# β-hydroxybutyrate restores social deficits by suppressing HDAC9 in the ACC of *Shank3B*-deficient mice

**DOI:** 10.1038/s44321-026-00491-9

**Published:** 2026-07-21

**Authors:** Erling Hu, Jialong Li, Chuchu Qi, Junye Ge, Yuanmeng Li, Qian Xue, Shengxi Wu, Wenting Wang

**Affiliations:** 1 Department of Neurobiology, School of Basic Medicine, Fourth Military Medical University, Xi’an, China; 2 The Shaanxi Province Key Laboratory of Brain Function Analysis and Modulation, Xi’an, China; 3 Guangdong Provincial Key Laboratory of Brain Function and Disease, Department of Physiology, School of Medicine, Shenzhen campus of Sun Yat-Sen University, Shenzhen, China; 4 School of Basic Medicine, Shaanxi University of Chinese Medicine, Xianyang, China

**Keywords:** Neuroscience

## Abstract

Autism spectrum disorder (ASD) is characterized by core deficits in social behavior, yet effective interventions remain limited. Ketogenic diet (KD) shows behavioral benefits in ASD, but the underlying mechanisms remain unclear. Here, using *Shank3B* knockout (KO) mice, we found that KD ameliorates social deficits and induces systemic ketosis with marked elevation of β-hydroxybutyrate (BHB) in KO mice. Oral BHB alone recapitulates KD’s prosocial effects, restoring social interaction and neuronal activity in the anterior cingulate cortex (ACC). Mechanistically, we identified HDAC9 as a region- specific epigenetic target upregulated in ACC neurons of *Shank3B* KO mice and suppressed by BHB. HDAC9 overexpression in ACC neurons induces social and synaptic deficits, while class IIa HDAC inhibition phenocopies BHB effects. BHB also restores dendritic complexity, excitatory transmission, and AMPA receptor expression. These findings uncover a metabolite-driven epigenetic mechanism linking ketogenic metabolism to the rescue of social behavior via the ACC, and identify HDAC9 as a potential therapeutic target for ASD-related social deficits.

## Synopsis

β-hydroxybutyrate was shown to improve ASD-related social deficits in a *Shank3B*-deficient ASD mouse model through suppression of HDAC9 in the anterior cingulate cortex.

- Social deficits in a *Shank3B*-deficient ASD mouse model were ameliorated by ketogenic diet treatment.
- β-hydroxybutyrate was identified as a key mediator linking ketogenic metabolism to behavioral and synaptic rescue.
- HDAC9 upregulation in the anterior cingulate cortex was associated with social and excitatory synaptic dysfunction.
- A metabolite–epigenetic pathway was defined that suggests HDAC9-related mechanisms as a potential therapeutic entry point for ASD-related social deficits.

## Abstract (web summary)

β-hydroxybutyrate was shown to improve ASD-related social deficits in a *Shank3B*-deficient ASD mouse model through suppression of HDAC9 in the anterior cingulate cortex.

## Graphical abstract

**Figure:**
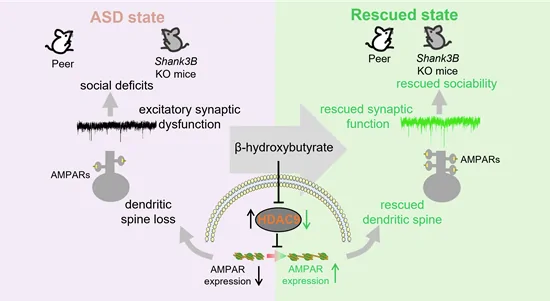

**Figure:**
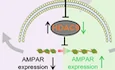

The paper explained **Problem**

Autism spectrum disorder (ASD) lacks effective mechanism-based treatments for core social deficits. Although ketogenic diets have shown potential benefits, the biological basis of these effects remains unclear, limiting the development of more practical targeted therapies.

**Results**

Using a *Shank3B*-deficient ASD mouse model, we identified β-hydroxybutyrate (BHB), a major ketone body produced by the ketogenic diet, as a key mediator of social rescue. BHB improved social behavior, restored excitatory synaptic structure and function in the anterior cingulate cortex, and reduced the abnormal increase of HDAC9, an epigenetic regulator linked here to social dysfunction in this model.

**Impact**

These findings define a metabolite–epigenetic pathway linking ketogenic metabolism to improvement of ASD-related social deficits. This work suggests that HDAC9-related mechanisms may represent a potential therapeutic avenue for social dysfunction associated with ASD.

## Introduction

Autism spectrum disorder (ASD) is a complex neurodevelopmental condition broadly characterized by deficits in social interaction and communication and restricted and repetitive patterns of behavior, interests, or activities (Quaak et al, 2013). Symptoms typically emerge in early childhood and display considerable clinical heterogeneity, presenting significant challenges for developing therapeutic interventions. While behavioral and language-based interventions remain the cornerstone of ASD management, their effectiveness in mitigating core symptoms, particularly in older individuals, is often incomplete and variable (Lord et al, 2018). This limited efficacy underscores a critical gap in our treatment options and emphasizes the need for novel, mechanism-based strategies that target the underlying neurobiology of ASD.

Emerging evidence has linked ASD to metabolic disturbances in the brain (Giulivi et al, 2010; Khaliulin et al, 2025), which has led to growing interest in metabolic interventions for this condition. Among these, a ketogenic diet (KD), defined as a high-fat, low-carbohydrate regimen that induces a state of nutritional ketosis, has shown promise in alleviating certain behavioral symptoms associated with ASD. Preclinical models and limited clinical observations suggest that a KD may exert positive effects for these individuals, particularly in the domain of social functioning (Garcia-Juarez et al, 2025; Kasprowska-Liskiewicz et al, 2017; Lee et al, 2018; Mohrle et al, 2024). However, long-term adherence to KD is challenging in the ASD population due to feeding difficulties, food selectivity, and gastrointestinal comorbidities (Schreck and Williams, 2006). Identifying the active metabolites responsible for KD’s behavioral benefits could overcome practical constraints and enable targeted, mechanism-driven therapeutics.

Metabolic products generated during the ketogenic diet play a crucial role in regulating neural function and behavior (Zhu et al, 2022). For example, certain metabolites can influence epigenetic regulation, neuroinflammation, and synaptic plasticity, thereby improving neural function (Gomora-Garcia et al, 2023; Lund et al, 2015; Newman and Verdin, 2017). These properties make these specific metabolites potential therapeutic targets, especially for complex disorders like ASD, which involve multiple biological processes (Ayoub, 2024; Iyer et al, 2024; Puchalska and Crawford, 2017). While there is evidence that metabolites produced during KD may contribute to the improvement of ASD symptoms, the specific metabolites and their mechanisms of action are not fully understood.

Given that ASD involves multi-system biological and neural disruptions, identifying how ketogenic metabolites modulate social-critical brain hubs is essential to uncovering their therapeutic mechanisms. Previous reports have identified several brain regions involved in social functions, such as the hippocampus, anterior cingulate cortex (ACC), striatum, and paraventricular nucleus (Sato et al, 2023). For example, ACC, a core hub for processing social salience, empathy, and social decision-making (Apps et al, 2016; Balsters et al, 2017), is consistently linked to social deficits in ASD (Urbain et al, 2015; Willbrand et al, 2026). In line with these clinical findings, our previous work demonstrated that ACC dysfunction drives social impairments in *Shank3B* KO mice, while selective activation of ACC pyramidal neurons rescues these deficits (Guo et al, 2019), highlighting the ACC as a key therapeutic target for ASD’s core social symptoms.

In the present study, we first confirmed that the ketogenic diet (KD) effectively improves social behavior in *Shank3B* knockout (KO) mice, a validated model of ASD (Peca et al, 2011). Subsequent analyses identified β-hydroxybutyrate (BHB) as a key metabolic product responsible for the prosocial effects of KD in *Shank3B* KO mice. At the cellular level, BHB-mediated behavioral improvements were accompanied by significant enhancements in the structure and functions of excitatory synapses on pyramidal neurons within the ACC. Mechanistically, we identified the epigenetic regulator HDAC9, which was selectively upregulated in the ACC of the KO mice, as the primary molecular target suppressed by BHB. Furthermore, both pharmacological inhibition of class IIa HDACs and direct neuronal overexpression of this molecule validated its pivotal role in social and synaptic deficits. Together, these findings provide mechanistic insights into the therapeutic effects of a KD and highlight BHB-HDAC9 signaling as a promising strategy for ameliorating core symptoms in individuals with ASD.

## Results

### A ketogenic diet alleviates social deficits in *Shank3B* KO mice

**Figure 1:**
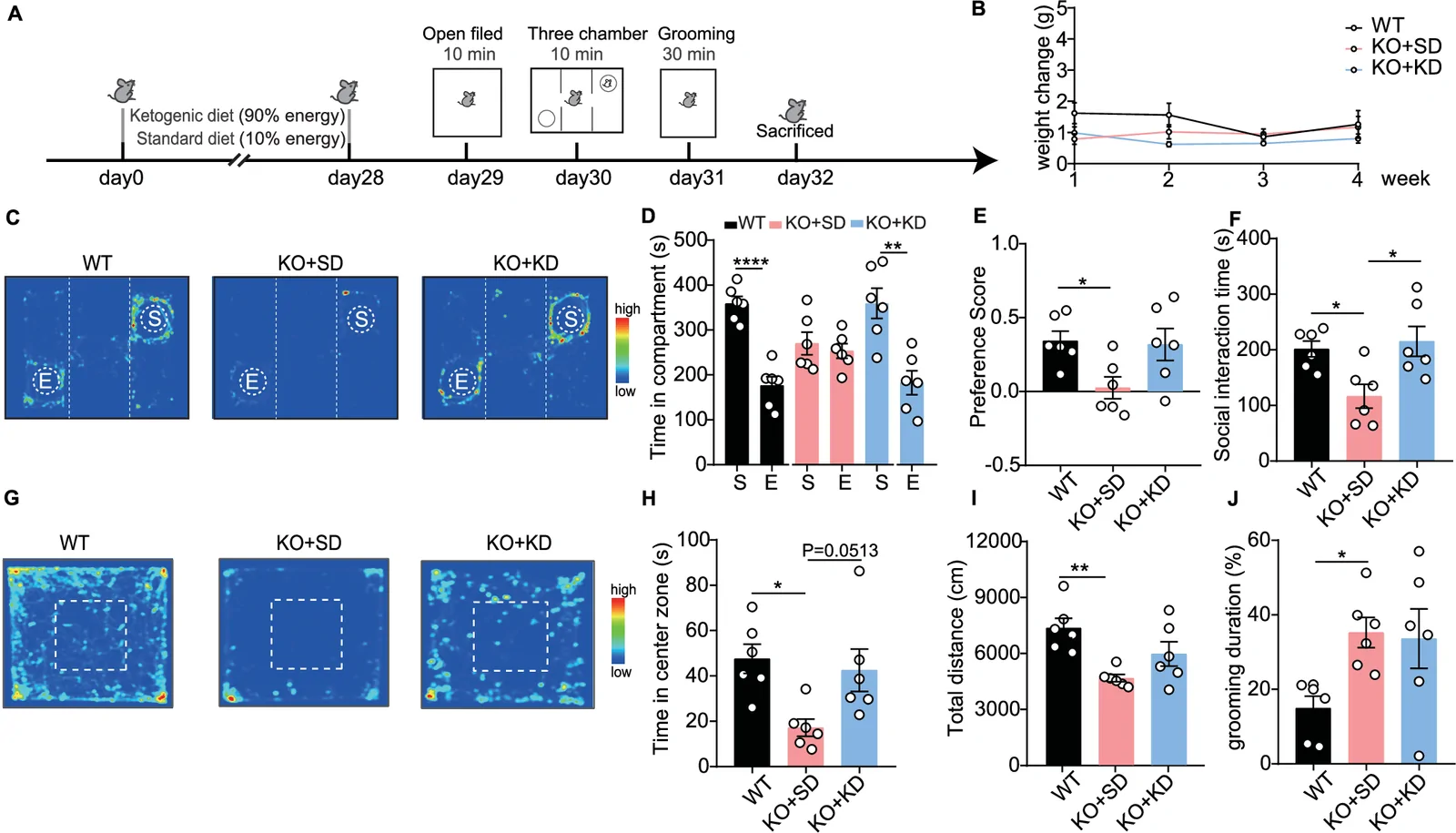
A ketogenic diet can effectively alleviate social deficits in *Shank3B* KO mice. (**A**) Schematic diagram of ketogenic diets in mice. (**B**) Changes in weight among WT mice and *Shank3B* KO mice fed a KD or a SD during the treatment period. Representative movement traces of mice (**C**) and comparisons of the time spent in the compartment (**D**), preference score (**E**), and social interaction time with the social mouse (**F**) among WT mice and *Shank3B* KO mice fed a KD or a SD in the three-chamber test (S social mouse, E empty cup). *n* = 6 mice in each group. Data are presented as mean ± SEM. Statistical analysis in (D) was performed using a two‑tailed Student’s *t* test. P_WT(S) vs. WT(E)_  < 0.0001, P_KO+KD(S) vs. KO+KD(E)_  =  0.0020. One‑way ANOVA followed by Tukey’s multiple comparisons test was used for statistical analysis in (**E**). P_KO+SD vs. WT_ =  0.0327, P_KO+SD vs. KO+KD_ =  0.0653. One‑way ANOVA followed by Tukey’s multiple comparisons test was used for statistical analysis in (**F**). P_KO+SD vs. WT_ =  0.0342, P_KO+SD vs. KO+KD_ =  0.0143. Representative movement traces of mice (**G**) and comparison of the time spent in the center zone (**H**) and total distance traveled (**I**) among WT mice and *Shank3B* KO mice fed a KD or a SD in the open-field test. *n* = 6 mice in each group. Data are presented as mean ± SEM. One‑way ANOVA followed by Tukey’s multiple comparisons test was used for statistical analysis in (**H**). P_KO+SD vs. WT_ =  0.0189. One‑way ANOVA followed by Tukey’s multiple comparisons test was used for statistical analysis in (**I**). P_KO+SD vs. WT_ =  0.0043. (**J**) Total duration of grooming behavior among WT mice and *Shank3B* KO mice fed a KD or a SD for 30 min of free movement. *n* = 6 mice in each group. Data are presented as mean ± SEM. One‑way ANOVA followed by Tukey’s multiple comparisons test was used for statistical analysis. P_KO+SD vs. WT_ =  0.0161. Source data are available online for this figure.

To evaluate the therapeutic potential of a KD on core autism-related behaviors, we subjected *Shank3B* KO mice to a four-week dietary regimen consisting of either a high-fat, low-carbohydrate KD or a matched SD (Table EV1). The KD regimen was well-tolerated, as evidenced by comparable body weight gain between the diet groups, indicating the absence of overt metabolic stress (Fig. 1A,B). As expected, compared with wild-type (WT) control mice, SD-fed *Shank3B* KO mice exhibited profound impairments in sociability, as they spent significantly less time in the social chamber and yielded a reduced social preference index. Strikingly, KD administration significantly ameliorated these deficits, with the KD-fed KO mice exhibiting restored social chamber occupancy, positive preference scores, and social interaction time that were indistinguishable from those of WT mice (Fig. 1C–F). Furthermore, in the open-field test, KD-fed KO mice spent more time in the central area, indicating a concurrent reduction in anxiety-like behavior (Fig. 1G,H). Although the locomotor activity of the KO mice was lower than that of the WT mice, the KD did not alter locomotion, suggesting a selective effect of the KD on social and affective domains (Fig. 1I). Importantly, while the self-grooming behavior of the SD-KO mice was markedly elevated, the KD failed to reverse this alteration (Fig. 1J). Collectively, these findings demonstrate that sustained KD selectively rescues social deficits and anxiety-like behaviors in *Shank3B* KO mice but not stereotypy or locomotion deficits.

### Ketogenic diet induces a robust systemic ketogenic metabolic state in *Shank3B* KO mice

**Figure 2:**
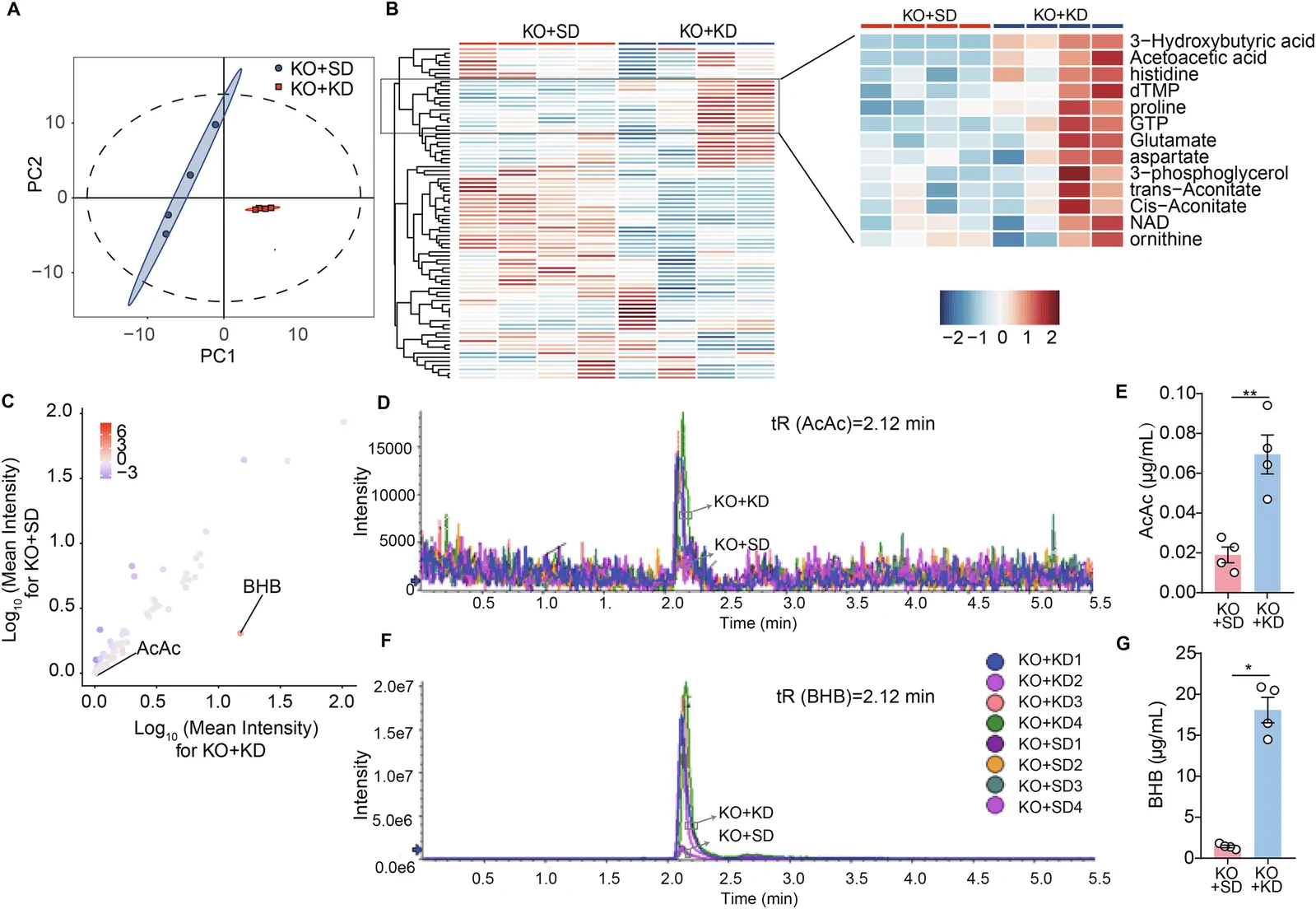
Ketogenic diet induces a robust systemic ketogenic metabolic state in *Shank3B* KO mice. (**A**) Principal component analysis (PCA) of targeted serum metabolomic profiles showing clear separation between *Shank3B* KO mice fed a standard diet (KO + SD) and those fed a ketogenic diet (KO + KD) according to LC–MS measurements. (**B**) Heatmap of significantly altered serum metabolites between KO + SD and KO + KD groups, visualized as row-wise Z-scores. Metabolites were selected based on statistical significance. (**C**) Differential abundance analysis illustrating the marked upregulation of β-hydroxybutyrate (BHB) and acetoacetate (AcAc) in KO + KD mice compared with KO + SD controls. (**D**) Representative extracted ion chromatograms of AcAc detected by LC–MS in serum samples from KO + SD and KO + KD mice. (**E**) Absolute quantification of circulating AcAc levels in KO + SD and KO + KD mice. *n* = 4 mice in each group. Data are presented as mean ± SEM. Statistical significance was determined by an unpaired two-tailed Student’s *t* test. *P*  =  0.0031. (**F**) Representative extracted ion chromatograms of BHB detected by LC–MS in serum samples from KO + SD and KO + KD mice. (**G**) Absolute quantification of circulating BHB levels in KO + SD and KO + KD mice. *n* = 4 mice in each group. Data are presented as mean ± SEM. Statistical significance was determined by the Mann–Whitney *U* test. *P*  =  0.0286. Source data are available online for this figure.

Targeted serum metabolomic profiling was performed to determine whether KD feeding effectively induced a ketogenic metabolic state in KO mice. Principal component analysis (PCA) revealed a clear separation between KO mice fed a standard diet (KO + SD) and those fed a ketogenic diet (KO + KD), indicating a global remodeling of the circulating metabolic profile following KD intervention (Fig. 2A). Consistent with this global shift, hierarchical clustering analysis of significantly altered metabolites demonstrated a distinct metabolic signature associated with KD feeding (Fig. 2B). Among these metabolites, ketone bodies including β-hydroxybutyrate (BHB) and acetoacetate (AcAc) were prominently elevated, whereas several amino acids and central carbon metabolism–related intermediates exhibited coordinated changes, reflecting a systemic reprogramming of energy metabolism under ketogenic conditions. Differential abundance analysis further identified BHB and AcAc as among the most strongly upregulated metabolites in KO + KD mice compared with KO + SD controls (Fig. 2C). Absolute quantification confirmed that circulating AcAc levels were elevated by approximately 3.64-fold (Fig. 2D,E), while BHB levels were increased by approximately 12.6-fold following KD feeding (Fig. 2F,G). Together, these findings demonstrate that ketogenic diet feeding induces a robust systemic ketogenic metabolic state in KO mice, characterized by a marked elevation of circulating ketone bodies.

### β-hydroxybutyrate mimics the therapeutic effects of a ketogenic diet in *Shank3B* KO mice

**Figure 3:**
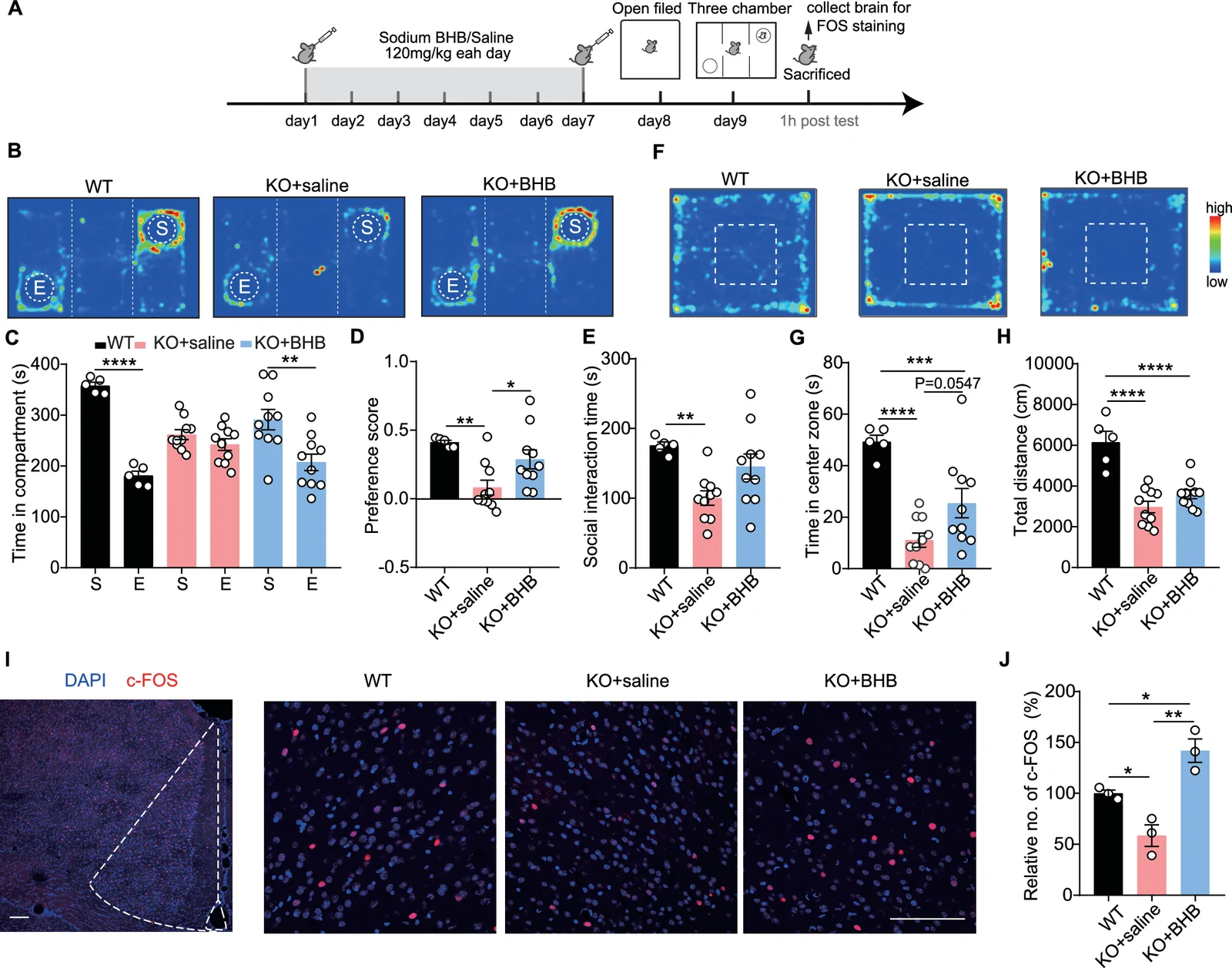
Sodium β-hydroxybutyrate administration ameliorates social behavior deficits in *Shank3B* KO mice. (**A**) Schematic diagram of oral gavage administration of sodium BHB to mice. Representative movement traces of mice (**B**) and quantification of the time spent in the compartment (**C**), preference score (**D**), and social interaction time with the social mouse (**E**) among WT mice, saline-treated *Shank3B* KO mice, and BHB-treated *Shank3B* KO mice in the three-chamber test (S social mouse, E empty cup). *n* = 5 mice in WT group, *n* = 10 mice in KO+saline group, and *n* = 10 mice in KO + BHB group. Data are presented as mean ± SEM. A two‑tailed Student’s *t* test was used for statistical analysis in (**C**). P_WT(S) vs. WT(E)_  < 0.0001, P_KO+BHB(S) vs. KO+BHB(E)_  =  0.0043. One‑way ANOVA followed by Tukey’s multiple comparisons test was used for statistical analysis in (**D**). P_KO+saline vs. WT_ =  0.0069, P_KO+saline vs. KO+BHB_ =  0.0417. One‑way ANOVA followed by Tukey’s multiple comparisons test was used for statistical analysis in (**E**). P_KO+saline vs. WT_ =  0.0097. Representative movement traces of mice (**F**) and quantification of the time spent in the center zone (**G**) and total distance traveled (**H**) among WT mice, saline-treated *Shank3B* KO mice, and BHB-treated *Shank3B* KO mice in the open-field test. *n *= 5 mice in WT group, *n* = 10 mice in KO+saline group, and *n* = 10 mice in KO + BHB group. Data are presented as mean ± SEM. One‑way ANOVA followed by Tukey’s multiple comparisons test was used for statistical analysis in (**G**). P_KO+saline vs. WT_ < 0.0001, P_KO+saline vs. KO+BHB_ =  0.0547, P_WT vs. KO+BHB_ =  0.0080. One‑way ANOVA followed by Tukey’s multiple comparisons test was used for statistical analysis in (**H**). P_KO+saline vs. WT_ < 0.0001, P_WT vs. KO+BHB_ < 0.0001. (**I**) Representative images of social interaction-induced c-FOS expression in the ACC among WT mice, saline-treated *Shank3B* KO mice, and BHB-treated *Shank3B* KO mice. (**J**) Fold change in the number of c-FOS-positive cells in the ACC relative to the WT group. Scale bar, 100 μm. *n* = 3 mice in each group. Data are presented as mean ± SEM. One‑way ANOVA followed by Tukey’s multiple comparisons test was used for statistical analysis. P_KO+saline vs. WT_ =  0.0426, P_KO+saline vs. KO+BHB_ =  0.0016, P_WT vs. KO+BHB_ =  0.0412. Source data are available online for this figure.

Given that BHB was among the most strongly elevated metabolites following KD and its well-documented role in epigenetic regulation and neuroprotective function (Newman and Verdin, 2017), we next asked whether exogenous BHB administration alone would be sufficient to recapitulate the beneficial effects of KD on social behavior in KO mice. To directly test this possibility, sodium BHB was administered via oral gavage to 4 to 6-week-old *Shank3B* KO mice at a dose of 120 mg/kg once daily for 7 consecutive days (Fig. 3A). Strikingly, this short-term BHB treatment was sufficient to produce comparable prosocial effects of the chronic KD. In the three-chamber social test, BHB treatment restored social preference in KO mice, as indicated by significantly greater occupancy of the social chamber than the empty chamber and an increased social preference score compared with KO+saline mice. Social interaction time also showed an apparent recovery toward WT levels following BHB treatment, although this effect was less pronounced than the improvement in preference score (Fig. 3B–E). Furthermore, BHB partially alleviated anxiety-like behavior in the open-field test, increasing center-zone dwell time in KO mice. This anxiolytic-like effect occurred without a significant change in total distance traveled between KO+saline and KO + BHB mice, indicating that the behavioral improvement was unlikely to be driven by increased locomotor activity (Fig. 3F–H). To explore neural correlates associated with this behavioral rescue, we examined c-FOS induction in the anterior cingulate cortex (ACC), a brain region critically involved in social behavior. c-FOS expression was markedly reduced in the ACC of *Shank3B* KO mice compared to WT controls during the social interaction test. Importantly, BHB treatment increased c-Fos induction in the ACC to levels comparable to those observed in WT mice (Fig. 3I,J), suggesting enhanced neuronal activity in the ACC during social behavior. Collectively, these findings demonstrate that exogenous BHB administration may be sufficient to mimic key behavioral and neural effects of a ketogenic diet, supporting a central role for BHB in mediating KD-associated rescue of social deficits in *Shank3B* KO mice.

### HDAC9 is upregulated in the ACC of *Shank3B* KO mice and downregulated by β-hydroxybutyrate

**Figure 4:**
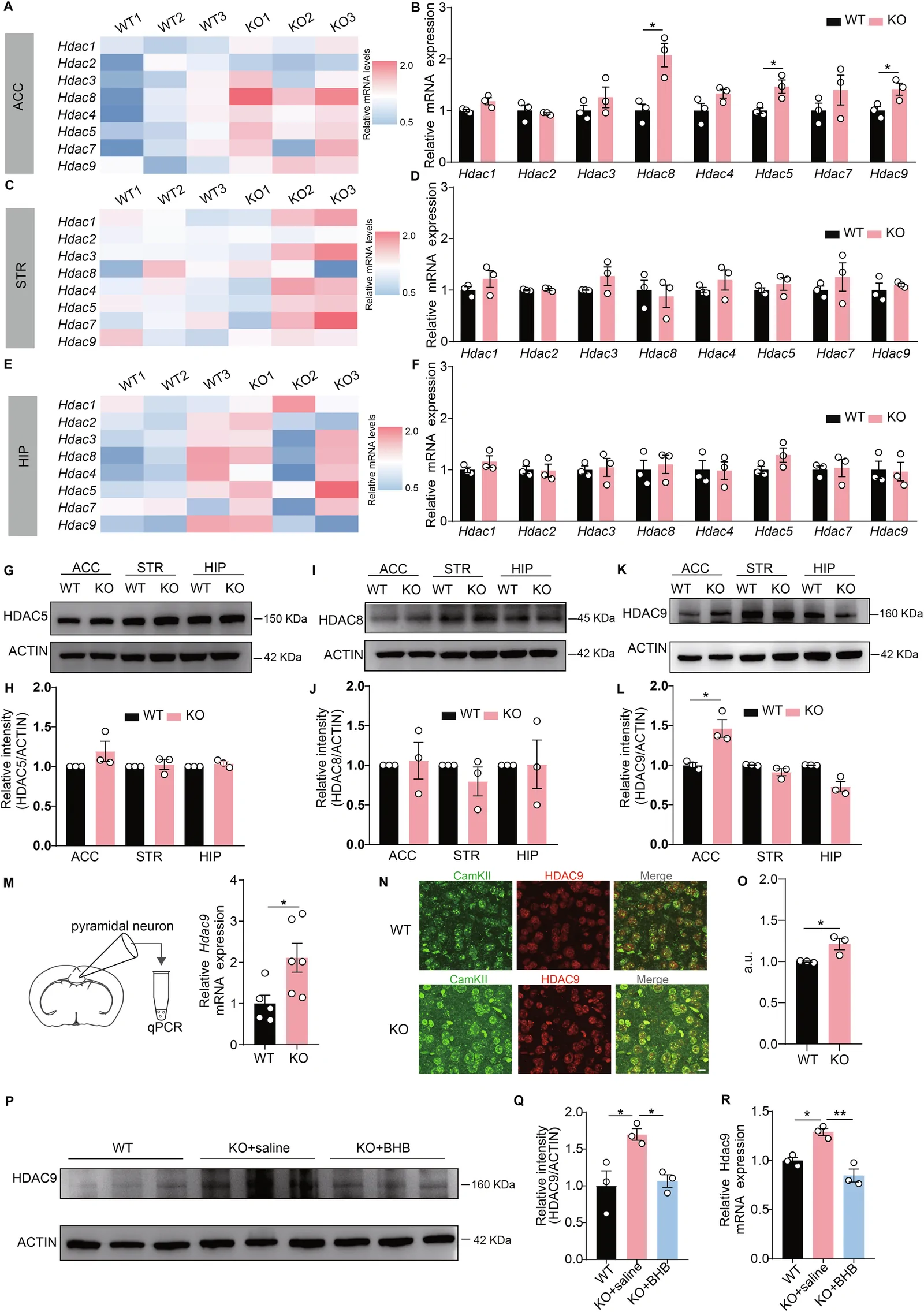
HDAC9 expression is significantly increased in the ACC of *Shank3B* KO mice. Heatmap (**A**) and quantitative analysis (**B**) of the mRNA levels of class I and class IIa HDACs in the ACC of WT and *Shank3B* KO mice. *n* = 3 mice per group. Data are presented as mean ± SEM. A two‑tailed Student’s *t* test was used for statistical analysis. P_WT(Hdac8) vs. KO(Hdac8)_  =  0.0148, P_WT(Hdac5) vs. KO(Hdac5)_  =  0.0299, P_WT(Hdac9) vs. KO(Hdac9)_  =  0.0427. Heatmap (**C**) and quantitative analysis (**D**) of the mRNA levels of class I and class IIa HDACs in the striatum (STR) of WT and *Shank3B* KO mice. *n* = 3 mice per group. Heatmap (**E**) and quantitative analysis (**F**) of the mRNA levels for class I and class IIa HDACs in the hippocampus (HIP) of WT and *Shank3B* KO mice. *n* = 3 mice per group. (**G**) Immunoblot for HDAC5 in the ACC, STR, and HIP. (**H**) Quantitative analysis of HDAC5 intensity relative to that of ACTIN. *n* = 3 mice per group. (**I**) Immunoblot for HDAC8 in the ACC, striatum, and hippocampus. (**J**) Quantitative analysis of HDAC8 intensity relative to that of ACTIN. *n* = 3 mice per group. (**K**) Immunoblot for HDAC9 in the ACC, striatum, and hippocampus. (**L**) Quantitative analysis of HDAC9 intensity relative to that of ACTIN. *n* = 3 mice per group. Data are presented as mean ± SEM. A two-tailed Student’s *t* test was used for statistical analysis. P_WT(ACC) vs. KO(ACC)_  =  0.0160. (**M**) Pyramidal neurons in the ACC were collected and subjected to single-cell PCR to assess the mRNA levels of *Hdac9*. Neurons from two mice were divided into five groups (WT) or 6 groups (KO), with 12–14 cells per group. Data are presented as mean ± SEM. A two‑tailed Student’s *t* test was used for statistical analysis. P_WT vs. KO_  =  0.0298. (**N**) Representative immunofluorescence images showing HDAC9 and CaMKII staining in the ACC. Scale bar, 10 μm. (**O**) Quantification of HDAC9 fluorescence intensity in CaMKII-positive neurons in the ACC of WT and KO mice. *n* = 3 mice per group. A two-tailed Student’s *t* test was used for statistical analysis. P_WT vs. KO_  =  0.0372. (**P**) Immunoblots for HDAC9 in the ACC of WT mice or KO mice treated with sodium BHB or saline. (**Q**) Quantification of HDAC9 intensity relative to that of ACTIN. *n* = 3 mice per group. Data are presented as mean ± SEM. One‑way ANOVA followed by Tukey’s multiple comparisons test was used for statistical analysis. P_KO+saline vs. WT_ =  0.0249, P_KO+saline vs. KO+BHB_ =  0.0374. (**R**) HDAC9 mRNA levels in the ACC of WT mice and KO mice treated with sodium BHB or saline. *n* = 3 mice per group. Data are presented as mean ± SEM. One‑way ANOVA followed by Tukey’s multiple comparisons test was used for statistical analysis. P_KO+saline vs. WT_ =  0.0116, P_KO+saline vs. KO+BHB_ =  0.0014. Source data are available online for this figure.

Given the known function of BHB as an endogenous inhibitor of class I/IIa histone deacetylases (HDACs) and the emerging role of epigenetic dysregulation in ASD, we sought to identify specific HDAC targets of BHB in the brain *of Shank3B* KO mice. Initial qPCR screening of the ACCs revealed elevated mRNA levels of several HDACs, including HDAC8 (class I), HDAC5, and HDAC9 (class IIa), in the KO mice compared with WT controls (Fig. 4A,B). Given previous reports of structural and functional abnormalities in the striatal neurons of *Shank3B* KO mice (Peca et al, 2011), we extended our analysis to the striatum and hippocampus to determine the regional specificity of this dysregulation. Notably, no significant changes in the expression of these HDACs were observed in these regions, indicating the presence of a selective epigenetic alteration within the ACC (Fig. 4C–F). Subsequent protein-level analysis confirmed that HDAC9 was uniquely and significantly upregulated in the ACC of the KO mice, whereas the HDAC5 and HDAC8 protein levels remained unchanged (Fig. 4G–L). This specific upregulation was also observed in excitatory neurons, as demonstrated by elevated HDAC9 mRNA in individually patched ACC pyramidal neurons by single-cell qPCR (Fig. 4M) and increased HDAC9 protein in CaMKII-positive neurons via immunohistochemistry (Fig. 4N,O). Importantly, the administration of sodium BHB, which we established rescued social deficits, effectively restored both the mRNA and protein expression of HDAC9 in the ACCs of *Shank3B* KO mice (Fig. 4P–R). Together, these findings indicate that HDAC9 is dysregulated in the ACC of *Shank3B* KO mice and its expression is restored by BHB treatment.

### HDAC9 overexpression in ACC neurons induces social deficits that are rescued through BHB treatment

**Figure 5:**
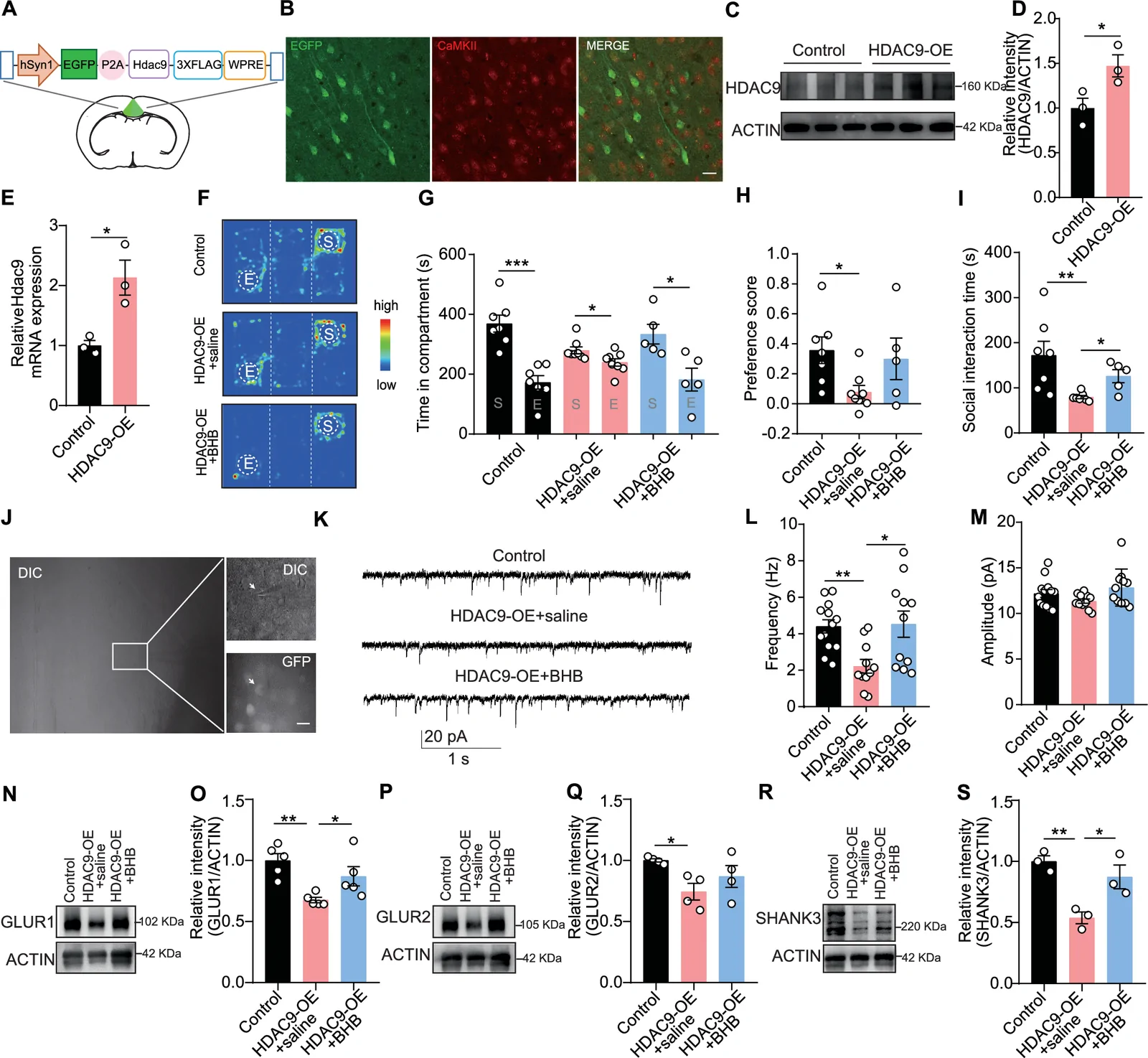
Sodium β-hydroxybutyrate rescues social deficits induced by HDAC9 overexpression in ACC neurons. (**A**) Schematic of the injection method used to overexpress HDAC9 in neurons in the ACC. (**B**) Representative immunohistochemical images EGFP colocaliztion with CaMKII in the ACC. Scale bar, 20 μm. (**C**) Immunoblots and (**D**) quantitative analysis of HDAC9 expression in the ACC of HDAC9-overexpressing mice and control mice. *n* = 3 mice per group. Data are presented as mean ± SEM. A two-tailed Student’s *t* test was used for statistical analysis. P_Control vs. HDAC9-OE_  =  0.0463. (**E**) *Hdac9* mRNA levels in the ACC of mice at 4 weeks post injection. *n* = 3 mice per group. Data are presented as mean ± SEM. A two-tailed Student’s *t* test was used for statistical analysis. P_Control vs. HDAC9-OE_  =  0.0202. Representative movement traces of mice (**F**) and quantification of the time spent in the compartment (**G**), preference score (**H**), and social interaction time with the social mouse (**I**) in the three-chamber test among control virus-injected mice, HDAC9-overexpressing mice treated with saline, and HDAC9-overexpressing mice treated with sodium BHB (S social mouse, E empty cup). *n* = 7 control group, *n* = 8 HDAC9+saline group, and *n* = 5 HDAC9 + BHB group. Data are presented as mean ± SEM. Two‑tailed Student’s *t* test or Mann–Whitney *U* test was used for statistical analysis in (**G**). P_Control(S) vs. Control (E)_  =  0.0002, P_HDAC9-OE+saline(S) vs. HDAC9-OE+saline(E)_  =  0.0166, P_HDAC9-OE+BHB(S) vs. HDAC9-OE+BHB(E)_  =  0.0171. One‑way ANOVA followed by Tukey’s multiple comparisons test was used for statistical analysis in (**H**). P_HDAC9-OE+saline vs. Control_ =  0.0332. Kruskal–Wallis test followed by Dunn’s multiple comparisons test was used for statistical analysis in (**I**). P_HDAC9-OE+saline vs. Control_ =  0.0021, P_HDAC9-OE+saline vs. HDAC9-OE+BHB_ =  0.0435. Representative DIC image (**J**) and representative mEPSC traces (**K**) recorded from ACC pyramidal neurons of control virus-injected mice, HDAC9-overexpressing mice treated with saline, and HDAC9-overexpressing mice treated with sodium BHB. Scale bar, 20 μm. Quantification of mEPSC frequency (**L**) and peak amplitudes (**M**). *n* = 13 cells from two mice in the control group, *n* = 11 cells from two mice in the HDAC9+saline group, and *n* = 11 cells from two mice in the HDAC9 + BHB group. Data are presented as mean ± SEM. Kruskal–Wallis test followed by Dunn’s multiple comparisons test was used for statistical analysis. P_HDAC9-OE +saline vs. Control_ =  0.0050, P_HDAC9-OE+saline vs. HDAC9-OE+BHB_ =  0.0159. (**N**) Immunoblots and (**O**) quantitative analysis of GLUR1 in the ACC of control virus-injected mice, HDAC9-overexpressing mice treated with saline, and HDAC9-overexpressing mice treated with sodium BHB. *n* = 5 mice per group. Data are presented as mean ± SEM. One‑way ANOVA followed by Bonferroni’s multiple comparisons test was used for statistical analysis. P_HDAC9-OE+saline vs. Control_ =  0.0018. P_HDAC9-OE+saline vs. HDAC9-OE+BHB_ =  0.0340. (**P**) Immunoblots and (**Q**) quantitative analysis of GLUR2 in the ACC of control virus-injected mice, HDAC9-overexpressing mice treated with saline, and HDAC9-overexpressing mice treated with sodium BHB. *n* = 4 mice per group. Data are presented as mean ± SEM. Kruskal–Wallis test followed by Bonferroni’s multiple comparisons test was used for statistical analysis. P_HDAC9-OE+saline vs. Control_ =  0.0215. (**R**) Immunoblots and (**S**) quantitative analysis for SHANK3 in the ACC of control virus-injected mice, HDAC9-overexpressing mice treated with saline, and HDAC9-overexpressing mice treated with sodium BHB. *n* = 3 mice per group. Data are presented as mean ± SEM. One‑way ANOVA followed by Tukey’s multiple comparisons test was used for statistical analysis. P_HDAC9-OE+saline vs. Control_ =  0.0073. P_HDAC9-OE+saline vs. HDAC9-OE+BHB_ =  0.0309. Source data are available online for this figure.

**Figure EV1:**
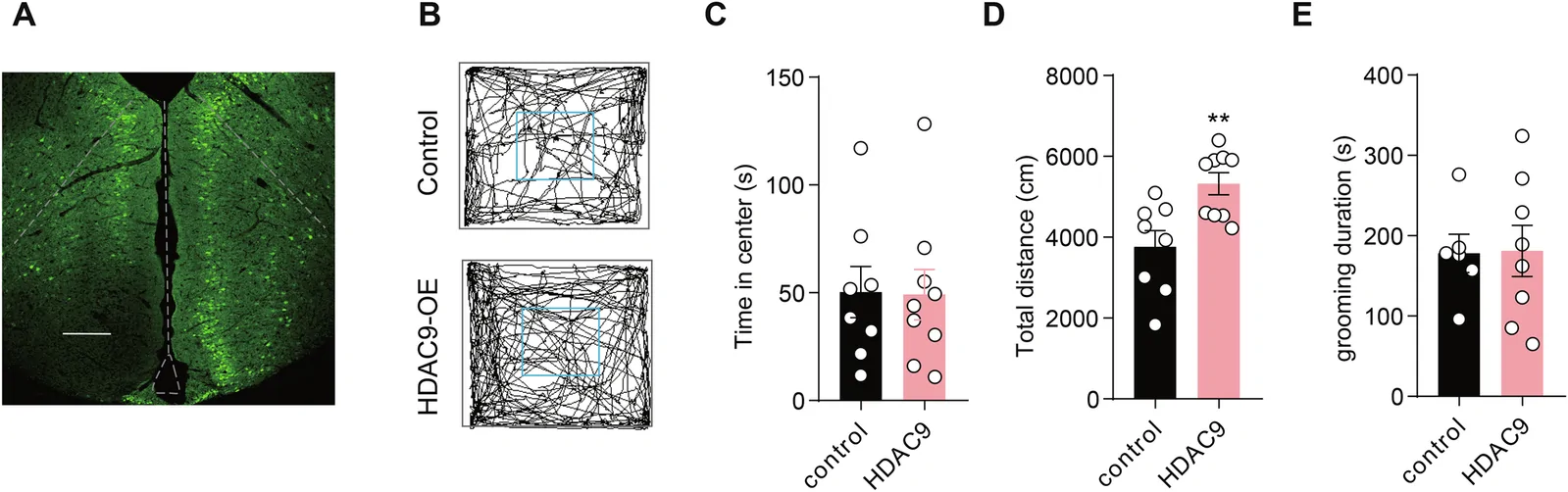
HDAC9 overexpression in ACC neurons does not affect anxiety-like or grooming behaviors. (**A**) Representative images showing viral expression and injection site localization in the ACC following stereotaxic injection of HDAC9-overexpressing AAV. Scale bar, 200 μm. (**B**) Representative movement traces of mice in the open-field test at 4 weeks after injection of control virus or HDAC9-overexpression virus into the ACC. Quantification of center zone dwell time (**C**) and total distance traveled (**D**) in the open-field test of mice injected with control virus or HDAC9-overexpression virus. n = 8 mice in the control group and 9 mice in the HDAC9-OE group. Data are presented as mean ± SEM. A two‑tailed Student’s *t* test was used for statistical analysis. *P* = 0.0052. (**E**) Quantification of grooming duration in mice at 4 weeks after injection of control virus or HDAC9-overexpression virus. *n* = 6 mice in the control group and 8 mice in the HDAC9-OE group. Data are presented as mean ± SEM. Source data are available online for this figure.

**Figure EV2:**
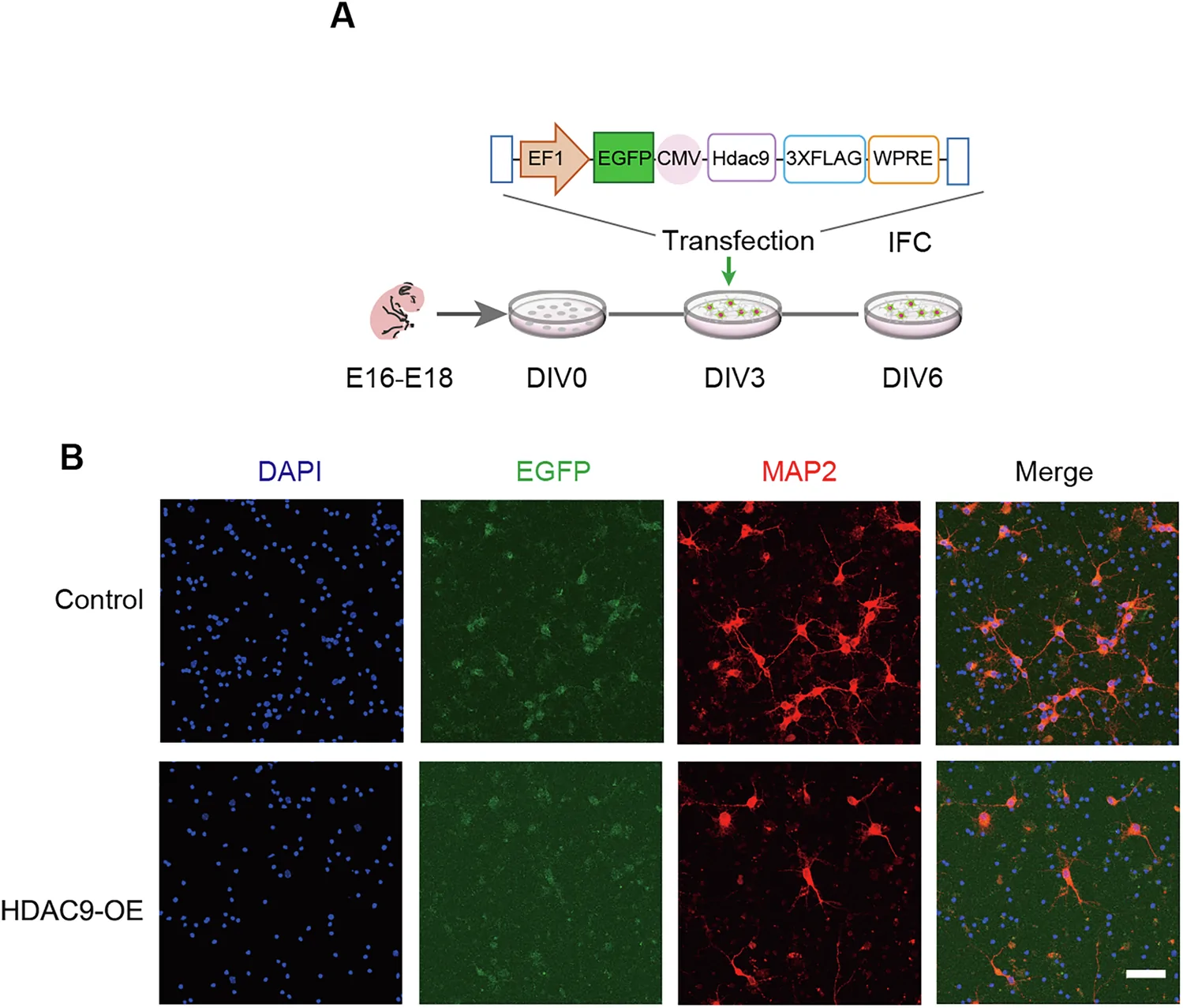
HDAC9 overexpression impairs the growth of cultured cortical neurons. (**A**) Timeline of the transfection of cultured cortical neurons with control lentivirus or HDAC9-overexpressing lentivirus. (**B**) Representative immunofluorescence images of cultured cortical neurons transfected with control lentivirus or HDAC9-overexpressing lentivirus. Scale bar, 50 μm. Source data are available online for this figure.

To establish a direct causal link between HDAC9 upregulation and autism-like pathophysiology, we asked whether targeted overexpression of HDAC9 in ACC neurons alone was sufficient to provoke core behavioral and synaptic deficits. We achieved selective overexpression via bilateral injection of AAV2/9-hSyn-EGFP-P2A-Hdac9-3XFLAG-WPRE (Fig. 5A). Immunostaining confirmed HDAC9 protein overexpression in CaMKII-positive excitatory neurons, and molecular analyses of ACC tissue showed elevated HDAC9 at both mRNA and protein levels (Figs. EV1A and  5B–E). Four weeks after viral injection, mice overexpressing HDAC9 exhibited impaired sociability compared with control mice, as evidenced by a reduced social preference score and decreased direct social interaction time (Fig. 5F–I). These findings indicate that HDAC9 overexpression in ACC neurons is sufficient to partially recapitulate the social deficits observed in *Shank3B* KO mice. However, the behavioral impact was specific, as HDAC9 overexpression did not alter anxiety-like (Fig. EV1B–D) or repetitive grooming behaviors (Fig. EV1E). Notably, BHB treatment ameliorated these HDAC9-OE-induced social deficits, increasing social interaction time and shifting the social preference score toward control levels (Fig. 5F–I). To define the underlying synaptic mechanisms, we performed a multilevel analysis. Cultured neurons were transduced with lentivirus overexpressing HDAC9 at DIV3, and morphological analysis was performed 3 days later (Fig. EV2A). Under these conditions, HDAC9 overexpression led to simplified dendritic arborization and impaired network connectivity (Fig. EV2B). Consistent with the behavioral abnormalities, electrophysiological recordings from the ACC pyramidal neurons showed that HDAC9 overexpression significantly decreased mEPSCs frequency, while mEPSC amplitude remained largely unchanged. These results suggest that HDAC9 overexpression mainly disrupts excitatory synaptic event frequency rather than postsynaptic response amplitude. BHB treatment effectively rescued the reduction in mEPSC frequency without significantly affecting mEPSC amplitude (Fig. 5J–M). Western blot analysis further revealed that HDAC9 overexpression downregulated the levels of the postsynaptic proteins GluR1, GluR2, and SHANK3 in ACC tissue. Notably, BHB administration restored GluR1 and SHANK3 expression and produced a partial recovery of GluR2 expression (Fig. 5N–S). Together, these results demonstrate that HDAC9 upregulation in the ACC neurons is sufficient to induce social and synaptic deficits that partially resemble key features of the *Shank3B* KO phenotype, and that BHB treatment can mitigate these HDAC9-driven deficits.

### Sodium BHB restores structural and functional synaptic impairments in *Shank3B* KO ACC neurons

**Figure 6:**
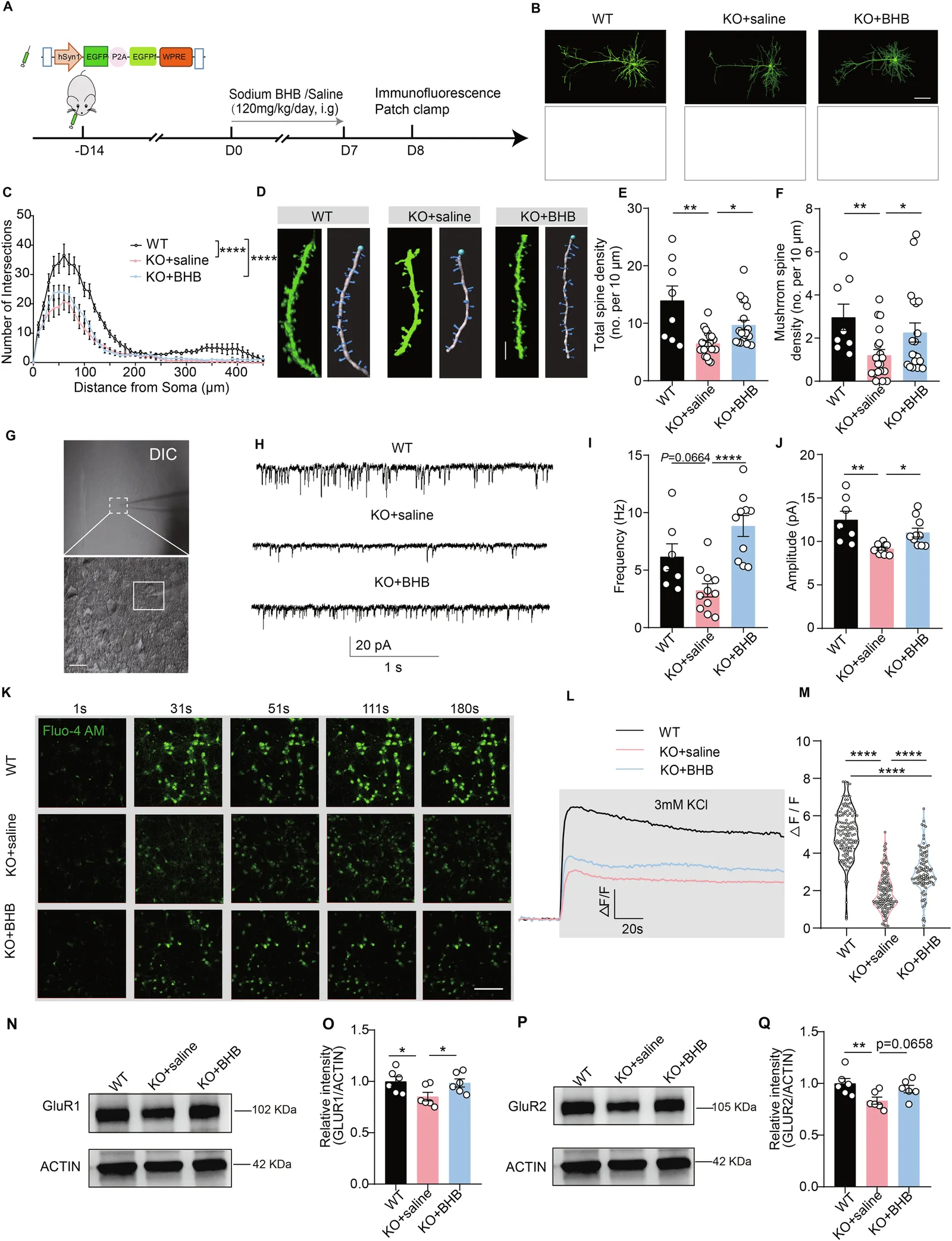
Sodium β-hydroxybutyrate rescues the dendritic structure and synaptic function of ACC neurons in *Shank3B* KO mice. (**A**) Schematic of the retroorbital injection method used to sparsely label neurons, followed by oral gavage administration of sodium BHB in mice for another 7 days after a 14-day interval. (**B**) Representative pyramidal neurons in the ACC of WT mice and *Shank3B* KO mice treated with sodium BHB or saline. Scale bar, 100 μm. (**C**) Summary of Sholl analysis of ACC pyramidal neurons. *n* = 10 neurons from 3 WT mice, *n* = 11 neurons from 4 KO+saline mice, and *n* = 13 neurons from 4 KO + BHB mice. Two‑way ANOVA followed by Tukey’s multiple comparisons test was used for statistical analysis. P_WT vs. KO+saline_ ≤  0.0001, P_WT vs. KO+BHB_ ≤  0.0001. (**D**) Representative confocal stacks and three-dimensional reconstruction images of the basal dendrites of ACC pyramidal neurons obtained from WT mice and *Shank3B* KO mice treated with sodium BHB or saline. Scale bar, 5 μm. Summary of total spine density (**E**) and mushroom spine density (**F**) of the basal dendrites of ACC pyramidal neurons. *n* = 8 dendrites from 3 WT mice, *n* = 19 dendrites from 3 KO+saline mice, and *n* = 19 dendrites from 3 KO + BHB mice. Data are presented as mean ± SEM. Kruskal–Wallis test followed by Dunn’s multiple comparisons test was used for statistical analysis. P_WT(Total spine density) vs. KO+saline(Total spine density)_ = 0.0058, P_KO+saline(Total spine density) vs. KO+BHB(Total spine density)_  = 0.0108. P_WT(Mushroom spine density) vs. KO+saline(Mushroom spine density)_ =  0.0032, P_KO+saline(Mushroom spine density) vs. KO+BHB(Mushroom spine density)_  = 0.0468. Representative DIC image (**G**) and representative mEPSC traces (**H**) recorded from ACC pyramidal neurons of WT mice or *Shank3B* KO mice treated with sodium BHB or saline. Scale bar, 20 μm. Quantification of mEPSC frequency (**I**) and peak amplitudes (**J**). *n* = 7 cells from 2 WT mice, *n* = 11 cells from 2 KO+saline mice, and *n* = 10 cells from 2 KO + BHB mice. Data are presented as mean ± SEM. One‑way ANOVA followed by Bonferroni’s multiple comparisons test was used for statistical analysis in (**I**). P_WT vs. KO+saline_ =  0.0664, P_KO+saline vs. KO+BHB_ = 0.0001. Kruskal–Wallis test followed by Dunn’s multiple comparisons test was used for statistical analysis in (J). P_WT vs. KO+saline_ =  0.0011, P_KO+saline vs. KO+BHB_ = 0.0236. (**K**–**M**) Calcium response of cultured cortical neurons from WT mice or *Shank3B* KO mice treated with or without sodium BHB. Quantification was based on peak ΔF/F after KCl stimulation. *n* = 133 cells in WT group, *n* = 140 cells in KO+saline group and *n* = 103 cells in KO + BHB group from 3 independent experiments. Data are presented as mean ± SEM. Kruskal–Wallis test followed by Dunn’s multiple comparisons test was used for statistical analysis. P_WT vs. KO+saline_ ≤  0.0001, P_KO+saline vs. KO+BHB_ ≤  0.0001, P_WT vs. KO+BHB_ ≤  0.0001. Scale bar, 100 μm. (**N**) Immunoblot and (**O**) quantitative analysis of GLUR1 in the ACC of WT mice or KO mice treated with sodium BHB or saline. *n* = 6 mice per group. Data are presented as mean ± SEM. One‑way ANOVA followed by Bonferroni’s multiple comparisons test was used for statistical analysis P_WT vs. KO+saline_ =  0.0259, P_KO+saline vs. KO+BHB_ = 0.0421. (**P**) Immunoblot and (**Q**) quantitative analysis of GLUR2 in the ACC of WT mice or KO mice treated with sodium BHB or saline. *n* = 6 mice per group. Data are presented as mean ± SEM. One‑way ANOVA followed by Bonferroni’s multiple comparisons test was used for statistical analysis. P_WT vs. KO+saline_ =  0.0099, P_KO+saline vs. KO+BHB_ = 0.0658. Source data are available online for this figure.

**Figure EV3:**
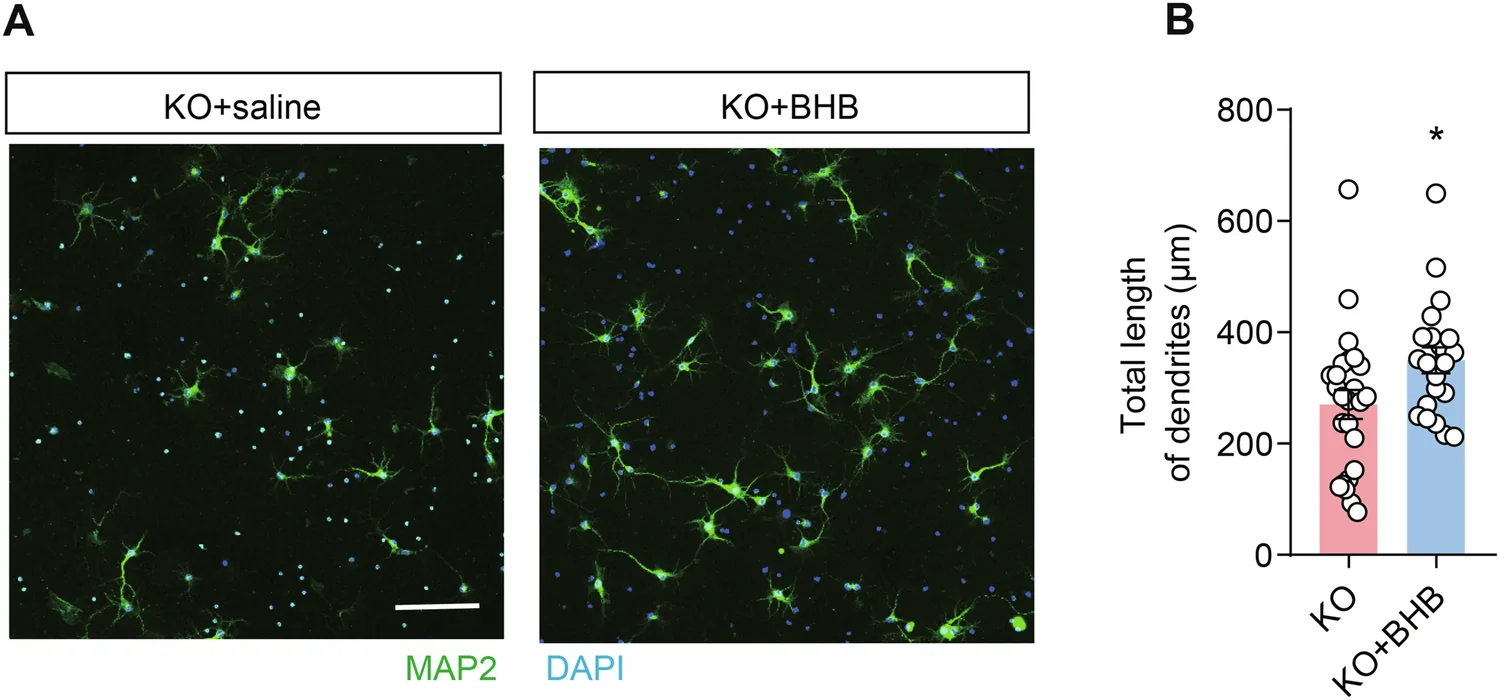
Sodium BHB administration increases dendritic length in *Shank3B* KO cortical neurons. (**A**) Representative immunofluorescence images showing DAPI and MAP2 staining in cultured cortical neurons from *Shank3B* KO mice treated with saline or 2 mM sodium BHB. (**B**) Quantification of total dendritic length in *Shank3B* KO cortical neurons treated with saline or 2 mM sodium BHB. *n* = 25 cells in the KO+Saline group, and *n* = 21 cells in the KO + BHB group. Data are presented as mean ± SEM. A two‑tailed Student’s *t* test was used for statistical analysis. *P* = 0.0298. Source data are available online for this figure.

**Figure EV4:**
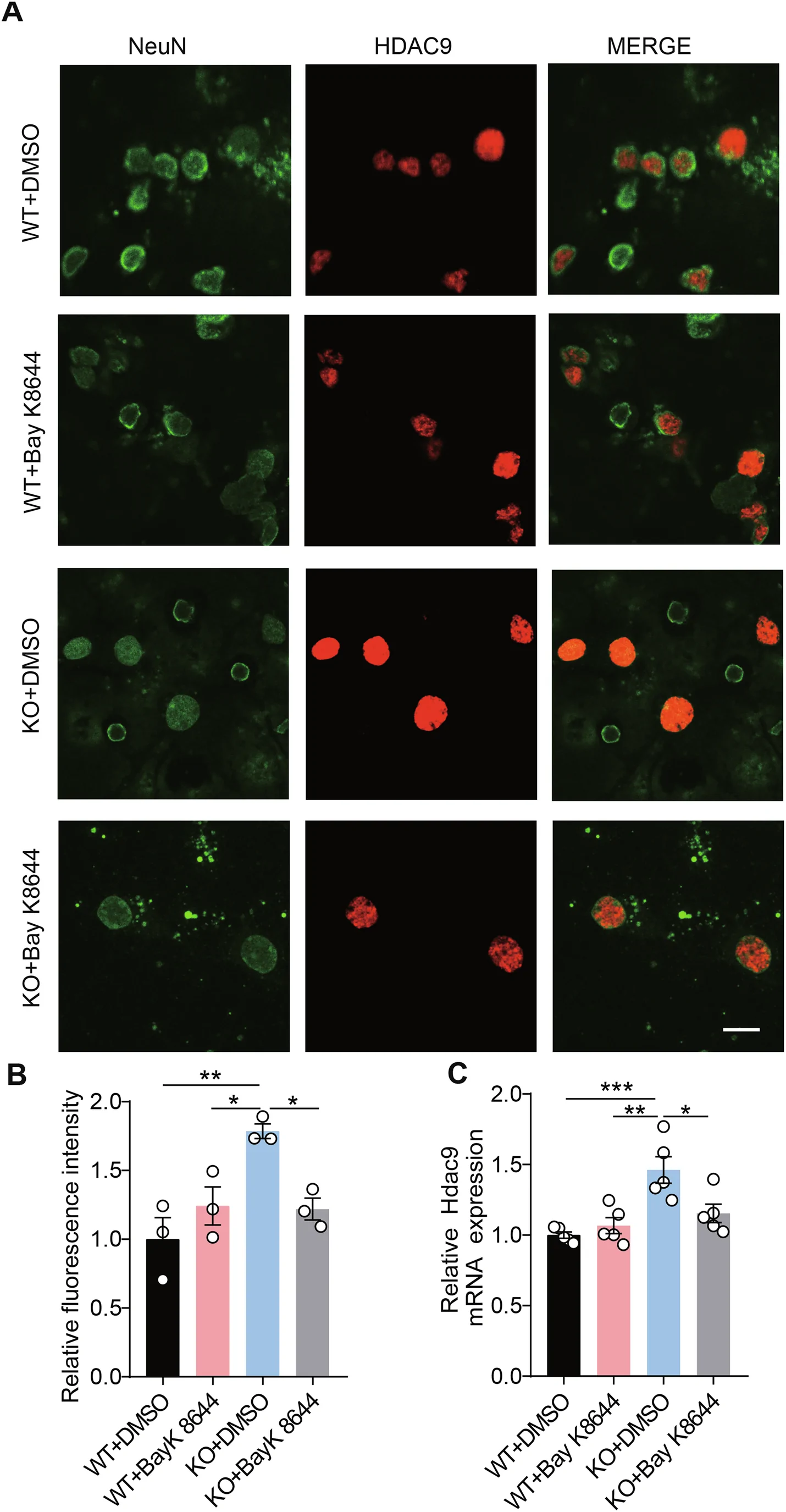
Bay K8644 treatment reduces HDAC9 expression in *Shank3B* KO cultured cortical neurons. (**A**) Representative immunofluorescence images showing NeuN and HDAC9 staining in cultured cortical neurons from WT and *Shank3B* KO mice treated with vehicle (DMSO) or the L‑type calcium channel agonist Bay K8644 (2 μM) for 12 h. Scale bar = 10 μm. (**B**) Quantification of the relative fluorescence intensity of HDAC9 per cell from each group. *n* = 3 slices with 36 neurons in the WT + DMSO group, *n* = 3 slices with 49 neurons in the WT+ Bay K8644 group, *n* = 3 slices with 33 neurons in the KO + DMSO group, and *n* = 3 slices with 19 neurons in the KO+ Bay K8644 group. Data are presented as mean ± SEM. One‑way ANOVA followed by Bonferroni’s multiple comparisons test was used for statistical analysis. P_WT+DMSO vs. KO+DMSO_ =  0.0021, P_KO+DMSO vs. WT+Bay K8644_ =  0.0163, P_KO+DMSO vs. KO+Bay K8644_ = 0.0133. (**C**) Relative mRNA expression of HDAC9 measured by quantitative real‑time PCR in cultured cortical neurons under the same treatment conditions. *n* = 5 independent biological samples each group. Data are presented as mean ± SEM. One‑way ANOVA followed by Tukey’s multiple comparisons test was used for statistical analysis. P_WT+DMSO vs. KO+DMSO_ =  0.0006, P_KO+DMSO vs. WT+Bay K8644_ =  0.0028, P_KO+DMSO vs. KO+Bay K8644_ = 0.0190. Source data are available online for this figure.

We next tested whether BHB could reverse intrinsic synaptic deficits in *Shank3B* KO mice. Using sparse labeling with AAV9-hSyn-EGFP-P2A-EGFPf, which was developed in our own laboratory (Chen et al, 2020), we visualized ACC pyramidal neurons and found that BHB-treated KO mice displayed significantly increased dendritic complexity, along with higher densities of total and mushroom-type dendritic spines compared with saline-treated KO mice (Fig. 6A–F). Consistent with these in vivo findings, cultured cortical neurons treated with 2 mM sodium BHB for 3 days displayed increased dendritic length (Fig. EV3A,B). Whole-cell patch-clamp recordings further revealed that BHB treatment significantly increased both mEPSCs frequency and amplitude in ACC pyramidal neurons, indicating improved excitatory synaptic transmission (Fig. 6G–J). In parallel, calcium imaging experiments revealed enhanced Ca²⁺ responses upon stimulation with 3 mM KCl in cultured *Shank3B* KO neurons treated with BHB (Fig. 6K–M). To further examine whether enhanced Ca²⁺ signaling could influence HDAC9 expression, we treated neurons with the L-type Ca²⁺ channel agonist Bay K8644. Bay K8644 markedly reduced the elevated HDAC9 immunofluorescence intensity (Fig. EV4A,B) and Hdac9 mRNA expression in *Shank3B* KO neurons, while having little effect in WT neurons (Fig. EV4C). These results suggest that impaired Ca²⁺-dependent signaling may contribute to HDAC9 upregulation in *Shank3B* KO neurons, and that restoring neuronal Ca²⁺ responsiveness is associated with suppression of HDAC9 expression. Moreover, western blot analyses further demonstrated that BHB restored GluR1 expression in the ACC, whereas GluR2 showed a non-significant trend toward recovery (*P* = 0.0658; Fig. 6N–Q). Collectively, these results indicate that BHB treatment ameliorates structural and functional deficits at excitatory synapses in the ACC of *Shank3B* KO mice.

### Systemic administration of the class II HDAC inhibitor TMP269 effectively rescued synaptic and social deficits in *Shank3B* KO mice

**Figure 7:**
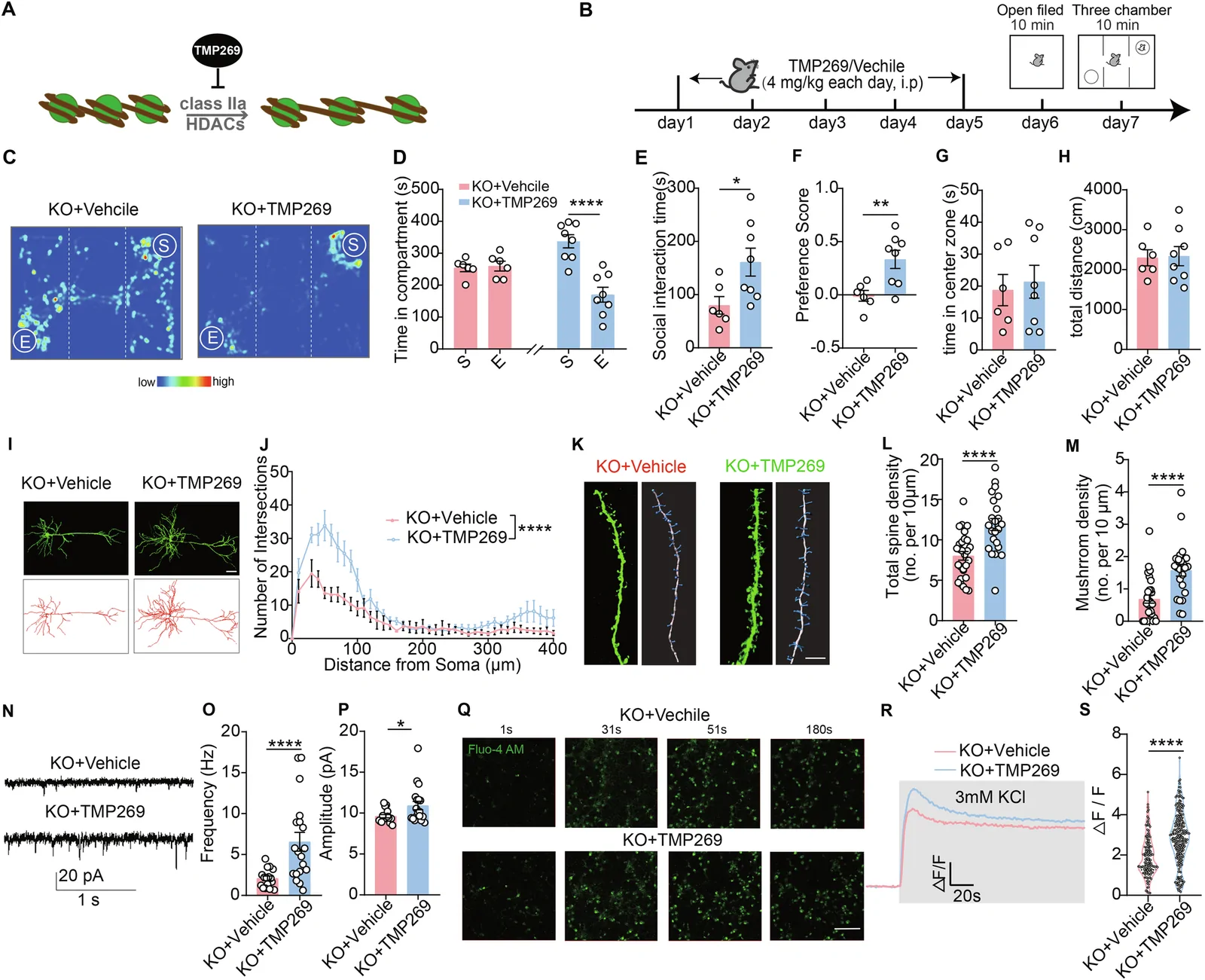
TMP269 administration effectively rescues synaptic and social deficits in *Shank3B* KO mice. (**A**) Schematic diagram of class II HDACs inhibition by TMP269. (**B**) Schematic diagram of the intraperitoneal injection of TMP269 in mice. Representative movement traces of mice (**C**) and quantification of the time spent in the compartment (**D**), social interaction time with the social mouse (**E**), and preference score (**F**) in the three-chamber test of *Shank3B* KO mice treated with TMP269 or Vehicle (S social mouse, E empty cup). *n* = 6 KO+Vehicle group, and *n* = 8 KO + TMP269 group. Data are presented as mean ± SEM. A two-tailed Student’s *t* test was used for statistical analysis. P_KO+TMP269(S) vs. KO+TMP269(E)_ ≤  0.0001, P_KO+Vehcile(Social interaction time) vs. KO+TMP269(Social interaction time)_= 0.0327, P_KO+Vehcile(Preference score) vs. KO+TMP269(Preference score)_= 0.0082. Quantification of the time spent in the center zone (**G**) and total distance traveled (**H**) in the open-field test of *Shank3B* KO mice treated with TMP269 or Vehicle. *n* = 6 KO+Vehicle group, and *n* = 8 KO + TMP269 group. (**I**) Representative pyramidal neurons in the ACC of *Shank3B* KO mice treated with TMP269 or Vehicle. Scale bar, 50 μm. (**J**) Sholl analysis of pyramidal neurons in the ACC. *n* = 6 neurons from 2 KO-vehicle mice and *n* = 6 neurons from 4 KO-TMP269 mice. Data are presented as mean ± SEM. Two‑way ANOVA was used for statistical analysis. *P* ≤  0.0001. (**K**) Representative confocal stacks and three-dimensional reconstruction images of the basal dendrites of ACC pyramidal neurons obtained from *Shank3B* KO mice treated with TMP269 or Vehicle. Scale bar, 5 μm. Summary of total spine density (**L**) and mushroom spine density (**M**) of the basal dendrites of ACC pyramidal neurons. *n* = 28 dendrites from 2 KO+Vehicle mice; *n* = 23 dendrites from 4 KO+TMP269 mice. Data are presented as mean ± SEM. A two‑tailed Student’s *t* test was used for statistical analysis in (**L**). *P* ≤  0.0001. Mann–Whitney *U* test was used for statistical analysis in (**M**). *P* ≤  0.0001. (**N**) Representative mEPSC traces from ACC pyramidal neurons of *Shank3B* KO mice treated with TMP269 or vehicle. Quantification of mEPSC frequency (**O**) and peak amplitudes (**P**). *n* = 16 cells from 2 KO+Vehicle mice, and *n* = 19 cells from 3 KO + TMP269 mice. Data are presented as mean ± SEM. The Mann–Whitney *U* test was used for statistical analysis. P_KO+Vehcile(Frequency) vs. KO+TMP269(Frequency)_= 0.0006, P_KO+Vehcile(Amplitude) vs. KO+TMP269(Amplitude)_= 0.0406. (**Q**–**S**) Calcium response of cultured cortical neurons from *Shank3B* KO mice treated with TMP269 or Vehicle. *n* = 140 cells in the KO+Vehicle group, and *n* = 249 cells in the KO + TMP269 group from three independent experiments. Data are presented as mean ± SEM. The Mann–Whitney *U* test was used for statistical analysis. *P* ≤  0.0001. Scale bar, 100 μm. Source data are available online for this figure.

To further examine whether inhibition of class IIa HDACs could recapitulate the behavioral and synaptic effects of BHB, we systemically administered the selective class IIa HDAC inhibitor TMP269 to *Shank3B* KO mice (Fig. 7A). As illustrated in Fig. 7B, 4 to 6-week-old *Shank3B* KO mice received TMP269 or vehicle at a dose of 4 mg/kg once daily for 5 consecutive days as described previously (Su et al, 2020). TMP269 treatment significantly improved social interaction time and social preference index in *Shank3B* KO mice compared with vehicle-treated controls (Fig. 7C–F). These prosocial effects were selective, as TMP269 administration did not alter anxiety-like behavior (Fig. 7G,H). At the structural level, morphological analysis of ACC pyramidal neurons revealed that TMP269 treatment significantly increased dendritic complexity and dendritic spine density (Fig. 7I–M). Consistent with these structural changes, whole-cell patch-clamp recordings demonstrated marked increases in both the frequency and amplitude of mEPSCs in TMP269-treated mice (Fig. 7N–P). In addition, calcium imaging experiments confirmed enhanced neuronal responsiveness to depolarizing stimulation following TMP269 administration (Fig. 7Q-S). Together, these findings show that pharmacological inhibition of class IIa HDACs is sufficient to restore excitatory synaptic function and social behavior in *Shank3B* KO mice, closely paralleling the effects observed following BHB treatment.

## Discussion

The development of effective, mechanism-based treatments for ASD remains a major challenge in psychiatry. Here, we show that KD alleviates social deficits in a *Shank3B* KO ASD mouse model and identify the ketone body BHB as an important mediator of these effects. Our data further suggest that BHB is associated with normalization of HDAC9 expression in the ACC, together with restoration of excitatory synaptic structure and function in this region. These findings support a metabolite–epigenetic mechanism linking ketogenic metabolism to improvement of social behavior in this ASD-relevant model and suggest that BHB-related pathways may be relevant to ASD-related social dysfunction, particularly in contexts involving synaptic and epigenetic abnormalities.

The KD, a high-fat, low-carbohydrate dietary intervention, has been established as an effective treatment for refractory epilepsy (Lum et al, 2023). Given the high prevalence of epilepsy as a comorbidity in individuals with ASD, KD has been proposed as a potential therapy for ASD. Preliminary clinical observations and preclinical studies, including those using maternal immune activation and BTBR mouse models, have demonstrated improvements in ASD-related behaviors following KD intervention (Olivito et al, 2023; Ruskin et al, 2017; Ruskin et al, 2013). However, the restrictive nature of KD often leads to poor patient adherence, compounded by gastrointestinal issues commonly observed in ASD patients (Schreck and Williams, 2006). To address these challenges, researchers have focused on elucidating its underlying mechanisms. Among the proposed mechanisms, ketone bodies—particularly β-hydroxybutyrate—are considered potential mediators of KD’s therapeutic effects. We demonstrate that KD selectively ameliorates social deficits in *Shank3B* KO mice, a robust ASD model (Peca et al, 2011), while leaving stereotyped grooming and baseline locomotion unchanged. These findings mirror limited clinical observations in children with ASD receiving KD (El-Rashidy et al, 2017) and further support the hypothesis that social and repetitive phenotypes are driven by dissociable neural circuits with distinct metabolic sensitivities.

The identification of BHB as a mediator of the KD effects highlights its potential as a translational therapeutic for ASD. Unlike KD, which requires strict dietary adherence and carries risks of side effects such as gastrointestinal discomfort (Schreck and Williams, 2006), exogenous administration of BHB successfully replicates KD’s prosocial effects without the need for dietary interventions in this model, making it a potentially more practical experimental strategy for further investigation. As an endogenous metabolite, BHB has been reported to exert diverse neuroprotective effects, including HDAC inhibition, GPCR activation, and anti-inflammatory actions (Newman and Verdin, 2017). In the present study, BHB and the class IIa HDAC inhibitor TMP269 produced partially overlapping effects on social and synaptic phenotypes, consistent with the involvement of class IIa HDAC-related mechanisms. However, TMP269 does not provide HDAC9-specific pharmacological evidence, and the effects of BHB are unlikely to be explained exclusively by HDAC9 regulation. Therefore, although our findings support BHB as a promising mechanistic and translational direction, further studies will be required to define its pharmacodynamics, dose–response relationships, long-term safety, and efficacy across additional ASD models and human-relevant systems before broader therapeutic conclusions can be drawn.

At the molecular level, our results identify HDAC9 as a critical epigenetic effector that mediates ACC-specific social dysfunction but not anxiety-like behaviors in *Shank3B* KO mice. It suggests that the upregulation of HDAC9 in the ACC reflects a dedicated pathway underlying social impairment, whereas the broader anxiolytic effects of BHB likely involve additional, distinct molecular pathways, possibly through its known anti-inflammatory or mitochondrial function-enhancing properties (Ortí et al, 2023). Our overexpression experiments indicate that HDAC9 upregulation is sufficient to induce synaptic and behavioral phenotypes in this model. BHB may counteract pathological HDAC9 upregulation through direct inhibition of its deacetylase activity or modulation of upstream regulators. Emerging evidence also links environmental stress to HDAC9 upregulation, suggesting that metabolic interventions, such as KD or BHB administration, might mitigate such epigenetic changes. This supports the interpretation that BHB acts to reverse these phenotypes, at least in part, by suppressing HDAC9 expression.

Recent research shows that HDAC9 forms context-specific multiprotein complexes, likely explaining its selective impact on social behavior and the regional specificity of its effects in the ACC compared to other brain areas. Indeed, recent studies have established the ACC as a key node for processing social information, with its dysfunction being linked to social deficits in autism (Kitagawa et al, 2024; Qi et al, 2025; Willbrand et al, 2026). Although we did not perform an exhaustive brain-wide survey, we examined several major regions implicated in social behavior, including the hippocampus, striatum, and ACC (Sato et al, 2023). Within this set of regions, HDAC9-related changes were most evident in the ACC, supporting a relatively selective involvement of this region in the present model, while not excluding contributions from other brain areas. In addition to pyramidal neuron dysfunction, the cellular heterogeneity of the ACC, including distinct interneuron subtypes such as parvalbumin and somatostatin interneurons, has been shown to shape social behavior (Huang et al, 2024; Qi et al, 2025). These findings highlight the complexity of ACC circuit regulation and suggest that multiple cell populations may contribute to ASD-related social dysfunction. Although our current data primarily implicate ACC excitatory neurons and overall ACC tissue-level HDAC9 dysregulation, they do not exclude possible contributions from interneuron populations. Together, these findings position the BHB-HDAC9 pathway as a critical metabolic‒epigenetic interface that links cellular energy states to the regulation of synaptic function and social behavior in ASD.

A key unresolved question is how SHANK3 deficiency leads to selective HDAC9 upregulation in the ACC. SHANK3 loss disrupts excitatory synaptic transmission and impairs LTCC/CaMKII-dependent excitation–transcription coupling (Perfitt et al, 2020), providing a plausible upstream mechanism for altered activity-dependent transcriptional regulation. In neurons, Ca²⁺/CaMK signaling regulates the phosphorylation-dependent nucleocytoplasmic trafficking of class IIa HDACs (McKinsey et al, 2000), and HDAC9 itself undergoes neuronal activity-dependent nuclear export from the nucleus to the cytoplasm (Sugo et al, 2010). Given that HDAC9 is preferentially expressed in post-mitotic neurons and has been implicated in neuropsychiatric disease (Lang et al, 2012), impaired Ca²⁺-dependent signaling in *Shank3B*-deficient neurons may favor aberrant HDAC9 accumulation and transcriptional repression. Consistent with this model, pharmacological enhancement of Ca²⁺ influx with the L-type Ca²⁺ channel agonist Bay K8644 reduced the elevated HDAC9 immunofluorescence intensity and Hdac9 mRNA expression in *Shank3B*-deficient cultured neurons, with relatively limited effects in WT neurons. These findings support a potential link between altered Ca²⁺ signaling and HDAC9 dysregulation in the *Shank3B*-deficient condition. However, because these experiments were performed in cultured cortical neurons and did not directly examine HDAC9 phosphorylation, nucleocytoplasmic localization, or ACC-specific regulation in vivo, this pathway should be regarded as a plausible mechanistic model rather than definitive proof. Consistent with these findings, we observed reduced social interaction-induced c-Fos expression in the KO ACC, which was increased by BHB treatment. This result is consistent with improved recruitment of ACC neurons during social behavior, although c-Fos remains an indirect marker of neuronal activity rather than a direct measure of complete circuit reinstatement. BHB likely restores excitatory drive and activity-dependent gene programs such as *Foxo3a* and *Bdnf* (Hu et al, 2020; Shimazu et al, 2013) by directly inhibiting HDAC activity and indirectly promoting a more permissive chromatin landscape, thereby inducing synaptogenesis and AMPAR expression. Additional pathways linked to the Wnt/β-catenin system, documented in other *Shank3* models, may also converge on HDAC regulation (Qin et al, 2018; Wang et al, 2023). These multilayered mechanisms may elucidate the variable epigenetic signatures across *Shank3* models: *Shank3*^*+/ΔC*^ mice exhibit HDAC2-mediated hypoacetylation in the prefrontal cortex (Ma et al, 2018; Qin et al, 2018), while our *Shank3B*^*−/−*(Δ13–16)^ mice (Peca et al, 2011) show ACC-specific HDAC9 upregulation. This divergence likely reflects differences in genetic targeting, circuit vulnerability, and cell-type-specific epigenetic regulation, suggesting that ASD-associated epigenetic pathology is similarly heterogeneous. Addressing this heterogeneity will require mechanism-guided, circuit-targeted therapeutic strategies.

Despite these findings, several limitations of the study should be acknowledged. First, although BHB improved social behavior and ameliorated associated molecular and cellular abnormalities, its efficacy on other core ASD symptoms, such as repetitive behaviors and sensory processing deficits, was limited and not thoroughly evaluated. Second, our study focused solely on the *Shank3B* KO genetic model, which, although reflective of key ASD synaptic phenotypes, requires validation across other genetic and environmental ASD models, as well as in human neuronal systems, such as iPSC-derived neurons or organoids, to confirm generalizability. Third, while our data strongly implicate HDAC9, the precise downstream transcriptional programs and chromatin loci affected by HDAC9 remain to be defined. Fourth, BHB may have multiple parallel effects, such as epigenetic, metabolic, and anti-inflammatory effects, and disentangling which of these are causal for synaptic rescue will require temporally resolved and cell-type-specific manipulations.

In summary, our study reveals a metabolic–epigenetic axis through which BHB, the principal effector of a KD, suppresses HDAC9 overexpression in ACC neurons to restore excitatory synaptic integrity and social behavior in *Shank3B* KO mice. These findings support a role for HDAC9-related mechanisms in ASD-related social dysfunction in this model and provide a framework for further investigation of BHB-based or HDAC9-related interventions in additional preclinical models and human-relevant systems.

## Methods

**Table:** Reagents and tools table

Reagent/resource	Reference or source	Identifier or catalog number
**Experimental models**		
Mouse: C57BL/6 J mice	The Fourth Military Medical University	N/A
Mouse: *Shank3B*^*−/−*^ mice	The Jackson Laboratory	N/A
**Antibodies**		
Rabbit anti-GFP	Invitrogen	A11122
Mouse anti-GRIA1	Proteintech	67642
Rabbit anti-Glutamate receptor 2	Proteintech	11994
Rabbit anti-SHANK3	Cell Signaling Technologies	64555
Rabbit anti-ACTIN	Proteintech	66009
Mouse anti-HDAC5	Santa Cruz	sc-133225
Rabbit anti-HDAC8	Affinity	AF6481
Rabbit anti-HDAC9	Abcam	AB109446
Mouse anti-HDAC9	Santa Cruz	sc-398003
Mouse anti-CaMKII (phospho T286)	Abcam	AB171095
Mouse anti-MAP2A	Millipore	MAB364
Rabbit anti-cFOS	Cell Signaling Technologies	2250
HRP-conjugated-Goat-Anti-Rabbit-IgG (H + L)	Proteintech	SA00001-2
HRP-conjugated-Goat-Anti-Mouse-IgG (H + L)	Proteintech	SA00001-1
488 Alexa Fluor anti-mouse IgG	Invitrogen	A11001
488 Alexa Fluor anti-rabbit IgG	Invitrogen	A21206
594 Alexa Fluor anti-mouse IgG	Invitrogen	A21203
594 Alexa Fluor anti-rabbit IgG	Invitrogen	A21207
DAPI	Sigma-Aldrich	D9564
**Bacterial and viral strains**		
AAV2/9-hSyn-EGFP-P2A-Hdac9-3XFLAG-WPRE	OBiO Technology	N/A
AAV2/9-hSyn-EGFP-P2A-3XFLAG-WPRE	OBiO Technology	N/A
AAV9-hSyn-EGFP-P2A-EGFPf	PackGene	N/A
**Oligonucleotides and other sequence-based reagents**		
Gapdh forward primer	AGGTCGGTGTGAACGGATTTG	N/A
Gapdh reverse primer	TGTAGACCATGTAGTTGAGGTCA	N/A
Hdac1 forward primer	AGTCTGTTACTACTACGACGGG	N/A
Hdac1 reverse primer	TGAGCAGCAAATTGTGAGTCAT	N/A
Hdac2 forward primer	GGAGGAGGCTACACAATCCG	N/A
Hdac2 reverse primer	TCTGGAGTGTTCTGGTTTGTCA	N/A
Hdac3 forward primer	GCCAAGACCGTGGCGTATT	N/A
Hdac3 reverse primer	GTCCAGCTCCATAGTGGAAGT	N/A
Hdac4 forward primer	CACTGCATTTCCAGCGATCC	N/A
Hdac4 reverse primer	AAGACGGGGTGGTTGTAGGA	N/A
Hdac5 forward primer	AGCACCGAGGTAAAGCTGAG	N/A
Hdac5 reverse primer	GCTGTGGGAGGGAATGGTT	N/A
Hdac7 forward primer	GAACTCTTGAGCCCTTGGACA	N/A
Hdac7 reverse primer	GGTGTGCTGCTACTACTGGG	N/A
Hdac8 forward primer	ATCTCCAGAAGGTCAGCCAAG	N/A
Hdac8 reverse primer	ACATTCCGTCAATCAGGCATT	N/A
Hdac9 forward primer	GCGGTCCAGGTTAAAACAGAA	N/A
Hdac9 reverse primer	GCCACCTCAAACACTCGCTT	N/A
**Chemicals, enzymes, and other reagents**		
TMP269	MedChemExpress,	HY-18360
(R)-(-)-Sodium β-hydroxybutyrate	Sigma-Aldrich	298360
Tetrodotoxin	Tocris	1078/1
AP5	Tocris	0106/1
Bay K8644	TargetMol	T6777
**Critical commercial assays**		
BCA Protein Assay Kit	Thermo Fisher Scientific	23235
RIPA buffer	BiYunTian	P0013B
phosphatase inhibitors	Roche	4693132001
TRIzol	Thermo Fisher Scientific	15596018CN
PrimeScript™ RT Master Mix	Takara	RR036A
TB Green qPCR Premix	Takara	639676
Bovine serum albumin	Sigma	V900933
Goat serum	Thermo Fisher Scientific	16210064
Triton X-100	Sigma	85112
PBS	Sigma	10010023
**Software**		
SMART Video Tracking System	Panlab S.L.U., Spain	V3.0
GraphPad Prism	GraphPad Software, Boston, USA	RRID: SCR_002798
Fiji software	National Institutes of Health, USA	RRID: SCR_003070
pClamp	Molecular Devices, USA	RRID: SCR_11323
Imaris software	Bitplane, UK	RRID: SCR_007370
**Other**		
Ketogenic Diet	Xietong Organism Company, China	XTKD01
Standard Control Diet	Xietong Organism Company, China	XTKDCON

### Mice

Wild-type (WT) C57BL/6 mice were obtained from the animal facility of the Fourth Military Medical University. *Shank3B* KO mice were on a pure C57BL6 background (Peca et al, 2011). All the mice were housed in standard facilities under controlled conditions, with a controlled temperature (23–25 °C), a 12/12-h light/dark cycle, and ad libitum access to food and water. Both male and female mice were used and were distributed as evenly as possible across experimental groups. Mice were age- and weight-matched within each experiment. Male and female mice were pooled for analysis, and sex was not included as an independent factor in the statistical analysis, as the experiments were not treated as an independent variable in the statistical analysis. All procedures were approved by the Institutional Animal Care and Use Committee of the Fourth Military Medical University (IACUC20260015) in compliance with the Principles of Medical Laboratory Animal Care issued by the Ministry of Health of China.

### Virus injection

An adeno-associated virus (AAV) expressing HDAC9 (AAV‑hSyn‑EGFP‑P2A‑Hdac9‑3XFLAG‑WPRE, titer: 4.68 × 10^12^ GC/ml) was packaged by OBiO Technology (Shanghai, China). The mice underwent stereotactic brain injections, and isoflurane was used for general anesthesia. The virus was injected into the bilateral ACC according to the following stereotaxic coordinates: antero-posterior: +0.8 mm, medio-lateral: ±0.3 mm, and dorsoventral: −1.75 mm—relative to the bregma per a stereotactic atlas. Both the HDAC9‑expressing AAV and the control AAV (AAV-hSyn-EGFP-P2A-3XFLAG-WPRE, titer: 1.5 × 10^1^^3^ GC/ml) were diluted to 1 × 10^12^ GC/ml, and 200 nL of the diluted virus was bilaterally injected into the ACC per side. AAV9-hSyn-EGFP-P2A-EGFPf which is used to sparsely label neurons was developed in our own laboratory (Chen et al, 2020), and was administered to mice via a single 50 μl retroorbital injection into the venous sinus at a titer of 5 × 10^11^ GC/ml.

### Ketogenic diet administration

At weaning, the KO mouse littermates were housed in same-sex cages. KO mice were divided into two groups: the KD group which was given a ketogenic diet (a diet characterized by 90% energy derived from fat and low carbohydrate levels, with cocoa butter as its primary component; XTKD01, Xietong Organism Company, China), and the SD group which was given a standard diet (a diet formulated to provide 10% energy from fat, serving as the standard control for KD, XTKDCON, Xietong Organism Company, China.). The starting weights of all groups showed no significant differences within each sex. The mice had ad libitum access to the chow, and chow was supplied daily. Behavioral assessments were initiated following a 4-week ketogenic diet.

### TMP269 treatment

TMP269 powder was dissolved in dimethyl sulfoxide and diluted with corn oil according to the manufacturer. The drug was administered to *Shank3B* KO mice via i.p injection at a dose of 4 mg/kg for 5 consecutive days.

### Sodium β-hydroxybutyrate treatment

Sodium β-hydroxybutyrate salt was dissolved in 0.01 M phosphate buffer solution and was administered by gavage at a dose of 120 mg/kg for 7 consecutive days.

### Behavioral measurements

#### Three-chamber test

The three-chamber sociability test was conducted using a black apparatus consisting of three connected chambers, each measuring 30 cm × 40 cm × 20 cm. The central compartment featured two sliding doors that allowed access to the adjacent chambers. Two cylindrical grid cages with a cone roof were placed in the corners of the outer chambers, enabling visual, olfactory, and auditory interactions between the test mouse and the caged mouse. First, the test mouse was placed in the central chamber and allowed to habituate for 5 min. Subsequently, an empty cage and a stimulus mouse were positioned in the cylindrical cages, and the test mouse was allowed access to all three chambers for a 10-min recorded trial. After each test, the apparatus was cleaned with 75% alcohol. The video recordings of these trials were analyzed using SMART 3.0 software to measure the time spent in each chamber and the time spent near the cages. Sociability was quantified by calculating the preference score using the following formula: (time spent in the social zone - time spent in the empty zone) / (total time spent in the social zone and empty zone).

#### Open-field test

The mouse was placed in the central area of a black box (50 × 50 × 50 cm) to freely explore the area and was video recorded for 10 min. The video recordings of these trials were analyzed using SMART 3.0 software. The time spent in the center zone and the total distance traveled were measured.

#### Self-grooming behavior

Each mouse was housed individually in a transparent plastic chamber (20 × 20 × 25 cm) for a 30 min free activity session, during which it was recorded in a side view. After each session, the chambers were cleaned with 75% ethanol. Grooming behavior was scored manually by observers who were blinded to the animal groups.

### Electrophysiology recording

To prepare the brain slices, the test mice were anesthetized with pentobarbital sodium (30 mg/kg body weight), and cardiac perfusion was performed with carbogenated ice-cold cutting solution, which consisted of 115 mM choline chloride, 2.5 mM KCl, 1.25 mM NaH_2_PO_4_, 0.5 mM CaCl_2_, 8 mM MgCl_2_, 26 mM NaHCO_3_, 10 mM D-(+)-glucose, 0.1 mM L-ascorbic acid, and 0.4 mM sodium pyruvate. Coronal slices (300 µm) encompassing the ACC were obtained using a vibrating microtome (7000SMZ; Campden, England) and maintained in the same cutting solution. The sections underwent a 30-min recovery period in carbogenated cutting solution at 32 °C, followed by equilibration in carbogenated artificial cerebrospinal fluid (ACSF: 119 mM NaCl, 2.3 mM KCl, 1.0 mM NaH_2_PO_4_, 26 mM NaHCO_3_, 11 mM D-glucose, 1.3 mM MgCl_2_, and 2.5 mM CaCl_2_; pH 7.4, 295–300 mOsm/L) at room temperature for 60 min. Electrophysiological recordings were obtained using a Multiclamp 700B amplifier (Molecular Devices, USA). Neuronal activity was monitored at a holding potential of -65 mV with 1 µM tetrodotoxin and 2H-1-benzopyran-7-propanoic acid, and β-cyclopropyl-2-[2-fluoro-4-(2-methoxy-4-pyridinyl)phenyl]-3,4-dihydro-α-methyl-(αS, βR,2S) (AP5) present in the bath solution to isolate miniature excitatory postsynaptic currents (mEPSCs). Data were acquired and analyzed using Clampex 10.7.

### Primary cortical neuron culture and pharmacological treatments

Primary cortical neurons were isolated and cultured as previously described (Qi et al, 2022). Briefly, cortical tissues were dissected from embryonic days 16 to 18 C57BL/6J or *Shank3B* KO mouse fetuses and mechanically dissociated in ice-cold HBSS. The dissociated cells were collected by centrifugation and plated on poly-L-lysine-coated dishes in Neurobasal medium supplemented with B27, glutamine, penicillin, and streptomycin.

For morphological analysis, primary cortical neurons were subjected to lentiviral-mediated Hdac9 overexpression or treated with BHB (2 mM) at DIV3. At DIV6, neurons were fixed and processed for immunofluorescence staining with MAP2 and DAPI to visualize neuronal morphology and nuclei, respectively. Confocal images were acquired using a laser scanning confocal microscope, and neuronal morphology was analyzed based on MAP2-positive signals.

For Bay K8644 stimulation experiments, primary cortical neurons were treated with Bay K8644 (2 μM) or an equivalent volume of DMSO as the vehicle control at DIV6. For mRNA analysis, cells were collected 6 h after treatment, followed by total RNA extraction, reverse transcription, and quantitative real-time PCR analysis of Hdac9 expression. For immunofluorescence analysis, neurons were fixed 12 h after Bay K8644 treatment and processed for HDAC9 immunostaining. Images were acquired under identical confocal imaging settings, and the mean fluorescence intensity of HDAC9 in neurons was quantified.

### In vitro calcium imaging

Primary cortical Neurons were loaded with aCSF containing 3 μM Fluo-4AM for 15 min at 37 °C at DIV6. Following loading, the neurons were washed three times with aCSF to remove excess dye. Fluorescence imaging was performed using a confocal microscope (Olympus FV3000, Japan). Fluo-4am was excited with a 488 nm laser, and images were acquired at 1-s intervals for a total duration of 180 s. To induce neuronal activity, 3 mM KCl was added to the aCSF 30 s after the initiation of imaging. The normalized changes in Fluo-4am fluorescence intensity within cell bodies were quantified as ΔF/F = (F − F0)/F0, where F0 represents the baseline fluorescence intensity before KCl application. For statistical analysis, the peak ΔF/F response after KCl stimulation was used. The same imaging settings, cell-selection criteria, and analysis procedures were applied across all experimental groups. Data were collected from at least three independent experiments for statistical analysis.

### Western blotting

ACC tissues were homogenized and lysed in RIPA buffer supplemented with protease and phosphatase inhibitors. The protein concentration of each sample was determined using a BCA Protein Assay Kit following the manufacturer’s instructions. The proteins were then resolved by SDS‒PAGE as previously described (Hu et al, 2020). Following electrophoresis, the proteins were transferred onto PVDF membranes, which were subsequently incubated with primary antibodies. After being washed, the membrane was probed with horseradish peroxidase-conjugated secondary antibodies. Protein bands were visualized using a chemiluminescence imaging system (Jena, Germany), and band intensities were quantified using ImageJ software. The antibodies used are listed in the Key Resources Table.

### RNA extraction and quantitative real-time PCR

Quantitative real-time PCR was performed as described (Hu et al, 2020). After behavioral testing, animals were anesthetized with isoflurane, and brains were rapidly collected. The anterior cingulate cortex (ACC) was microdissected from freshly prepared coronal sections under stereomicroscopic guidance. Total RNA was purified using TRIzol reagent according to the manufacturer’s instructions. Reverse transcription was performed with PrimeScript™ RT Master Mix (Takara) using 1000 ng of total RNA as input to generate cDNA. Quantitative PCR reactions were set up with 2× Universal SYBR Green Fast qPCR Mix (Takara) and run on a StepOnePlus™ Real-Time PCR System (Thermo). Primer sequences are listed in the Reagents and tools table.

### Immunofluorescence

The mice were deeply anesthetized with isoflurane and transcardially perfused with 0.01 M PBS, followed by 4% (w/v) paraformaldehyde. The brains were post fixed in the same fixative overnight at 4 °C and subsequently cryoprotected in 30% (w/v) sucrose solution. Coronal brain sections containing the ACC were cut at a thickness of 30 μm using a freezing microtome. The sections were incubated with primary antibodies overnight at room temperature, followed by incubation with Alexa Fluor-conjugated secondary IgG antibodies for 4 h. Finally, the sections were stained with DAPI for nuclear labeling, air-dried, and coverslipped using 50% (v/v) glycerol. Imaging was performed using an FV3000 confocal microscope (Olympus, Japan).

For mice injected with sparse-labeled virus, the procedure was performed as described previously (Ge et al, 2024). In brief, the mice were perfused with 0.01 M PBS and 4% (w/v) PFA, and the brains were post fixed in PFA overnight. The following day, the brains were sectioned at a thickness of 200 µm using a vibratome. Brain slices were washed three times with 0.01 M PBS, permeabilized with 0.3% Triton X-100 for 2 h at room temperature, and subsequently blocked for 1 h at room temperature in blocking solution containing 5% bovine serum albumin (BSA), 15% goat serum, and 0.3% Triton X-100 in 0.01 M PBS. The slices were then incubated with primary antibody (anti-GFP polyclonal antibody; 1:1000; A11122; Invitrogen) diluted in blocking solution for 48 h at 4 °C. After being washed three times with blocking solution, the slices were incubated with a secondary antibody (Alexa Fluor 488-conjugated donkey anti-rabbit IgG, 1:500) for 12 h at room temperature. Following three washes with PBS containing 0.2% Tween 20, the slices were mounted with 50% (v/v) glycerol. Images of labeled neurons and dendritic spines were acquired using an Olympus FV3000 confocal microscope. Three-dimensional (3D) reconstruction and quantitative analysis were performed using Imaris software (v7.7.1, serial number: 32mr-rfhf-7hbu-jb58, Bitplane).

### LC/MS measurement

To measure key metabolites in the serum samples, a liquid chromatography–mass spectrometry (LC–MS) system (Shimadzu) was used to achieve chromatographic separation using a Kinetex F5 column (3 × 100 mm, 2.6 μm). The isocratic mobile phase, consisting of 95% acetonitrile, 10 mM ammonium acetate, and 0.04% ammonia, was maintained at a flow rate of 200 µL/min.

### Statistical analysis

All the statistical analyses were performed in GraphPad Prism software (version 8.0; GraphPad, CA, USA). Electrophysiological data analysis and dendritic spine quantification were performed by investigators blinded to the experimental groups. No formal blinding was applied to the other experiments. Data are presented as the mean ± standard error of the mean (SEM), unless otherwise indicated. Statistical significance was assessed using two-sided tests where applicable. For comparisons between two groups, an unpaired two-tailed Student’s *t* test or Mann–Whitney *U* test was used, as appropriate. For comparisons among multiple groups, one-way ANOVA was performed, followed by Dunnett’s post hoc test when each experimental group was compared with a single control group, or Tukey’s multiple comparisons test when all groups were compared with one another. For datasets involving two independent variables, such as genotype/treatment effects across distance or time, two-way ANOVA was used, followed by the indicated multiple-comparison test, including Bonferroni’s multiple comparisons test where appropriate. For datasets that did not meet the assumptions for parametric testing, the Kruskal–Wallis test was used, followed by Dunn’s multiple comparisons test. A *P* value < 0.05 was considered statistically significant. For datasets involving multiple cells, dendrites, or neuronal structures, the unit of analysis and the number of analyzed cells/dendrites, as well as the contributing animals, are indicated in the corresponding figure legends and in Table EV2. Detailed information on the statistical tests used for each analysis, including the corresponding figure panels, comparison groups, sample sizes, and multiple-comparison procedures, is provided in Table EV2.

## Supplementary information

Table EV1

Table EV2

Peer Review File

Source data Fig. 1

Source data Fig. 2

Source data Fig. 3

Source data Fig. 4

Source data Fig. 5

Source data Fig. 6

Source data Fig. 7

Figure EV1 Source Data

Figure EV2 Source Data

Figure EV3 Source Data

Figure EV4 Source Data

Expanded View Figures

## Data Availability

The metabolomics data generated in this study have been deposited in the MetaboLights database under accession number MTBLS14381 and are publicly available at https://www.ebi.ac.uk/metabolights/MTBLS14381. The source data of this paper are collected in the following database record: biostudies:S-SCDT-10_1038-S44321-026-00491-9.
