# Supplementary material for: β-hydroxybutyrate restores social deficits by suppressing HDAC9 in the ACC of Shank3B-deficient mice
Source: EMBO Mol Med. 2026 Jul 21;18(9):3517–39. doi: 10.1038/s44321-026-00491-9 (PMC13562639; doi:10.1038/s44321-026-00491-9)
Supplement: Supplementary file 1 [file 44321_2026_491_MOESM15_ESM.pdf]

# Expanded View Figures

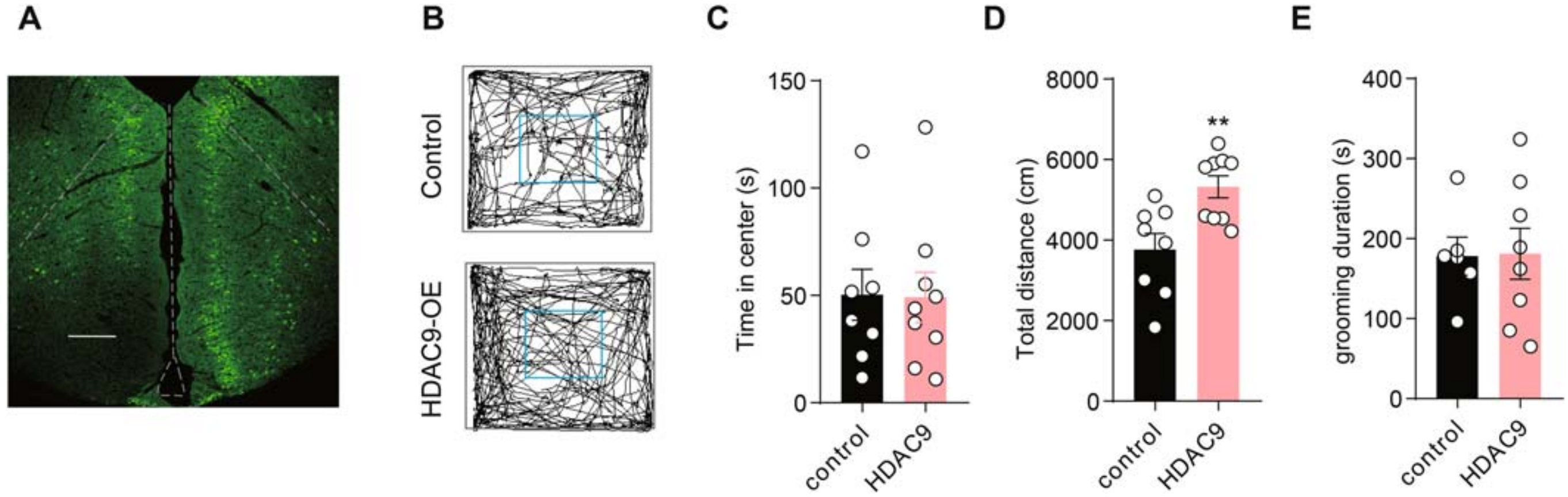


**Figure EV1. HDAC9 overexpression in ACC neurons does not affect anxiety-like or grooming behaviors.**

(**A**) Representative images showing viral expression and injection site localization in the ACC following stereotaxic injection of HDAC9-overexpressing AAV. Scale bar, 200 µm. (**B**) Representative movement traces of mice in the open-field test at 4 weeks after injection of control virus or HDAC9-overexpression virus into the ACC. Quantification of center zone dwell time (**C**) and total distance traveled (**D**) in the open-field test of mice injected with control virus or HDAC9-overexpression virus. n = 8 mice in the control group and 9 mice in the HDAC9-OE group. Data are presented as mean ± SEM. A two-tailed Student's *t* test was used for statistical analysis. $P = 0.0052$. (**E**) Quantification of grooming duration in mice at 4 weeks after injection of control virus or HDAC9-overexpression virus. $n = 6$ mice in the control group and 8 mice in the HDAC9-OE group. Data are presented as mean ± SEM. Source data are available online for this figure.

© The Author(s) 

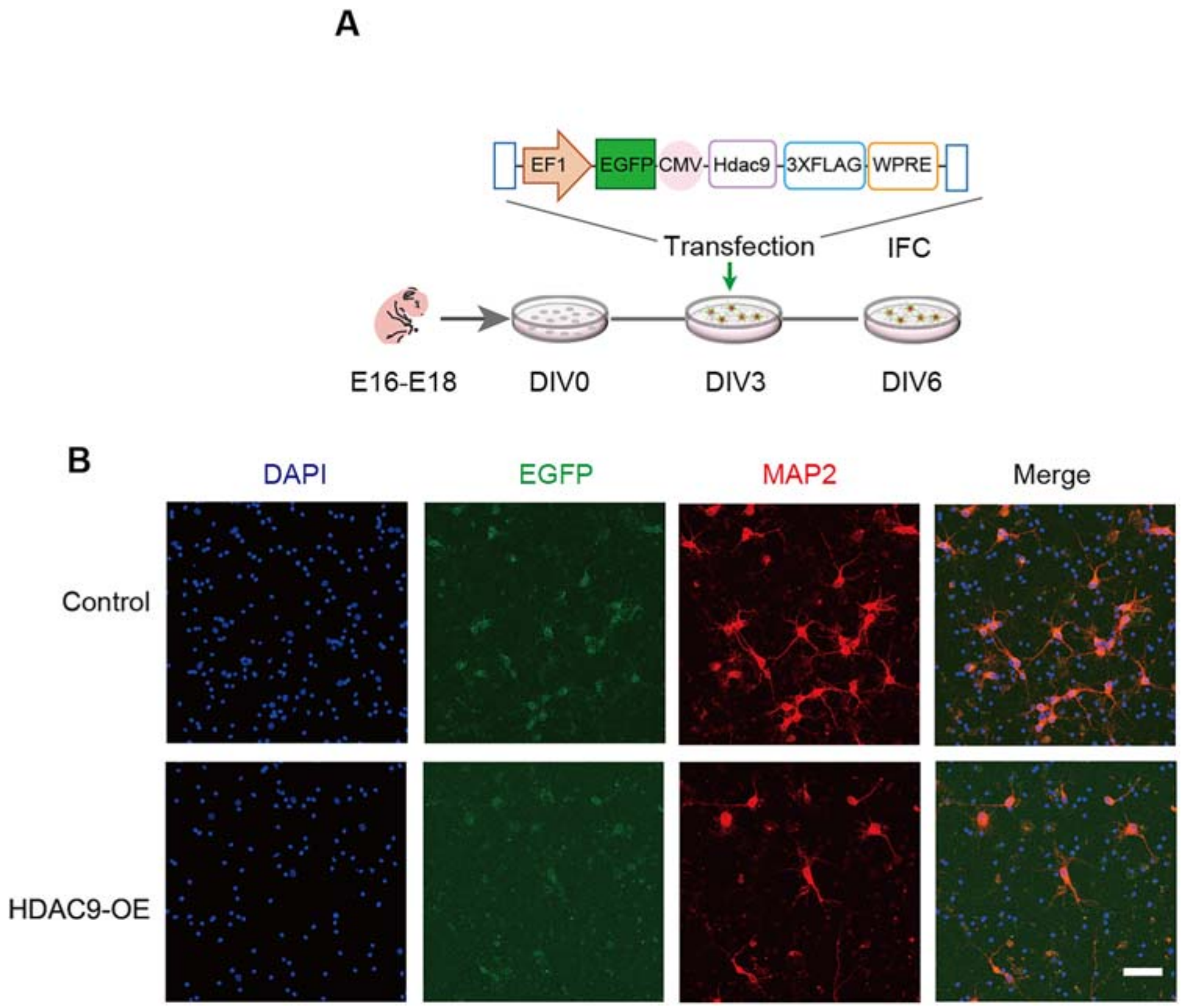


**Figure EV2. HDAC9 overexpression impairs the growth of cultured cortical neurons.**

(**A**) Timeline of the transfection of cultured cortical neurons with control lentivirus or HDAC9-overexpressing lentivirus. (**B**) Representative immunofluorescence images of cultured cortical neurons transfected with control lentivirus or HDAC9-overexpressing lentivirus. Scale bar, 50 µm. Source data are available online for this figure.

 © The Author(s)

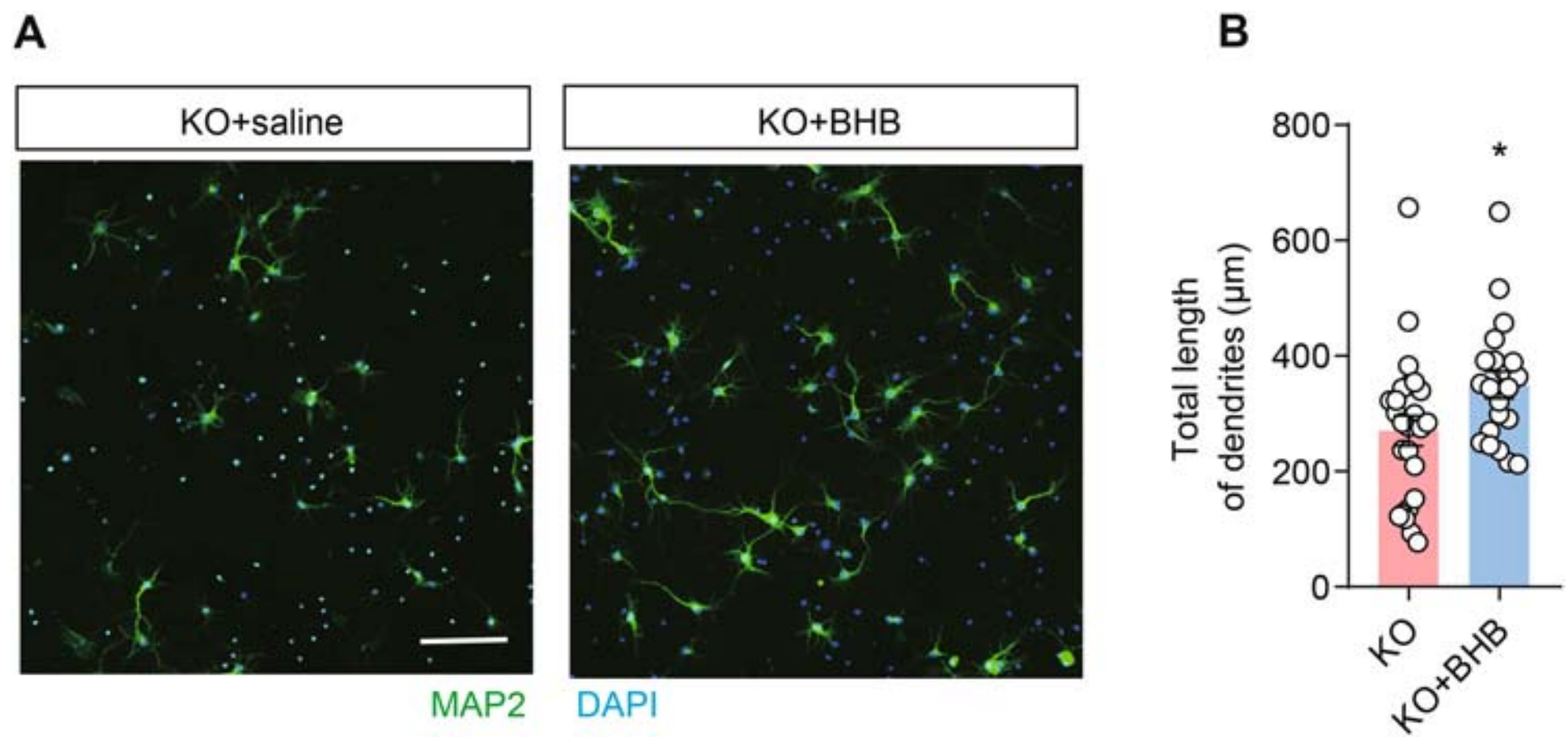


**Figure EV3. Sodium BHB administration increases dendritic length in *Shank3B* KO cortical neurons.**

(**A**) Representative immunofluorescence images showing DAPI and MAP2 staining in cultured cortical neurons from *Shank3B* KO mice treated with saline or 2 mM sodium BHB. (**B**) Quantification of total dendritic length in *Shank3B* KO cortical neurons treated with saline or 2 mM sodium BHB. $n = 25$ cells in the KO+Saline group, and $n = 21$ cells in the KO + BHB group. Data are presented as mean ± SEM. A two-tailed Student's *t* test was used for statistical analysis. $P = 0.0298$. Source data are available online for this figure.

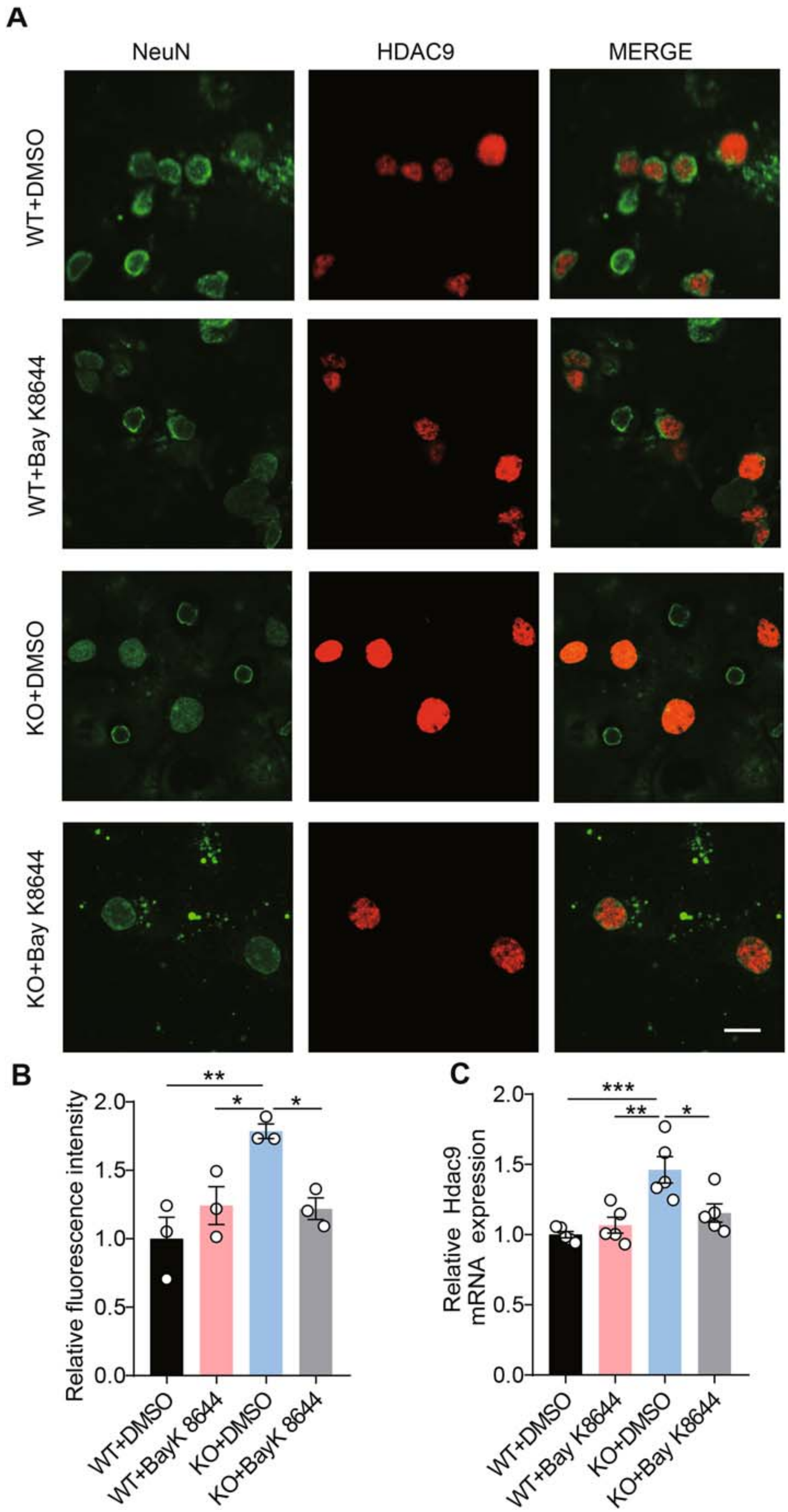
A
NeuN
HDAC9
MERGE
WT+DMSO
WT+Bay K8644
KO+DMSO
KO+Bay K8644
B
Relative fluorescence intensity
2.0
1.5
1.0
0.5
0.0
**
*
*
WT+DMSO
WT+BayK 8644
KO+DMSO
KO+BayK 8644
C
Relative Hdac9 mRNA expression
2.0
1.5
1.0
0.5
0.0
***
**
*
WT+DMSO
WT+Bay K8644
KO+DMSO
KO+Bay K8644

 © The Author(s)

**Figure EV4. Bay K8644 treatment reduces HDAC9 expression in *Shank3B* KO cultured cortical neurons.**

(**A**) Representative immunofluorescence images showing NeuN and HDAC9 staining in cultured cortical neurons from WT and *Shank3B* KO mice treated with vehicle (DMSO) or the L-type calcium channel agonist Bay K8644 (2 μM) for 12 h. Scale bar = 10 μm. (**B**) Quantification of the relative fluorescence intensity of HDAC9 per cell from each group. *n* = 3 slices with 36 neurons in the WT + DMSO group, *n* = 3 slices with 49 neurons in the WT+ Bay K8644 group, *n* = 3 slices with 33 neurons in the KO + DMSO group, and *n* = 3 slices with 19 neurons in the KO+ Bay K8644 group. Data are presented as mean ± SEM. One-way ANOVA followed by Bonferroni's multiple comparisons test was used for statistical analysis. $P_{WT+DMSO\ vs.\ KO+DMSO} = 0.0021$, $P_{KO+DMSO\ vs.\ WT+Bay\ K8644} = 0.0163$, $P_{KO+DMSO\ vs.\ KO+Bay\ K8644} = 0.0133$. (**C**) Relative mRNA expression of HDAC9 measured by quantitative real-time PCR in cultured cortical neurons under the same treatment conditions. *n* = 5 independent biological samples each group. Data are presented as mean ± SEM. One-way ANOVA followed by Tukey's multiple comparisons test was used for statistical analysis. $P_{WT+DMSO\ vs.\ KO+DMSO} = 0.0006$, $P_{KO+DMSO\ vs.\ WT+Bay\ K8644} = 0.0028$, $P_{KO+DMSO\ vs.\ KO+Bay\ K8644} = 0.0190$. Source data are available online for this figure.

© The Author(s)
