# Supplementary material for: β-hydroxybutyrate restores social deficits by suppressing HDAC9 in the ACC of Shank3B-deficient mice
Source: EMBO Mol Med. 2026 Jul 21;18(9):3517–39. doi: 10.1038/s44321-026-00491-9 (PMC13562639; doi:10.1038/s44321-026-00491-9)
Supplement: Supplementary file 2 [file 44321_2026_491_MOESM3_ESM.pdf]

EMBO Molecular Medicine

β-hydroxybutyrate Restores Social Deficits by Suppressing HDAC9 in the ACC of Shank3B-deficient Mice

Erling Hu, Jialong Li, Chuchu Qi, Junye Ge, Yuanmeng Li, Qian Xue, Shengxi Wu, and Wenting Wang

Corresponding authors: Wenting Wang (wwt0657@fmmu.edu.cn) , Shengxi Wu (shengxi@fmmu.edu.cn)

Review Timeline:

Submission Date:	27th Feb 26
Editorial Decision:	15th Apr 26
Revision Received:	15th Jun 26
Editorial Decision:	1st Jul 26
Revision Received:	3rd Jul 26
Accepted:	8th Jul 26

Editor: Jingyi Hou

Transaction Report:

(Note: With the exception of the correction of typographical or spelling errors that could be a source of ambiguity, letters and reports are not edited. Depending on transfer agreements, referee reports obtained elsewhere may or may not be included in this compilation. Referee reports are anonymous unless the Referee chooses to sign their reports.)

15th Apr 2026

Dear Dr. Wang,

Thank you again for submitting your work to EMBO Molecular Medicine. First, I would like to apologise for the delay in the review process. We have now received evaluations from two of the three referees who had agreed to assess your manuscript. Despite several reminders, we were unable to obtain a report from Referee #3. In the interest of time and given that the recommendations from the two received reviews are aligned, I have decided to proceed with a decision rather than delay the process further.

The referees' recommendations are rather clear, so I won't repeat the points listed below. It is important to carefully address all the issues raised by the referees. Please feel free to contact me in case you would like to discuss in further detail any of the issues raised by the referees.

As you may already know, our editorial policy allows in principle a single round of major revision, and it is therefore essential to provide responses to the referees' comments that are as complete as possible.

EMBO Molecular Medicine has a "scooping protection" policy, whereby similar findings that are published by others during review or revision are not a criterion for rejection. Should you decide to submit a revised version, I do ask that you get in touch after three months if you have not completed it, to update us on the status.

Please also contact us as soon as possible if similar work is published elsewhere. If other work is published, we may not be able to extend the revision period beyond three months.

Please read below for important editorial formatting and consult our author's guidelines for proper formatting of your revised article for EMBO Molecular Medicine.

I look forward to receiving your revised manuscript.

Use this link to login to the manuscript system and submit your revision: https://embomolmed.msubmit.net/cgi-bin/main.plex

Kind regards,
Jingyi

Jingyi Hou
Senior Editor
EMBO Molecular Medicine

*****

When preparing your revised manuscript, please refer to our guidelines: https://link.springer.com/journal/44321/submission-guidelines#cms-Revised-submissions. We perform an initial quality control of all revised manuscripts before re-review; failure to include requested items will delay the evaluation of your revision.

We require:

1) A .docx formatted version of the manuscript text (including legends for main figures, EV figures and tables). Please make sure that the changes are highlighted to be clearly visible.

2) Individual production quality figure files as .eps, .tif, .jpg (one file per figure). For guidance, download the 'Figure Guide PDF': https://media.springernature.com/original/springer-cms/rest/v1/content/27825798/data/v1.

3) A .docx formatted letter INCLUDING the reviewers' reports and your detailed point-by-point responses to their comments. As part of the EMBO Press transparent editorial process, the point-by-point response is part of the Review Process File (RPF), which will be published alongside your paper.

4) A complete author checklist, which you can download from our author guidelines. Please insert information in the checklist that is also reflected in the manuscript. The completed author checklist will also be part of the RPF.

5) Please note that all corresponding authors are required to supply an ORCID ID for their name upon submission of a revised

manuscript.

6) It is mandatory to include a 'Data Availability' section after the Materials and Methods. Before submitting your revision, primary datasets produced in this study need to be deposited in an appropriate public database, and the accession numbers and database listed under 'Data Availability'. Please remember to provide a reviewer password if the datasets are not yet public.

In case you have no data that requires deposition in a public database, please state so in this section. Note that the Data Availability Section is restricted to new primary data that are part of this study.

7) For data quantification: please specify the name of the statistical test used to generate error bars and P values, the number (n) of independent experiments (specify technical or biological replicates) underlying each data point and the test used to calculate p-values in each figure legend. The figure legends should contain a basic description of n, P and the test applied. Graphs must include a description of the bars and the error bars (s.d., s.e.m.).

8) At EMBO Press we ask authors to provide source data for the main manuscript figures. You will receive a separate email with instructions for providing source data with your revised manuscript, including how to upload and organize the files.

9) Our journal encourages inclusion of *data citations in the reference list* to directly cite datasets that were re-used and obtained from public databases. Data citations in the article text are distinct from normal bibliographical citations and should directly link to the database records from which the data can be accessed. In the main text, data citations are formatted as follows: "Data ref: Smith et al, 2001" or "Data ref: NCBI Sequence Read Archive PRJNA342805, 2017". In the Reference list, data citations must be labeled with "[DATASET]". A data reference must provide the database name, accession number/identifiers and a resolvable link to the landing page from which the data can be accessed at the end of the reference.

10) We replaced Supplementary Information with Expanded View (EV) Figures and Tables that are collapsible/expandable online. A maximum of 5 EV Figures can be typeset. EV Figures should be cited as 'Figure EV1, Figure EV2" etc... in the text and their respective legends should be included in the main text after the legends of regular figures.

- For the figures that you do NOT wish to display as Expanded View figures, they should be bundled together with their legends in a single PDF file called *Appendix*, which should start with a short Table of Content. Appendix figures should be referred to in the main text as: "Appendix Figure S1, Appendix Figure S2" etc.

- Additional Tables/Datasets should be labeled and referred to as Table EV1, Dataset EV1, etc. Legends have to be provided in a separate tab in case of .xls files. Alternatively, the legend can be supplied as a separate text file (README) and zipped together with the Table/Dataset file.

11) The paper explained: EMBO Molecular Medicine articles are accompanied by a summary of the articles to emphasize the major findings in the paper and their medical implications for the non-specialist reader. Please provide a draft summary of your article highlighting

- the medical issue you are addressing,

- the results obtained and

- their clinical impact.

This may be edited to ensure that readers understand the significance and context of the research. Please refer to any of our published articles for an example.

12) Author contributions: You will be asked to provide CRediT (Contributor Role Taxonomy) terms in the submission system. These replace a narrative author contribution section in the manuscript.

13) A Conflict of Interest statement should be provided in the main text.

14) Please provide a 'Synopsis' to further enhance discoverability. Synopses are displayed on the journal webpage and are freely accessible to all readers. They include a short stand first (maximum of 300 characters, including space) as well as 2-5 one-sentences bullet points that summarizes the paper. Please write the bullet points to summarize the key NEW findings. They should be designed to be complementary to the abstract - i.e. not repeat the same text. We encourage inclusion of key acronyms and quantitative information (maximum of 30 words / bullet point). Please use the passive voice. Please attach these in a separate file or send them by email, we will incorporate them accordingly.

Please also provide a visual abstract to illustrate your article as a PNG file 550 px wide x 300 px high.

15) All Materials and Methods need to be described in the main text using our 'Structured Methods' format. According to this format, the Methods section includes a Reagents and Tools Table (listing key reagents, experimental models, software and relevant equipment and including their sources and relevant identifiers) followed by a Methods and Protocols section describing the methods, ideally using a step-by-step protocol format. The aim is to facilitate adoption of the methodologies across labs.

Please download and fill our Reagents and Tools Table template (.docx), which you can find in our author guidelines: https://link.springer.com/journal/44320/submission-guidelines#structuredmethods.

When submitting your revised manuscript, please do not include the Reagents and Tools Table in the Methods section of the manuscript but upload it as a separate file choosing the file type "Reagent Table".

EMBO Molecular Medicine has a "scooping protection" policy, whereby similar findings that are published by others during review or revision are not a criterion for rejection. Should you decide to submit a revised version, I do ask that you get in touch after three months if you have not completed it, to update us on the status.

***** Reviewer's comments *****

Referee #1 (Remarks for Author):

This manuscript by Hu, Li, Qi et al. investigates the mechanisms by which a ketogenic diet (KD) alleviates social deficits in the Shank3B knockout (KO) mouse model of autism spectrum disorder (ASD). The authors demonstrate that the ketone body β-hydroxybutyrate (BHB) is a key mediator of this effect. They elegantly show that BHB acts within the anterior cingulate cortex (ACC) to suppress the expression of HDAC9, a class IIa histone deacetylase that is selectively upregulated in this brain region. Using a combination of behavioral assays, electrophysiology, morphology, and viral-mediated gene transfer, they provide compelling evidence that the BHB-mediated suppression of HDAC9 rescues excitatory synaptic structure and function, thereby normalizing social behavior. The study is thorough, the data are of high quality, and the conclusions are largely supported by the evidence. This work identifies a novel metabolic-epigenetic pathway with significant therapeutic potential. Below are major and minor comments to improve the quality of this manuscript.
Major comments:
1)Causality of HDAC9 Upregulation in Shank3B KO Mice: The study definitively shows that HDAC9 is necessary and sufficient for the social deficits in the context of ACC manipulation. However, the mechanism linking the loss of Shank3B to the selective upregulation of HDAC9 in the ACC remains speculative. The authors propose a calcium/CaMKII signaling pathway (lines 324-330). While this is a plausible hypothesis, it is not directly tested. For instance, does restoring calcium influx or activating CaMKII in Shank3B KO neurons normalize HDAC9 expression? Including an experiment to test this link, even briefly, would significantly strengthen the paper.
2)The rationale for selecting BHB as the key metabolite from the metabolomics data should be made more explicit. Currently, the transition is plausible, but it would be helpful to state directly whether BHB was chosen because it was among the most strongly elevated metabolites, due to prior literature, or both.
3)The manuscript effectively demonstrates selective rescue. However, this point should be more consistently integrated into the overall framing. KD improved sociability and center time but did not affect grooming or locomotion. Conversely, HDAC9 overexpression induced social deficits without impacting anxiety-like or repetitive behaviors. This specificity should be emphasized rather than suggesting a broad normalization of ASD phenotypes.
4)The evidence for excitatory neuron associated HDAC9 upregulation is valuable; however, the language should remain appropriately scoped. Unless additional cell populations have been directly assessed, the text should avoid implying exclusivity.
5)The manuscript would benefit from a clearer explanation regarding the use of sex as a variable. The Methods section states that animals were matched by sex, age, and weight and housed in same-sex cages; however, it remains unclear whether both sexes were combined in each experiment and whether sex was included as a factor in the statistical analysis.

Minor comments:
1)The title may somewhat overstate the scope of the circuit-level rescue. The phrase "restore cortical circuit dysfunction" sounds broad, whereas the current dataset primarily focuses on ACC neuronal recruitment, synaptic transmission, and social behavior. A more precise title would better reflect the study's scope.
2)The interpretation of the c-FOS data should be approached with some nuance. Social interaction-induced c-FOS expression serves as an informative correlate of neuronal recruitment; however, it remains an indirect marker and should not be considered fully equivalent to a direct measurement of circuit restoration. Furthermore, while focusing on the ACC is reasonable and relevant, the Introduction and Discussion sections would benefit from a clearer and more explicit explanation of why this region was prioritized over other brain areas involved in social behavior.
3)The comparison between BHB and TMP269 in the Discussion should be toned down. TMP269 is used here as a class IIa HDAC inhibitor, but the text tends to imply HDAC9-specific pharmacology and suggests reduced off-target risks for BHB without direct supporting data. While the translational discussion is appealing, it should remain more cautious. The current manuscript occasionally refers to BHB as a "promising therapeutic candidate for ASD," yet the evidence is limited to one mouse model and a single principal symptom domain. A more measured translational statement would enhance the balance of the discussion.
4)There are several grammatical and typographical issues in the Discussion and Methods that should be corrected. Examples

include "that mediate ACC-specific social dysfunction" "by direct inhibiting" "focused on solely on" "administrated by gavage" "according to the manufacture" and so on. Moreover, the figure legends and main text still require a careful editorial-level consistency check. Panel labeling, figure numbering, "Scale bar" usage, and wording such as "mouse movement path diagram" should all be standardized before acceptance.
5)The virus description would benefit from additional detail. For example, the AAV titer is provided for sparse labeling but not clearly specified for the ACC overexpression virus. It would also be helpful to include the injection volume per side and to clarify whether the injection sites were histologically verified in all animals.
6)The Key Resources/Reagents table should be cleaned up. There are duplicate entries (e.g., Rabbit anti-GFP appears twice; BCA Protein Assay Kit appears twice with two different catalog numbers), and some source formatting is inconsistent.
7)The figure legends require a thorough consistency check. There appear to be several errors in panel labeling and figure referencing, including the inclusion of metabolomics panels K-P in the Figure 1 legend, despite these data being presented as Figure 2 in the Results section; the repeated use of panel D in Figure 3; and inconsistent panel assignments in Figure 7. These presentation issues should be corrected throughout the manuscript.

Referee #2 (Remarks for Author):

The study entitled "β-hydroxybutyrate suppresses HDAC9 to restore cortical circuit dysfunction in Shank3B-deficient mice" investigates the mechanisms underlying the therapeutic effects of the ketogenic diet (KD) in a Shank3 knockout (KO) mouse model of autism. The authors show that KD improves social deficits in Shank3 KO mice. Furthermore, β-hydroxybutyrate (BHB), a major metabolic product of KD, partially mimics the effects of KD treatment by enhancing the structure and function of excitatory synapses on pyramidal neurons in the anterior cingulate cortex (ACC). The study further identifies histone deacetylase 9 (HDAC9) as a potential molecular target of BHB treatment. Pharmacological inhibition of class IIa HDACs and neuronal overexpression experiments suggest a role for HDAC9 in regulating social and synaptic phenotypes. Overall, the work provides interesting insights into a possible BHB-HDAC pathway linking metabolic intervention to synaptic function in ASD models. However, the manuscript would benefit from a more cautious interpretation of the proposed mechanism, improved methodological and statistical transparency, and correction of several presentation-related issues.
Specific comments
1. In the Abstract, the statement "highlighting HDAC9 as a therapeutically actionable target for ASD" (line 43) should be moderated, as the current findings are limited to a single mouse model rather than the broader ASD spectrum.
2. In the Introduction, the sentence "Emerging evidence has linked between ASD and systemic metabolic disturbances in the brain" (line 58) is grammatically incorrect and conceptually unclear. The authors should distinguish between systemic metabolic alterations and brain-specific metabolic dysregulation.
3. Also in the Introduction, the phrase "both pharmacological inhibition of HDAC9 and direct neuronal overexpression" (line 91) requires revision. TMP269 is a class IIa HDAC inhibitor and does not selectively target HDAC9.
4. In the Results section, the sentence "To evaluate the therapeutic potential of a KD on for core autism-related behaviors" (line 98) contains a clear language error and should be corrected.
5. The language describing behavioral effects in the Results section should be moderated. Statements such as "KD administration completely reversed these deficits" (line 105) and "short-term BHB treatment was sufficient to fully recapitulate the prosocial effects of the chronic KD" (line 146) appear overstated unless formal equivalence testing was performed.
6. The sentence "western blot analysis of ACC tissue reversed that HDAC9 overexpression significantly downregulated..." (line 208) is clearly incorrect and should be revised.
7. In the Discussion, the statement that "HDAC9's role appears both necessary and sufficient..." (line 306) is not fully supported by the current data. Evidence for necessity would require knockdown, knockout, or rescue experiments.
8. The wording regarding TMP269 should be more precise throughout the manuscript. Although TMP269 inhibits class IIa HDACs, the text occasionally implies HDAC9-specific pharmacology. The distinction between class IIa HDAC inhibition and HDAC9-specific regulation should be maintained.
9. The Methods section should clarify how sex was considered as a biological variable. Please specify whether males and females were analyzed separately, balanced across groups, or pooled, and provide justification for the chosen approach.
10. For Figure 4, the legend should describe in greater detail how CaMKII colocalization was quantified to support the localization claim.
11. The statistical analysis requires clarification, particularly for Figures 5-7. Please indicate whether statistical tests were performed on individual cell-level measurements or on mouse-level averages, given the relatively small number of animals but large number of cells analyzed (e.g., mEPSCs, calcium imaging, spine density).
12. Several errors are present in the figure legends. In Figure 1, metabolomics panels K-P are listed even though these data appear in Figure 2. Figure 3 contains duplicate panel labels for D. In Figure 7, mEPSC amplitude is labeled as Q, followed immediately by Q-S for calcium imaging. These inconsistencies should be corrected.
13. For the calcium imaging experiments (Figures 6 and 7), the analysis pipeline should be described in greater detail. Please specify the metrics used (e.g., peak ΔF/F, area under the curve, responder fraction), criteria for cell selection, and confirm that identical criteria were applied across all experimental groups.
14. Figure legends should be further revised to correct typographical errors (e.g., "Scal bar"), awkward wording such as "mouse movement path diagram," and inaccurate descriptions such as "center time/distance measures" in Figure 7.
15. The Key Resources table should be revised to remove duplicate entries (e.g., Rabbit anti-GFP antibody and BCA kits) to

improve clarity and accuracy.
16. The discussion of translational relevance should be more cautious. Statements positioning BHB as a therapeutic candidate for ASD should be tempered, given that the conclusions rely on data from a single mouse model.

Detailed Response to Reviewers

We sincerely thank the editor and both reviewers for their careful, detailed, and constructive evaluation of our manuscript. Their comments were extremely helpful in identifying aspects of the mechanistic interpretation, statistical analysis, methodological transparency, figure presentation, and translational framing that required clearer explanation, additional experimental support, or more cautious wording.

In response, we have substantially revised the manuscript. Specifically, we performed additional experiments to strengthen the mechanistic link between Ca^{2+} signaling and HDAC9 dysregulation, clarified the rationale for focusing on BHB and the ACC, and moderated statements where the conclusions could have been overstated. We also systematically re-examined the statistical analyses throughout the manuscript, corrected and refined the significance annotations in the figures where necessary, and added a comprehensive statistical summary table listing the statistical method used for each analysis (**Table EV2**). In parallel, we revised the Results, figure legends, Methods, and Discussion to ensure that the textual descriptions are fully consistent with the statistical outcomes and that the conclusions are presented with appropriate rigor and caution.

Overall, we believe that these revisions have significantly strengthened the rigor, clarity, and presentation of the work, and we are grateful for the opportunity to improve the manuscript through this review process.

Following are our point-by-point responses to the comments on our manuscript (**Manuscript Number: EMM-2026-23556**).

Editors

Issue 1: *A .docx formatted version of the manuscript text (including legends for main figures, EV figures and tables). Please make sure that the changes are highlighted to be clearly visible.*

Response: We thank you for this instruction. We have prepared and uploaded a revised .docx version of the manuscript text, including the legends for all main figures, EV figures, and tables. All corresponding changes have been highlighted in **Red** in the revised manuscript for your convenience.

Issue 2: *Individual production quality figure files as .eps, .tif, .jpg (one file per figure). For guidance, download the 'Figure Guide PDF': https://media.springernature.com/original/springer-cms/rest/v1/content/27825798/data/v1.*

Response: We thank you for this instruction. We have uploaded the individual production-quality figure files in the required formats, with one file provided for each figure, in accordance with the journal's figure preparation guidelines.

Issue 3: *A .docx formatted letter INCLUDING the reviewers' reports and your detailed point-by-point responses to their comments. As part of the EMBO Press transparent editorial process, the point-by-point response is part of the Review Process File (RPF), which will be published alongside your paper.*

Response: We thank you for this instruction. We have prepared and uploaded a .docx-formatted point-by-point response letter that includes the reviewers' reports together with our detailed responses to each comment. We understand that this document will form part of the Review Process File (RPF) and have therefore carefully revised it for clarity and completeness.

Issue 4: *A complete author checklist, which you can download from our author guidelines. Please insert information in the checklist that is also reflected in the manuscript. The completed author checklist will also be part of the RPF.*

Response: We thank you for this instruction. We have completed the author checklist and ensured that the information provided there is consistent with the revised manuscript. The completed checklist has been included with the revised submission, and we understand that it will also form part of the RPF.

Issue 5: *Please note that all corresponding authors are required to supply an ORCID ID for their name upon submission of a revised manuscript.*

Response: We thank you for this reminder. We have provided the ORCID IDs of the corresponding authors in the revised manuscript.

Issue 6: *It is mandatory to include a 'Data Availability' section after the Materials and Methods. Before submitting your revision, primary datasets produced in this study need to be deposited in an appropriate public database, and the accession numbers and database listed under 'Data Availability'. Please remember to provide a reviewer password if the datasets are not yet public.*

Response: We thank you for this important instruction. A dedicated Data Availability section has now been added after the Materials and Methods in the revised manuscript. In this section, we state that the metabolomics data generated in this study have been deposited in the MetaboLights database under accession number MTBLS14381. As the dataset is not yet public, reviewer access details will be provided to facilitate confidential evaluation.

Issue 7: *In case you have no data that requires deposition in a public database, please state so in this section. Note that the Data Availability Section is restricted to new primary data that are part of this study.*

Response: We thank you for this clarification. In the revised manuscript, the Data Availability section has been restricted to the new primary data generated in this study. Specifically, we have included the deposition information for the metabolomics dataset generated in this work and have not included unrelated data types that do not require public deposition.

Issue 8: *For data quantification: please specify the name of the statistical test used to generate error bars and P values, the number (n) of independent experiments (specify technical or biological replicates) underlying each data point and the test used to calculate p-values in each figure legend. The figure legends should contain a basic description of n, P and the test applied. Graphs must include a description of the bars and the error bars (s.d., s.e.m.).*

Response: We thank you for this important instruction. We have fully revised all figure legends in the updated manuscript to comply with the requirements. Specifically, each legend now clearly states the statistical tests for error bars and P values, defines the number of independent replicates (n) and distinguishes biological/technical replicates for every data point. Meanwhile, we have revised the Statistical Analysis section to enhance methodological transparency across the study. For datasets from cells or dendritic structures, we additionally specified the total number of analyzed cells/dendrites and the number of donor mice in corresponding legends. All statistical analyses throughout the manuscript have been rechecked, and relevant figure annotations have been updated accordingly. **To further improve clarity, we have added a statistical summary table (Table EV2).** We believe these revisions make the data quantification and statistical reporting substantially clearer and more consistent with the journal's requirements.

Issue 9: *At EMBO Press we ask authors to provide source data for the main manuscript figures. You will receive a separate email with instructions for providing source data with your revised manuscript, including how to upload and organize the files.*

Response: We thank you for this instruction. We have prepared the source data for the main manuscript figures and will upload and organize the files according to the journal's guidelines.

Issue 10: *Our journal encourages inclusion of *data citations in the reference list* to directly cite datasets that were re-used and obtained from public databases. Data citations in the article text are distinct from normal bibliographical citations and should directly link to the database records from which the data can be accessed. In the main text, data citations are formatted as follows: "Data ref: Smith et al, 2001" or "Data ref: NCBI Sequence Read Archive PRJNA342805, 2017". In the Reference list, data citations must be labeled with "[DATASET]". A data reference must provide the database name, accession number/identifiers and a resolvable link to the landing page from which the data can be accessed at the end of the reference.*

Response: We thank you for this helpful guidance. The present study does not include re-used datasets obtained from public databases. Therefore, no additional data citations were required in the reference list.

Issue 11: *We replaced Supplementary Information with Expanded View (EV) Figures and Tables that are collapsible/expandable online. A maximum of 5 EV Figures can be typeset. EV Figures should be cited as 'Figure EV1, Figure EV2" etc... in the text and their respective legends should be included in the main text after the legends of regular figures.*

Response: We thank you for this clarification. We have reorganized the supplementary materials into Expanded View (EV) Figures and Tables in accordance with the journal's format. The EV figures are now cited in the main text as Figure EV1, Figure EV2, etc., and their corresponding legends have been included in the main manuscript after the legends of the main figures.

Issue 12: *The paper explained: EMBO Molecular Medicine articles are accompanied by a summary of the articles to emphasize the major findings in the paper and their medical implications for the non-specialist reader. Please provide a draft summary of your article highlighting*

- the medical issue you are addressing,

- the results obtained and

- their clinical impact.

This may be edited to ensure that readers understand the significance and context of the research. Please refer to any of our published articles for an example.

Response: We thank you for this helpful instruction. We have prepared a draft summary of our article for the non-specialist reader, highlighting the medical issue addressed, the main findings, and their potential clinical relevance, and have included it with the revised submission.

Issue 13: *Author contributions: You will be asked to provide CRediT (Contributor Role Taxonomy) terms in the submission system. These replace a narrative author contribution section in the manuscript.*

Response: We thank you for this clarification. We confirm that the CRediT terms had already been provided in the submission system at the time of the initial submission.

Issue 14: *A Conflict of Interest statement should be provided in the main text.*

Response: We thank you for this reminder. A Conflict of Interest statement was already included in the original submitted manuscript.

Issue 15: *Please provide a 'Synopsis' to further enhance discoverability. Synopses are displayed on the journal webpage and are freely accessible to all readers. They include a short stand first (maximum of 300 characters, including space) as well as 2-5 one-sentences bullet points that summarizes the paper. Please write the bullet points to summarize the key NEW findings. They should be designed to be complementary to the abstract - i.e. not repeat the same text. We encourage inclusion of key acronyms and quantitative information (maximum of 30 words / bullet point). Please use the passive voice. Please attach these in a separate file or send them by email, we will incorporate them accordingly.*

Response: We thank you for this helpful instruction. We have prepared a Synopsis, including a short standfirst and a set of bullet points summarizing the key new findings of the study in a form complementary to the Abstract. This material has been provided as a separate file with the revised submission.

Issue 16: *Please also provide a visual abstract to illustrate your article as a PNG file 550 px wide x 300 px high.*

Response: We thank you for this instruction. A visual abstract illustrating the main findings of the study has been prepared in the required PNG format (550 px × 300 px) and has been included with the revised submission.

Issue 17: *All Materials and Methods need to be described in the main text using our 'Structured Methods' format. According to this format, the Methods section includes a Reagents and Tools Table (listing key reagents, experimental models, software and relevant equipment and including their sources and relevant identifiers) followed by a Methods and Protocols section describing the methods, ideally using a step-by-step protocol format. The aim is to facilitate adoption of the methodologies across labs.*

Response: We thank you for this important instruction. In the revised manuscript, we have reorganized the Materials and Methods section to follow the journal's Structured Methods format. In addition, the corresponding Reagents and Tools Table has been prepared separately in the journal's required format and included with the revised submission.

Issue 18: *Please download and fill our Reagents and Tools Table template (.docx), which you can find in our author guidelines. When submitting your revised manuscript, please do not include the Reagents and Tools Table in the Methods section of the manuscript but upload it as a separate file choosing the file type "Reagent Table".*

Response: We thank you for this instruction. We have completed the Reagents and Tools Table using the journal's template and have uploaded it as a separate file under the file type "Reagent Table".

Issue 19: *EMBO Molecular Medicine has a "scooping protection" policy, whereby similar findings that are published by others during review or revision are not a criterion for rejection. Should you decide to submit a revised version, I do ask that you get in touch after three months if you have not completed it, to update us on the status.*

Response: We thank you for this clarification and appreciate the journal's scooping protection policy. We have completed the revision and are now submitting the revised manuscript accordingly.

Reviewer 1

This manuscript by Hu, Li, Qi et al. investigates the mechanisms by which a ketogenic diet (KD) alleviates social deficits in the Shank3B knockout (KO) mouse model of autism spectrum disorder (ASD). The authors demonstrate that the ketone body β-hydroxybutyrate (BHB) is a key mediator of this effect. They elegantly show that BHB acts within the anterior cingulate cortex (ACC) to suppress the expression of HDAC9, a class IIa histone deacetylase that is selectively upregulated in this brain region. Using a combination of behavioral assays, electrophysiology, morphology, and viral-mediated gene transfer, they provide compelling evidence that the BHB-mediated suppression of HDAC9 rescues excitatory synaptic structure and function, thereby normalizing social behavior. The study is thorough, the data are of high quality, and the conclusions are largely supported by the evidence. This work identifies a novel metabolic-epigenetic pathway with significant therapeutic potential. Below are major and minor comments to improve the quality of this manuscript.

Response: We sincerely thank you for the thoughtful, constructive, and encouraging assessment of our work. We have carefully addressed all of the comments raised and have revised the manuscript accordingly. All corresponding changes have been highlighted in **Red** in the revised manuscript for your convenience. Detailed point-by-point responses are provided below.

Issue 1: *Causality of HDAC9 Upregulation in Shank3B KO Mice: The study definitively shows that HDAC9 is necessary and sufficient for the social deficits in the context of ACC manipulation. However, the mechanism linking the loss of Shank3B to the selective upregulation of HDAC9 in the ACC remains speculative. The authors propose a calcium/CaMKII signaling pathway (lines 324-330). While this is a plausible hypothesis, it is not directly tested. For instance, does restoring calcium influx or activating CaMKII in Shank3B KO neurons normalize HDAC9 expression? Including an experiment to test this link, even briefly, would significantly strengthen the paper.*

Response: We sincerely thank you for this insightful suggestion. We agree that direct experimental testing of the proposed Ca^{2+}/CaMKII–HDAC9 link would strengthen the mechanistic interpretation of the study.

To address this point, we performed a new experiment to test whether enhancing Ca^{2+} influx could normalize HDAC9 expression in *Shank3B*-deficient neurons. Primary cultured cortical neurons from WT and *Shank3B* KO mice were treated with the L-type Ca^{2+} channel agonist Bay K8644. We found that KO neurons displayed elevated HDAC9 immunofluorescence intensity and increased *Hdac9* mRNA expression compared with WT neurons under vehicle-treated conditions. Importantly, Bay K8644 treatment significantly reduced both HDAC9 protein signal and *Hdac9* mRNA expression in *Shank3B* KO neurons, shifting them toward WT-like levels, while having relatively limited effects in WT neurons. These new data are now included as **Figure EV4**.

We have also revised the Results (**see page 8, lines 252-259**) and Discussion (**see page 12, lines 365-384**) sections to incorporate these findings and to temper our mechanistic interpretation. Specifically, we now state that impaired Ca^{2+}-dependent signaling may contribute to HDAC9 dysregulation in *Shank3B*-deficient neurons, rather than claiming that the Ca^{2+}/CaMKII–HDAC9 pathway has been definitively established. We further acknowledge that future experiments directly measuring HDAC9 phosphorylation, nucleocytoplasmic localization, and CaMKII dependence in ACC neurons in vivo will be required to fully establish this pathway.

Together, these new data provide experimental support for the reviewer's proposed link between altered Ca^{2+} influx and HDAC9 upregulation, while maintaining an appropriately cautious interpretation of the underlying mechanism.

Issue 2: *The rationale for selecting BHB as the key metabolite from the metabolomics data should be made more explicit. Currently, the transition is plausible, but it would be helpful to state directly whether BHB was chosen because it was among the most strongly elevated metabolites, due to prior literature, or both.*

Response: We sincerely thank you for this important comment. In the revised manuscript, we have clarified that BHB was selected based on both its marked elevation following KD treatment and its strong mechanistic relevance from prior literatures (**Newman & Verdin, 2017, Zhu H *et al*, 2022**). Specifically, among the metabolites altered by KD, BHB was one of the most prominently increased ketone bodies and showed a substantially greater fold change than

many other detected metabolites. In addition, BHB has been well documented as a biologically active ketone body with established roles in epigenetic regulation, neuronal function, and neuroprotection, making it a particularly strong candidate mediator of the behavioral and synaptic effects of KD. We therefore revised the relevant text to state this rationale explicitly, so that the transition from the metabolomics data to the focused analysis of BHB is more transparent to the reader (**see page 5, lines 153–155**).

Issue 3: *The manuscript effectively demonstrates selective rescue. However, this point should be more consistently integrated into the overall framing. KD improved sociability and center time but did not affect grooming or locomotion. Conversely, HDAC9 overexpression induced social deficits without impacting anxiety-like or repetitive behaviors. This specificity should be emphasized rather than suggesting a broad normalization of ASD phenotypes.*

Response: Thank you for this constructive comment. We agree that our data support a selective, rather than global, rescue of ASD-related phenotypes, and that this point should be integrated more consistently into the overall framing of the manuscript. In response, we have revised the manuscript at multiple levels to better reflect this specificity.

First, we revised the title to focus specifically on social deficits and the ACC, rather than overgeneralizing to "cortical circuit dysfunction" or global ASD normalization. The new title is "**β-hydroxybutyrate Restores Social Deficits by Suppressing HDAC9 in the ACC of *Shank3B*-deficient Mice**" (**see page 1, lines 1-2**). Second, we revised the final sentence of the Abstract to specify that HDAC9 is relevant to social deficits in ASD-related pathology, rather than ASD broadly (**see page 2, lines 44-45**). Third, although our original results already described this selectivity, we agree that the overall framing could still be interpreted too broadly. We have therefore refined the Discussion and softened statements that might imply broad ASD normalization. The revised text now consistently emphasizes that the BHB–HDAC9 axis in the ACC exerts a circuit- and behavior-selective effect on social deficits, without affecting repetitive grooming (**see page 9, lines 286-293**).

We believe these revisions now better align the framing of the manuscript with the actual scope of the data.

Issue 4: *The evidence for excitatory neuron associated HDAC9 upregulation is valuable; however, the language should remain appropriately scoped. Unless additional cell populations have been directly assessed, the text should avoid implying exclusivity.*

Response: We sincerely thank you for this important comment. We agree that the language in the original manuscript may have overstated the cell-type specificity of our findings.

In the present study, the evidence for excitatory neuron involvement comes primarily from the single-cell qPCR analysis, which showed increased Hdac9 mRNA levels in ACC excitatory neurons from *Shank3B* KO mice. In contrast, the qPCR and western blot data were obtained from bulk ACC tissue and therefore reflect overall changes at the tissue level rather than cell-type-specific expression. We agree that these tissue-level results do not exclude the possible contribution of other cellular populations in the ACC.

Accordingly, we have revised the relevant wording throughout the manuscript to avoid implying cell-type exclusivity. Specifically, we now state that our data indicate elevated Hdac9 mRNA in ACC excitatory neurons at the single-cell level, together with overall upregulation of HDAC9 in ACC tissue, rather than suggesting that the increase is exclusively restricted to excitatory neurons (**see pages 7, lines 196, 202-203**). In addition, to better reflect the cellular complexity of the ACC, we have revised the Discussion to note that recent studies have highlighted the contribution of ACC interneuron subtypes, including parvalbumin and somatostatin interneurons, to the regulation of social behavior (**see page 11, lines 353-359**). This addition was intended to emphasize that ACC micro-circuit is likely important for ASD-related social dysfunction, while also making clear that the present study did not directly assess interneuron-specific HDAC9 regulation. We believe this revised phrasing more accurately reflects the scope of the current evidence.

Issue 5: *The manuscript would benefit from a clearer explanation regarding the use of sex as a variable. The Methods section states that animals were matched by sex, age, and weight and housed in same-sex cages; however, it remains unclear whether both sexes were combined in each experiment and whether sex was included as a factor in the statistical analysis.*

Response: We sincerely thank you for this important suggestion. We agree that the treatment of sex as a biological variable should be described more clearly. In the revised Methods, we have clarified the sex composition of the animals used in each experiment and explicitly stated whether male and female mice were pooled or analyzed separately. We have also clarified whether sex was included as a factor in the statistical analysis. In experiments where males and females were pooled, this is now stated explicitly, and we have revised the text to make the rationale and analytical approach clear (**see page 14, lines 428-433**). We believe these revisions improve the transparency of the experimental design and more accurately describe how sex was handled in the present study.

Issue 6: *The title may somewhat overstate the scope of the circuit-level rescue. The phrase "restore cortical circuit dysfunction" sounds broad, whereas the current dataset primarily focuses on ACC neuronal recruitment, synaptic transmission, and social behavior. A more precise title would better reflect the study's scope.*

Response: We sincerely thank you for this insightful comment. To better reflect the actual scope and findings of our work, we have revised the title accordingly. The new title is **"β-hydroxybutyrate Restores Social Deficits by Suppressing HDAC9 in the ACC of *Shank3B*-deficient Mice**". We believe this revised title more accurately captures the region-specific and behavior-specific nature of our findings, which center on ACC-associated synaptic and neuronal changes linked to social behavior, rather than broad restoration of cortical circuit dysfunction..

Issue 7: *The interpretation of the c-FOS data should be approached with some nuance. Social interaction-induced c-FOS expression serves as an informative correlate of neuronal recruitment; however, it remains an indirect marker and should not be considered fully equivalent to a direct measurement of circuit restoration. Furthermore, while focusing on the ACC is reasonable and relevant, the Introduction and Discussion sections would benefit from a clearer and more explicit explanation of why this region was prioritized over other brain areas involved in social behavior.*

Response: We sincerely thank you for this thoughtful comment.

First, we agree that social interaction-induced c-FOS expression should be interpreted with appropriate caution. In the present study, we used c-FOS only as an informative indicator of neuronal recruitment associated with social behavior, not as a direct measure of full circuit restoration. In response, we revised the relevant text in both the Results and the Discussion to avoid overstating the implications of the c-FOS data. Specifically, in the Results section, we replaced wording that could have been interpreted as equating the recovery of c-FOS induction with direct restoration of cortical circuit function (**see page 6, lines 171-177**). In the Discussion, we further revised the text to state that the BHB-associated increase in social interaction-induced c-FOS expression in the ACC is consistent with improved recruitment of ACC neurons during social behavior, while also making clear that c-FOS remains an indirect marker of neuronal activity rather than a direct measure of complete circuit reinstatement (**see page 12, lines 384-388**). The revised language now clearly states that c-FOS reflects neuronal activity changes, not complete circuit reinstatement.

Second, regarding the rationale for prioritizing the ACC over other brain areas involved in social behavior.We fully agree that the Introduction and Discussion sections should more explicitly explain why the ACC was chosen as the primary region of focus. In response, we have substantially strengthened both sections. In the Introduction, we have added a dedicated paragraph (**see page 3, lines 82–93**). This paragraph: (1) emphasizes that, because ASD involves multi-system biological and neural disruptions, understanding how ketogenic metabolites act on socially relevant brain hubs is important for defining therapeutic mechanisms; (2) notes that, although several brain regions have been implicated in social behavior, including the hippocampus, ACC, striatum, and paraventricular nucleus, the ACC was prioritized because it is a well-established hub for social salience, empathy, and social decision-making and has been consistently linked to social deficits in ASD **(Sato *et al.*, 2023; Apps *et al.*, 2016; Balsters *et al.*, 2017; Urbain *et al.*, 2015; Willbrand *et al.*, 2026)**; and (3) highlights our previous finding that ACC dysfunction drives social impairments in *Shank3B* KO mice, whereas selective activation of ACC pyramidal neurons rescues these deficits, thereby providing a direct experimental rationale for focusing on the ACC in the present study (**Guo *et al.*, 2019**). This

provides a clear, step-by-step justification for why the ACC was prioritized in the current mechanistic investigation.

In the Discussion, we further added explicit statements to clarify our regional focus on the ACC (**see pages 11-12, lines 346–361**). Specifically, the revised text now emphasizes that: (1) the ACC is a key node for social information processing and has been linked to social deficits in autism; (2) although our regional survey was not exhaustive, we examined several major brain regions implicated in social behavior, including the hippocampus, striatum, and ACC, and found that HDAC9-related changes were most evident in the ACC, supporting its relative regional specificity in the present model; and (3) the cellular heterogeneity of the ACC, including distinct interneuron subtypes such as parvalbumin and somatostatin interneurons, is now recognized to shape social behavior, indicating that multiple cell populations may contribute to ASD-related social dysfunction. At the same time, we explicitly state that, although our current data primarily implicate ACC excitatory neurons and overall ACC tissue-level HDAC9 dysregulation, they do not exclude possible contributions from interneuron populations.

We believe this clarification makes the regional rationale clearer and better explains why the ACC, rather than other brain regions involved in social behavior, was emphasized in the present study.

Issue 8: *The comparison between BHB and TMP269 in the Discussion should be toned down. TMP269 is used here as a class IIa HDAC inhibitor, but the text tends to imply HDAC9-specific pharmacology and suggests reduced off-target risks for BHB without direct supporting data. While the translational discussion is appealing, it should remain more cautious. The current manuscript occasionally refers to BHB as a "promising therapeutic candidate for ASD," yet the evidence is limited to one mouse model and a single principal symptom domain. A more measured translational statement would enhance the balance of the discussion.*

Response: We sincerely thank you this important and insightful comment. We agree that both the comparison between BHB and TMP269 and the broader translational framing in the

Discussion should be presented more cautiously. In response, we revised the Discussion in several specific places.

First, in the opening paragraph of the Discussion, we moderated the overall translational tone of the study. In the revised version, we softened the statement that KD alleviates social deficits in this model, that BHB is an important mediator of these effects, and that our data suggest BHB is associated with the normalization of HDAC9 expression, along with the restoration of excitatory synaptic structure and function (**see page 9, lines 286-293**). This change was made to avoid overstating mechanistic certainty and translational scope.

Second, we substantially revised the third paragraph of the Discussion, where the comparison between BHB and TMP269 was originally too strong. In the revised version, we made this comparison more cautious in several ways: (1) BHB is now described as a potentially more practical experimental strategy for further investigation, rather than being positioned too directly as a therapeutic option; (2) the wording was changed from "largely overlapping benefits" to "partially overlapping effects"; (3) we explicitly state that TMP269 does not provide HDAC9-specific pharmacological evidence; and (4) we removed the statement suggesting reduced off-target risks for BHB relative to TMP269, because this was not directly tested in the present study (**see pages 10-11, lines 316-318, and 320-329**). Together, these revisions more clearly distinguish class IIa HDAC pathway-level pharmacological support from the more specific implication of HDAC9 derived from our molecular and viral overexpression data.

Third, we also revised the final summary paragraph of the Discussion to reduce overstatement of therapeutic implications. In the revised version, we stated that the findings support a role for HDAC9-related mechanisms in ASD-related social dysfunction in this model and provide a framework for further investigation in additional preclinical models and human-relevant systems (**see page 13, lines 418-421**). This revised wording better reflects the fact that the current conclusions are based on a single mouse model and should not be generalized too broadly.

We believe these revisions make the Discussion more balanced and better aligned with the actual scope of the current data.

Issue 9: *There are several grammatical and typographical issues in the Discussion and Methods that should be corrected. Examples include "that mediate ACC-specific social dysfunction" "by direct inhibiting" "focused on solely on" "administrated by gavage" "according to the manufacture" and so on. Moreover, the figure legends and main text still require a careful editorial-level consistency check. Panel labeling, figure numbering, "Scale bar" usage, and wording such as "mouse movement path diagram" should all be standardized before acceptance.*

Response: We sincerely thank you for this careful and constructive comment. We agree that the manuscript required additional language polishing and a more thorough editorial-level consistency check. In the revised version, we have carefully proofread the full manuscript and corrected the grammatical and typographical issues identified by the reviewer, including all of the examples noted above. Specifically, wording such as "that mediate ACC-specific social dysfunction" (corrected to "that mediates…"), "by direct inhibiting" (corrected to "by directly inhibiting"), "focused on solely on" (corrected to "focused solely on"), "administrated by gavage" (corrected to "administered by gavage"), and "according to the manufacture" (corrected to "according to the manufacturer") has been corrected in the revised manuscript.

In addition, we have thoroughly revised the figure legends and the corresponding main text to improve consistency and presentation. This included standardizing panel labeling, figure numbering, terminology, and formatting across the manuscript. We also corrected repeated issues such as "Scal bar" to "Scale bar" and replaced awkward expressions such as "mouse movement path diagram" with more appropriate wording.

We believe these revisions have substantially improved the clarity, consistency, and overall presentation of the manuscript.

Issue 10: *The virus description would benefit from additional detail. For example, the AAV titer is provided for sparse labeling but not clearly specified for the ACC overexpression virus. It would also be helpful to include the injection volume per side and to clarify whether the injection sites were histologically verified in all animals.*

Response: We sincerely thank your for this helpful suggestion. We agree that the description of the viral procedures should be more complete and transparent. In the revised Methods, we have added the missing experimental details for the ACC overexpression virus, including the viral titer (1×10^{12} GC/ml) and the injection volume per side (200 nL). We have also clarified that the stereotaxic injection sites were histologically verified in the animals included in the corresponding analyses. In addition, representative images showing the verification of viral expression and injection sites have now been provided in Figure EV1A. The revised text has been incorporated into the Virus Injection subsection of the Methods (**see page 14, lines 438-439, and 444-446**). We believe these additions improve the reproducibility of the study and make the viral manipulation procedures clearer to the reader.

Issue 11: *The Key Resources/Reagents table should be cleaned up. There are duplicate entries (e.g., Rabbit anti-GFP appears twice; BCA Protein Assay Kit appears twice with two different catalog numbers), and some source formatting is inconsistent.*

Response: We sincerely thank you for identifying this issue. In the revised manuscript, we have removed duplicate entries, including the repeated Rabbit anti-GFP antibody and BCA Protein Assay Kit entries, corrected inconsistencies in catalog numbers and source information, and standardized the formatting throughout the table.

Issue 12: *The figure legends require a thorough consistency check. There appear to be several errors in panel labeling and figure referencing, including the inclusion of metabolomics panels K-P in the Figure 1 legend, despite these data being presented as Figure 2 in the Results section; the repeated use of panel D in Figure 3; and inconsistent panel assignments in Figure 7. These presentation issues should be corrected throughout the manuscript.*

Response: We sincerely thank you for carefully identifying these presentation inconsistencies. In the revised manuscript, we have carefully reviewed and corrected the figure legends, panel labeling, and the corresponding in-text figure citations throughout the manuscript. Specifically, we corrected the misplaced metabolomics panels in the Figure 1 legend, resolved the duplicated panel D labeling in Figure 3, and corrected the inconsistent panel assignments in Figure 7. We

also performed a full cross-check of all figures and legends to ensure consistency between the Results section and the final figure set.

Reviewer 2

The study entitled "β-hydroxybutyrate suppresses HDAC9 to restore cortical circuit dysfunction in Shank3B-deficient mice" investigates the mechanisms underlying the therapeutic effects of the ketogenic diet (KD) in a Shank3 knockout (KO) mouse model of autism. The authors show that KD improves social deficits in Shank3 KO mice. Furthermore, β-hydroxybutyrate (BHB), a major metabolic product of KD, partially mimics the effects of KD treatment by enhancing the structure and function of excitatory synapses on pyramidal neurons in the anterior cingulate cortex (ACC). The study further identifies histone deacetylase 9 (HDAC9) as a potential molecular target of BHB treatment. Pharmacological inhibition of class IIa HDACs and neuronal overexpression experiments suggest a role for HDAC9 in regulating social and synaptic phenotypes. Overall, the work provides interesting insights into a possible BHB-HDAC pathway linking metabolic intervention to synaptic function in ASD models. However, the manuscript would benefit from a more cautious interpretation of the proposed mechanism, improved methodological and statistical transparency, and correction of several presentation-related issues.

Response: We are grateful for your recognition of the interest and potential significance of this work, as well as for the helpful suggestions regarding mechanistic interpretation, methodological clarity, statistical transparency, and presentation. All corresponding changes have been highlighted in **Red** in the revised manuscript for your convenience. Detailed point-by-point responses are provided below.

Issue 1: *In the Abstract, the statement "highlighting HDAC9 as a therapeutically actionable target for ASD" (line 43) should be moderated, as the current findings are limited to a single mouse model rather than the broader ASD spectrum.*

Response: We sincerely thank you for this important suggestion. We agree that the original wording in the Abstract was too broad given that the current findings are based on a single mouse model. In response, we revised the final sentence of the Abstract to moderate the translational scope of the statement. The sentence now reads: **"These findings uncover a metabolite-driven epigenetic mechanism linking ketogenic metabolism to the rescue of**

social behavior via the ACC, and identify HDAC9 as a potential therapeutic target for ASD-related social deficits." (**see page 2, lines 44-45**). We believe this revised wording better reflects the scope of the present data while preserving the translational relevance of the findings.

Issue 2: *In the Introduction, the sentence "Emerging evidence has linked between ASD and systemic metabolic disturbances in the brain" (line 58) is grammatically incorrect and conceptually unclear. The authors should distinguish between systemic metabolic alterations and brain-specific metabolic dysregulation.*

Response: We sincerely thank you for this helpful suggestion. In the revised manuscript, we have rewritten this sentence to avoid conflating systemic metabolic alterations with brain-specific metabolic dysregulation. The sentence now reads: **"Emerging evidence has linked ASD to metabolic disturbances in the brain."** (**see page 2, line 59**). We believe this revision improves both the grammatical correctness and the conceptual clarity of the Introduction.

Issue 3: *Also in the Introduction, the phrase "both pharmacological inhibition of HDAC9 and direct neuronal overexpression" (line 91) requires revision. TMP269 is a class IIa HDAC inhibitor and does not selectively target HDAC9.*

Response: We sincerely thank you for this important comment. We agree that the original phrasing was inaccurate, because TMP269 is a class IIa HDAC inhibitor and does not selectively target HDAC9. In the revised manuscript, we have revised the sentence in the Introduction to read **"both pharmacological inhibition of class IIa HDACs and direct neuronal overexpression..."** in order to more accurately reflect the pharmacological specificity of TMP269 (**see page 4, line 103**). We have also carefully checked the manuscript for similar wording elsewhere and revised it where necessary to maintain a clear distinction between class IIa HDAC inhibition and HDAC9-specific regulation.

Issue 4: *In the Results section, the sentence "To evaluate the therapeutic potential of a KD on for core autism-related behaviors" (line 98) contains a clear language error and should be corrected.*

Response: We sincerely thank you for identifying this language error. In the revised manuscript, we have corrected the sentence by removing the extraneous word "for". This revision resolves the grammatical problem in the Results section (**see page 4, line 110**).

Issue 5: *The language describing behavioral effects in the Results section should be moderated. Statements such as "KD administration completely reversed these deficits" (line 105) and "short-term BHB treatment was sufficient to fully recapitulate the prosocial effects of the chronic KD" (line 146) appear overstated unless formal equivalence testing was performed.*

Response: We sincerely thank you for this important suggestion. We agree that some of the wording in the Results section was too strong in the absence of formal equivalence testing. In the revised manuscript, we have moderated the relevant language throughout the manuscript to avoid implying full normalization beyond what is directly supported by the data. Specifically, we replaced "completely reversed" with "significantly ameliorated" (**see page 4, line 118**) and "fully recapitulated" with "produced comparable prosocial effects" (**see page 5, line 160**). We also carefully reviewed the surrounding text to ensure that the behavioral effects are described in a more appropriately restrained manner throughout the Results and Discussion.

Issue 6: *The sentence "western blot analysis of ACC tissue reversed that HDAC9 overexpression significantly downregulated..." (line 208) is clearly incorrect and should be revised.*

Response: We sincerely thank you for identifying this typographical error. In the revised manuscript, we have corrected the sentence by replacing "reversed" with "revealed", which accurately reflects the intended meaning (**see page 8, line 231**). This correction has been highlighted in red in the revised manuscript for your convenience.

Issue 7: *In the Discussion, the statement that "HDAC9's role appears both necessary and sufficient..." (line 306) is not fully supported by the current data. Evidence for necessity would require knockdown, knockout, or rescue experiments.*

Response: We sincerely thank you for this important comment. In the revised manuscript, we have revised the original sentence to read **"Our overexpression experiments indicate that HDAC9 upregulation is sufficient to induce synaptic and behavioral phenotypes in this**

model" (**see page 11, lines 336-337**), which states only that HDAC9 upregulation is sufficient to induce the phenotypes. This revised wording removes the unsupported claim of necessity and more accurately reflects the scope of the data presented in the current study.

Issue 8: *The wording regarding TMP269 should be more precise throughout the manuscript. Although TMP269 inhibits class IIa HDACs, the text occasionally implies HDAC9-specific pharmacology. The distinction between class IIa HDAC inhibition and HDAC9-specific regulation should be maintained.*

Response: We sincerely thank you for this important suggestion. We agree that the wording regarding TMP269 should be more precise throughout the manuscript. In response, we have carefully reviewed all references to TMP269 and revised the corresponding text to accurately reflect its pharmacological profile. Specifically, TMP269 is now described consistently as a class IIa HDAC inhibitor, and we have removed or revised wording that could be interpreted as implying HDAC9-specific pharmacology. We have also clarified, where relevant, that the pharmacological experiments support the involvement of the class IIa HDAC pathway **(see page 4, line 103, and page 10, line 320-325)**, whereas the more specific implication of HDAC9 is derived from our molecular expression and viral overexpression data. We believe these revisions now maintain a clearer distinction between class IIa HDAC inhibition and HDAC9-specific regulation throughout the manuscript.

Issue 9: *The Methods section should clarify how sex was considered as a biological variable. Please specify whether males and females were analyzed separately, balanced across groups, or pooled, and provide justification for the chosen approach.*

Response: We sincerely thank you for this important comment. We agree that the treatment of sex as a biological variable should be described more clearly. In the revised Methods, we now explicitly state that both male and female mice were used and were balanced across experimental groups whenever applicable. We also clarify that males and females were pooled for analysis and that sex was not included as an independent factor in the statistical analysis, because the present study was not designed or powered for sex-stratified comparisons. These

revisions have been incorporated into the Mice subsection of the Methods ((**see page 14, lines 428-433**). We believe this revision improves the transparency of the experimental design.

Issue 10: *For Figure 4, the legend should describe in greater detail how CaMKII colocalization was quantified to support the localization claim.*

Response: We sincerely thank you for this helpful suggestion. In the revised manuscript, we have revised the Figure 4 legend to clarify that HDAC9 fluorescence intensity was quantified in CaMKII-positive neurons within the ACC (**see page 26, lines 864-865**). The revised description has been added to better support the localization claim and to improve the transparency of the image analysis.

Issue 11: *The statistical analysis requires clarification, particularly for Figures 5-7. Please indicate whether statistical tests were performed on individual cell-level measurements or on mouse-level averages, given the relatively small number of animals but large number of cells analyzed (e.g., mEPSCs, calcium imaging, spine density).*

Response: We sincerely thank you for this important comment. In the revised manuscript, we have clarified in the corresponding **figures 5-7** legends that, for datasets such as mEPSCs, calcium imaging, and spine density, the statistical analyses were performed using cell-level measurements, and that these cells were obtained from two to four mice per group. We now explicitly report both the number of cells analyzed and the number of contributing animals for these datasets, so that the level of sampling and replication is fully transparent. We believe these revisions improve the clarity of the analytical approach and provide a more accurate description of how these data were handled in the present study.

Issue 12: *Several errors are present in the figure legends. In Figure 1, metabolomics panels K-P are listed even though these data appear in Figure 2. Figure 3 contains duplicate panel labels for D. In Figure 7, mEPSC amplitude is labeled as Q, followed immediately by Q-S for calcium imaging. These inconsistencies should be corrected.*

Response: We sincerely thank you for carefully identifying these presentation inconsistencies. We agree that the figure legends required a thorough consistency check. In the revised manuscript, we have carefully reviewed and corrected the figure legends, panel labels, and the

corresponding in-text figure references throughout the manuscript. Specifically, we corrected the misplaced metabolomics panels in the Figure 1 legend, resolved the duplicated panel D labeling in Figure 3, and corrected the inconsistent panel assignments in Figure 7. We also performed a full cross-check of all figures and legends to ensure consistency between the Results section and the final figure set.

Issue 13: *For the calcium imaging experiments (Figures 6 and 7), the analysis pipeline should be described in greater detail. Please specify the metrics used (e.g., peak ΔF/F, area under the curve, responder fraction), criteria for cell selection, and confirm that identical criteria were applied across all experimental groups.*

Response: We sincerely thank you for this valuable suggestion to improve the transparency of our calcium imaging analysis. In the revised manuscript, we have expanded the In vitro calcium imaging subsection of the Methods to provide additional detail regarding the analysis procedure. Specifically, we now state that the normalized fluorescence signal was quantified as $\Delta F/F = (F - F_0)/F_0$, where F_0 represents the baseline fluorescence intensity before KCl application, and that the peak ΔF/F response after KCl stimulation was used for statistical analysis. We have also clarified that the same imaging settings, cell-selection criteria, and analysis procedures were applied across all experimental groups (**see page 17, lines 542-544**). We believe these revisions improve the transparency and reproducibility of the calcium imaging analysis.

Issue 14: *Figure legends should be further revised to correct typographical errors (e.g., "Scal bar"), awkward wording such as "mouse movement path diagram," and inaccurate descriptions such as "center time/distance measures" in Figure 7.*

Response: We sincerely thank you for identifying these issues in the figure legends. In the revised manuscript, we have corrected typographical errors such as “Scal bar” to “Scale bar”, replaced awkward wording such as “mouse movement path diagram” with “representative movement trace of mice”, and revised imprecise descriptions such as “center time” to the more accurate term “center zone dwell time” where appropriate, including in Figure 7. We have also carefully rechecked the figure legends more broadly to improve consistency in wording and presentation.

Issue 15: *The Key Resources table should be revised to remove duplicate entries (e.g., Rabbit anti-GFP antibody and BCA kits) to improve clarity and accuracy.*

Response: We sincerely thank you for identifying this issue. In the revised manuscript, we have removed duplicate entries, including the repeated Rabbit anti-GFP antibody and BCA kit entries, corrected inconsistencies in catalog numbers and source information, and standardized the formatting throughout the table. We have also carefully rechecked the revised table to ensure consistency with the corresponding descriptions in the Methods section. We believe these revisions improve the clarity and accuracy of the resource information provided in the manuscript.

Issue 16: *The discussion of translational relevance should be more cautious. Statements positioning BHB as a therapeutic candidate for ASD should be tempered, given that the conclusions rely on data from a single mouse model.*

Response: We sincerely thank you for this important comment. In response, we have revised the relevant Discussion paragraphs to moderate statements that previously positioned BHB too broadly as a therapeutic candidate for ASD. The revised text now states more cautiously that our findings suggest BHB may have therapeutic potential for ASD-related social deficits, particularly in contexts involving synaptic and epigenetic dysfunction, without implying broad clinical applicability across the ASD spectrum. We believe this revised framing is better aligned with the actual scope of the data. These modifications were made in the Discussion at **lines 290–293 at page 9, lines 316-318 at page 10, and lines 418–421 at page 13** of the revised manuscript.

References

Newman JC, Verdin E (2017) beta-Hydroxybutyrate: A Signaling Metabolite. Annu Rev Nutr 37: 51-76.

Zhu H, Bi D, Zhang Y, Kong C, Du J, Wu X, Wei Q, Qin H (2022) Ketogenic diet for human diseases: the underlying mechanisms and potential for clinical implementations. Signal transduction and targeted therapy 7: 11.

Apps MA, Rushworth MF, Chang SW (2016) The Anterior Cingulate Gyrus and Social Cognition: Tracking the Motivation of Others. *Neuron* 90: 692-707.

Balsters JH, Apps MA, Bolis D, Lehner R, Gallagher L, Wenderoth N (2017) Disrupted prediction errors index social deficits in autism spectrum disorder. *Brain : a journal of neurology* 140: 235-246.

Guo B, Chen J, Chen Q, Ren K, Feng D, Mao H, Yao H, Yang J, Liu H, Liu Y *et al* (2019) Anterior cingulate cortex dysfunction underlies social deficits in Shank3 mutant mice. *Nat Neurosci* 22: 1223-1234.

Sato M, Nakai N, Fujima S, Choe KY, Takumi T (2023) Social circuits and their dysfunction in autism spectrum disorder. Mol Psychiatry 28: 3194-3206.

Urbain CM, Pang EW, Taylor MJ (2015) Atypical spatiotemporal signatures of working memory brain processes in autism. *Translational psychiatry* 5: e617.

Willbrand EH, Martinez E, Ludwig JJ, Maboudian SA, Weiner KS (2026) Anterior cingulate folding pattern is altered in autism spectrum disorder. *Cerebral cortex (New York, NY : 1991)* 36.

1st Jul 2026

Dear Wenting,

Thank you for sending us your revised manuscript. We have now heard back from the original Referee #1 who was asked to re-evaluate your study. The referee is now satisfied with the modifications made and considers the study as suitable for publication. Before we can formally accept your manuscript, we would ask you to address the following editorial-level issues:

1. Please include individual panel callouts for the EV figures in the manuscript text.

2. Data availability: please remove the reviewer link and provide a resolvable and specific URL for dataset MTBLS1438, and make sure it is publicly available upon the acceptance of the manuscript.

3. Remove highlights from manuscript text.

4. Please note that source data for main figures should be uploaded as one (zipped) file /figure, and named as "manuscriptID_SourceDataForFigure x". Source data for supplementary figures can be combined in one file.

5. Please provide "The paper explained" in the manuscript file. EMBO Molecular Medicine articles are accompanied by a summary of the articles to emphasize the major findings in the paper and their medical implications for the non-specialist reader. Please provide a draft summary of your article highlighting

- the medical issue you are addressing,

- the results obtained and

- their clinical impact.

This may be edited to ensure that readers understand the significance and context of the research. Please refer to any of our published articles for an example.

6. Remove legends for Tables EV1 and EV2 from the manuscript text and add them directly to the corresponding tables. Please correct the citation in the text to Table EV1.

7. The resolution of the submitted EV figures is too low for blot and microscopy images. This reduction in resolution is commonly caused by converting original 16-bit TIFF files to RGB format for publication. While this is not inherently problematic, it can raise concerns about image integrity for critical readers.
To avoid any misunderstanding and to meet EMBO Press standards, we kindly ask that you to resubmit the complete EV Figure set at the captured original data resolution.

8. Please address the following issues in figure legends:
- Please note that the exact p values are not provided in the legends of 1D-F,H-J; 2E,G; 3C-H,J; 4B,L,M,O,Q,R; 5D,E,G-I,L,O,Q,S; 6C,E,F,I,J,M,O,Q; 7D-F,J,L,M,O,P,S; EV-1D; EV-3B; EV-4B,C.
- Please note that information related to n is missing in the legends EV-4C.

I look forward to seeing a revised form of your manuscript as soon as possible.

Kind regards,
Jingyi

Jingyi Hou
Senior Editor
EMBO Molecular Medicine

*** Instructions to submit your revised manuscript ***

*** PLEASE NOTE *** As part of the EMBO Publications transparent editorial process initiative (see our Editorial at

https://www.embopress.org/doi/pdf/10.1002/emmm.201000094), EMBO Molecular Medicine will publish online a Review Process File to accompany accepted manuscripts.

In the event of acceptance, this file will be published in conjunction with your paper and will include the anonymous referee reports, your point-by-point response and all pertinent correspondence relating to the manuscript. If you do NOT want this file to be published, please inform the editorial office at contact@embomolmed.org.

The authors addressed the remaining editorial issues.

8th Jul 2026

Dear Wenting,

We are pleased to inform you that your manuscript is accepted for publication and is now being sent to our publisher to be included in the next available issue of EMBO Molecular Medicine.

Your manuscript will be processed for publication by EMBO Press. It will be copy edited and you will receive page proofs prior to publication. Please note that you will be contacted by Springer Nature Author Services to complete licensing and payment information.

You may qualify for financial assistance for your publication charges - either via a Springer Nature fully open access agreement or an EMBO initiative. Check your eligibility: https://link.springer.com/journal/44321/how-to-publish-with-us

Should you be planning a Press Release on your article, please notify the production team via this form in order to coordinate publication and release dates: https://forms.cloud.microsoft/e/eH4A8JVP6g

If you have any questions, please do not hesitate to contact the Editorial Office. Thank you for your contribution to EMBO Molecular Medicine.

Kind regards,
Jingyi

Jingyi Hou
Senior Editor
EMBO Molecular Medicine

>>> Please note that it is EMBO Molecular Medicine policy for the transcript of the editorial process (containing referee reports and your response letter) to be published as an online supplement to each paper. If you do NOT want this, you will need to inform the Editorial Office via email immediately. More information is available here: https://link.springer.com/partners/embo-press/editorial-policies#Peer%20review

EMBO Press Author Checklist

Corresponding Author Name: Wenting Wang	
Journal Submitted to: EMBO Molecular Medcine	
Manuscript Number: EMM-2026-23556	

USEFUL LINKS FOR COMPLETING THIS FORM
The EMBO Journal - Author Guidelines
EMBO Reports - Author Guidelines
Molecular Systems Biology - Author Guidelines
EMBO Molecular Medicine - Author Guidelines

Reporting Checklist for Life Science Articles (updated January
This checklist is adapted from Materials Design Analysis Reporting (MDAR) Checklist for Authors. MDAR establishes a minimum set of requirements in transparent reporting in the life sciences (see Statement of Task: 10.31222/osf.io/9sm4x). Please follow the journal's guidelines in preparing your
Please note that a copy of this checklist will be published alongside your article.

Abridged guidelines for figures
1. Data
The data shown in figures should satisfy the following conditions:
- the data were obtained and processed according to the field's best practice and are presented to reflect the results of the experiments in an accurate and unbiased manner.
- ideally, figure panels should include only measurements that are directly comparable to each other and obtained with the same assay.
- plots include clearly labeled error bars for independent experiments and sample sizes. Unless justified, error bars should not be shown for technical
- if n<5, the individual data points from each experiment should be plotted. Any statistical test employed should be justified.
- Source Data should be included to report the data underlying figures according to the guidelines set out in the authorship guidelines on Data

2. Captions
Each figure caption should contain the following information, for each panel where they are relevant:
- a specification of the experimental system investigated (eg cell line, species name).
- the assay(s) and method(s) used to carry out the reported observations and measurements.
- an explicit mention of the biological and chemical entity(ies) that are being measured.
- an explicit mention of the biological and chemical entity(ies) that are altered/varied/perturbed in a controlled manner.
- the exact sample size (n) for each experimental group/condition, given as a number, not a range;
- a description of the sample collection allowing the reader to understand whether the samples represent technical or biological replicates (including how many animals, litters, cultures, etc.).
- a statement of how many times the experiment shown was independently replicated in the laboratory.
- definitions of statistical methods and measures:
  - common tests, such as t-test (please specify whether paired vs. unpaired), simple χ2 tests, Wilcoxon and Mann-Whitney tests, can be unambiguously identified by name only, but more complex techniques should be described in the methods section;
  - are tests one-sided or two-sided?
  - are there adjustments for multiple comparisons?
  - exact statistical test results, e.g., P values = x but not P values < x;
  - definition of 'center values' as median or average;
  - definition of error bars as s.d. or s.e.m.

Please complete ALL of the questions below.
Select "Not Applicable" only when the requested information is not relevant for your study.

Materials

Newly Created Materials	Information included in the manuscript?	In which section is the information available? (Reagents and Tools Table, Materials and Methods, Figures, Data Availability Section)
New materials and reagents need to be available; do any restrictions apply?	Not Applicable	

Antibodies	Information included in the manuscript?	In which section is the information available? (Reagents and Tools Table, Materials and Methods, Figures, Data Availability Section)
For **antibodies** provide the following information:<br>- Commercial antibodies: RRID (if possible) or supplier name, catalogue number and or/clone number<br>- Non-commercial: RRID or citation	Yes	Reagents and Tools Tables

DNA and RNA sequences	Information included in the manuscript?	In which section is the information available? (Reagents and Tools Table, Materials and Methods, Figures, Data Availability Section)
**Short novel DNA or RNA including primers, probes**: provide the sequences.	Yes	Materials and Methods

Cell materials	Information included in the manuscript?	In which section is the information available? (Reagents and Tools Table, Materials and Methods, Figures, Data Availability Section)
**Cell lines:** Provide species information, strain. Provide accession number in repository **OR** supplier name, catalog number, clone number, and/**OR** RRID.	Not Applicable	
**Primary cultures:** Provide species, strain, sex of origin, genetic modification status.	Yes	Materials and Methods
Report if the cell lines were recently **authenticated** (e.g., by STR profiling) and tested for mycoplasma contamination.	Not Applicable	

Experimental animals	Information included in the manuscript?	In which section is the information available? (Reagents and Tools Table, Materials and Methods, Figures, Data Availability Section)
**Laboratory animals or Model organisms:** Provide species, strain, sex, age, genetic modification status. Provide accession number in repository **OR** supplier name, catalog number, clone number, **OR** RRID.	Yes	Materials and Methods
**Animal observed in or captured from the field:** Provide species, sex, and age where possible.	Not Applicable	
Please detail **housing and husbandry conditions**.	Yes	Materials and Methods

Plants and microbes	Information included in the manuscript?	In which section is the information available? (Reagents and Tools Table, Materials and Methods, Figures, Data Availability Section)
**Plants:** provide species and strain, ecotype and cultivar where relevant, unique accession number if available, and source (including location for collected wild specimens).	Not Applicable	
**Microbes:** provide species and strain, unique accession number if available, and source.	Not Applicable	

Human research participants	Information included in the manuscript?	In which section is the information available? (Reagents and Tools Table, Materials and Methods, Figures, Data Availability Section)
If collected and within the bounds of privacy constraints report on age, sex and gender or ethnicity for all study participants.	Not Applicable	

Core facilities	Information included in the manuscript?	In which section is the information available? (Reagents and Tools Table, Materials and Methods, Figures, Data Availability Section)
If your work benefited from core facilities, was their service mentioned in the acknowledgments section?	Not Applicable	

Design

Study protocol	Information included in the manuscript?	In which section is the information available? (Reagents and Tools Table, Materials and Methods, Figures, Data Availability Section)
If study protocol has been **pre-registered, provide DOI in the manuscript**. For clinical trials, provide the trial registration number **OR** cite DOI.	Not Applicable	
Report the **clinical trial registration number** (at ClinicalTrials.gov or equivalent), where applicable.	Not Applicable	

Laboratory protocol	Information included in the manuscript?	In which section is the information available? (Reagents and Tools Table, Materials and Methods, Figures, Data Availability Section)
Provide DOI OR other citation details if **external detailed step-by-step protocols** are available.	Not Applicable	

Experimental study design and statistics	Information included in the manuscript?	In which section is the information available? (Reagents and Tools Table, Materials and Methods, Figures, Data Availability Section)
Include a statement about **sample size** estimate even if no statistical methods were used.	Yes	Figures
Were any steps taken to minimize the effects of subjective bias when allocating animals/samples to treatment (e.g. **randomization procedure**)? If yes, have they been described?	Not Applicable	
Include a statement about **blinding** even if no blinding was done.	Yes	Material and Methods
Describe **inclusion/exclusion criteria** if samples or animals were excluded from the analysis. Were the criteria pre-established?<br><br>If sample or data points were omitted from analysis, report if this was due to attrition or intentional exclusion and provide justification.	Not Applicable	
For every figure, are **statistical tests** justified as appropriate? Do the data meet the assumptions of the tests (e.g., normal distribution)? Describe any methods used to assess it. Is there an estimate of variation within each group of data? Is the variance similar between the groups that are being statistically compared?	Yes	Figures

Sample definition and in-laboratory replication	Information included in the manuscript?	In which section is the information available? (Reagents and Tools Table, Materials and Methods, Figures, Data Availability Section)
In the figure legends: state number of times the experiment was **replicated** in laboratory.	Yes	Figures
In the figure legends: define whether data describe **technical or biological replicates**.	Yes	Figures

Ethics

Ethics	Information included in the manuscript?	In which section is the information available? (Reagents and Tools Table, Materials and Methods, Figures, Data Availability Section)
Studies involving **human participants**: State details of **authority granting ethics approval** (IRB or equivalent committee(s), provide reference number for approval.	Not Applicable	
Studies involving **human participants**: Include a statement confirming that **informed consent** was obtained from all subjects and that the experiments conformed to the principles set out in the WMA Declaration of Helsinki and the Department of Health and Human Services Belmont Report.	Not Applicable	
Studies involving **human participants: For** publication of **patient photos**, include a statement confirming that consent to publish was obtained.	Not Applicable	
Studies involving experimental **animals**: State details of **authority granting ethics approval** (IRB or equivalent committee(s), provide reference number for approval. Include a statement of compliance with ethical regulations.	Yes	Materials and Methods
Studies involving **specimen and field samples:** State if relevant **permits** obtained, provide details of authority approving study; if none were required, explain why.	Not Applicable	

Dual Use Research of Concern (DURC)	Information included in the manuscript?	In which section is the information available? (Reagents and Tools Table, Materials and Methods, Figures, Data Availability Section)
Could your study fall under dual use research restrictions? Please check biosecurity documents and list of **select agents and toxins** (CDC): https://www.selectagents.gov/sat/list.htm	Not Applicable	
If you used a select agent, is the security level of the lab appropriate and reported in the manuscript?	Not Applicable	
If a study is subject to dual use research of concern regulations, is the name of the **authority granting approval and reference number** for the regulatory approval provided in the manuscript?	Not Applicable	

Reporting

The MDAR framework recommends adoption of discipline-specific guidelines, established and endorsed through community initiatives. Journals have their own policy about requiring specific guidelines and recommendations to complement MDAR.

Adherence to community standards	Information included in the manuscript?	In which section is the information available? (Reagents and Tools Table, Materials and Methods, Figures, Data Availability Section)
State if relevant guidelines or checklists (e.g., **ICMJE, MIBBI, ARRIVE, PRISMA**) have been followed or provided.	Not Applicable	
For **tumor marker prognostic studies,** we recommend that you follow the **REMARK** reporting guidelines (see link list at top right). See author guidelines, under 'Reporting Guidelines'. Please confirm you have followed these guidelines.	Not Applicable	
For **phase II and III randomized controlled trials**, please refer to the **CONSORT** flow diagram (see link list at top right) and submit the CONSORT checklist (see link list at top right) with your submission. See author guidelines, under 'Reporting Guidelines'. Please confirm you have submitted this list.	Not Applicable	

Data Availability

Data availability	Information included in the manuscript?	In which section is the information available? (Reagents and Tools Table, Materials and Methods, Figures, Data Availability Section)
Have **primary datasets** been deposited according to the journal's guidelines (see 'Data Deposition' section) and the respective accession numbers provided in the Data Availability Section?	Yes	Data Avaliablity Section
Were **human clinical and genomic datasets** deposited in a public access-controlled repository in accordance to ethical obligations to the patients and to the applicable consent agreement?	Not Applicable	
Are **computational models** that are central and integral to a study available without restrictions in a machine-readable form? Were the relevant accession numbers or links provided?	Not Applicable	
If publicly available data were reused, provide the respective **data citations in the reference list**.	Not Applicable	
