# Supplementary material for: β-hydroxybutyrate restores social deficits by suppressing HDAC9 in the ACC of Shank3B-deficient mice
Source: EMBO Mol Med. 2026 Jul 21;18(9):3517–39. doi: 10.1038/s44321-026-00491-9 (PMC13562639; doi:10.1038/s44321-026-00491-9)
Supplement: Supplementary file 3 [file 44321_2026_491_MOESM1_ESM.docx]

Table EV1. The energy composition of ketogenic and control diet.

| **Items** | **XTKDCON** | **XTKD01** |
| --- | --- | --- |
|  | Energy (kcal) % | Energy (kcal) % |
| Proteins | 9.99 | 9.99 |
| Fat | 9.97 | 0 |
| Carbohydrate | 79.9 | 89.91 |
| **Ingredient** | **Energy (kcal)** | **Energy (kcal)** |
| Casein | 400.00 | 400.00 |
| L-Cystine | 6.00 | 6.00 |
| Corn Starch | 1484.00 | 0.00 |
| Maltodextrin | 140.00 | 0.00 |
| Sucrose | 1624.00 | 0.00 |
| Celluose | 0.00 | 0.00 |
| Soybean oil | 225.00 | 225.00 |
| Cocoa Butter | 180.00 | 3429.00 |
| Mineral Mix | 0.00 | 0.00 |
| Vitamin Mix | 4.00 | 4.00 |
| FD&C Blue Dye | 0.00 | 0.00 |
| FD&C Yellow Dye | 0.00 | 0.00 |
| Choline | 0.00 | 0.00 |
| **Total** | **4063.00** | **4064.00** |

Provided by Xietong Pharmaceutical Bio-Engineering Co., Ltd.
