# Supplementary material for: β-hydroxybutyrate restores social deficits by suppressing HDAC9 in the ACC of Shank3B-deficient mice
Source: EMBO Mol Med. 2026 Jul 21;18(9):3517–39. doi: 10.1038/s44321-026-00491-9 (PMC13562639; doi:10.1038/s44321-026-00491-9)
Supplement: Supplementary file 4 [file 44321_2026_491_MOESM2_ESM.docx]

Table EV2. Statistical summary of all quantified data presented in the figures.

| **Data** | **Response variable** | **Groups** | **n define as** | **slice/**  **animal used** | **Normality Test (Shapiro-Wilk)** | **Homogeneity of variance test (Brown-Forsythe’s test)** | **Hypothesis test** | **Test value** | **P value** | **summary** |
| --- | --- | --- | --- | --- | --- | --- | --- | --- | --- | --- |
|  |  |  |  |  |  |  |  |  |  |  |
| **Fig 1B** | **Weight change (g)** | WT | Mouse | 6 mice | W=0.9465 df=5 p=0.6944 |  | Two-way RM ANOVA | F(6,40)=1.134 | P ≥ 0.05 | n.s |
|  |  | KO+SD |  | 6 mice | W=0.9515 df=5 p=0.7257 |  |  |  |  |  |
|  |  | KO+KD |  | 6 mice | W=0.9120 df=5 p=0.4933 |  |  |  |  |  |
| **Fig 1D** | **Time in compartment (s)** | WT (S) | mouse | 6 mice | W=0.9771 df=5 p=0.9362 | F=1.516 df1=5 df2=5 p=0.6592 | Two-tailed unpaired t-test | t=-7.320 df=10 | P ≤ 0.0001 | **** |
|  |  | WT (E) |  | 6 mice | W=0.9229 df=5 p=0.5262 |  |  |  |  |  |
|  |  | KO+SD (S) | mouse | 6 mice | W=0.8812 df=5 p=0.2746 | F=2.373 df1=5 df2=5 p=0.3647 | Two-tailed unpaired t-test | t=-5.501 df=10 | P = 0.5945 | n.s |
|  |  | KO+SD (E) |  |  | W=0.9950 df=5 p=0.9979 |  |  |  |  |  |
|  |  | KO+KD (S) | mouse | 6 mice | W=0.9495 df=5 p=0.7365 | F=1.608 df1=5 df2=5 p=0.6149 | Two-tailed unpaired t-test | t=-4.131 df=10 | P = 0.0020 | ** |
|  |  | KO+KD (E) |  |  | W=0.9659 df=5 p=0.8638 |  |  |  |  |  |
| **Fig 1E** | **Preference Score** | WT | mouse | 6 mice | W=0.9180 df=5 p=0.4911 | F=0.5936 df1=2 df2=15 p=0.5648 | Repeated measurement ANOVA | F(2,15)=4.381 | P ≤ 0.05 | * |
|  |  | KO+SD |  | 6 mice | W=0.9137 df=5 p=0.4610 |  |  |  |  |  |
|  |  | KO+KD |  | 6 mice | W=0.9580 df=5 p=0.8039 |  |  |  |  |  |
|  |  | KO+SD vs. WT |  |  |  |  | Tukey's multiple comparisons test |  | P = 0.0327 | * |
|  |  | KO+SD vs. KO+KD |  |  |  |  | Tukey's multiple comparisons test |  | P = 0.0653 | n.s |
|  |  | WT vs. KO+KD |  |  |  |  | Tukey's multiple comparisons test |  | P = 0.9771 | n.s |
| **Fig 1F** | **Social interaction time (s)** | WT | mouse | 6 mice | W=0.8937 df=5 p=0.3380 | F=0.5237 df1=2 df2=15 p=0.6027 | Repeated measurement ANOVA | F(2,15)=6.171 | P ≤ 0.05 | * |
|  |  | KO+SD |  | 6 mice | W=0.9176 df=5 p=0.4882 |  |  |  |  |  |
|  |  | KO+KD |  | 6 mice | W=0.8730 df=5 p=0.2386 |  |  |  |  |  |
|  |  | KO+SD vs. WT |  |  |  |  | Tukey's multiple comparisons test |  | P = 0.0342 | * |
|  |  | KO+SD vs. KO+KD |  |  |  |  | Tukey's multiple comparisons test |  | P = 0.0143 | * |
|  |  | WT vs. KO+KD |  |  |  |  | Tukey's multiple comparisons test |  | P = 0.8974 | n.s |
| **Fig 1H** | **Time in center zone (s)** | WT | mouse | 6 mice | W=0.9834 df=5 p=0.9671 | F=0.7638 df1=2 df2=15 p=0.4832 | Repeated measurement ANOVA | F(2,15)=5.518 | P ≤ 0.05 | * |
|  |  | KO+SD |  | 6 mice | W=0.8909 df=5 p=0.3227 |  |  |  |  |  |
|  |  | KO+KD |  | 6 mice | W=0.8129 df=5 p=0.0765 |  |  |  |  |  |
|  |  | KO+SD vs. WT |  |  |  |  | Tukey's multiple comparisons test |  | P = 0.0189 | * |
|  |  | KO+SD vs. KO+KD |  |  |  |  | Tukey's multiple comparisons test |  | P = 0.0513 | n.s |
|  |  | WT vs. KO+KD |  |  |  |  | Tukey's multiple comparisons test |  | P = 0.8645 | n.s |
| **Fig 1I** | **Total distance (cm)** | WT | mouse | 6 mice | W=0.9159 df=5 p=0.4761 | F=1.986 df1=2 df2=15 p=0.1718 | Repeated measurement ANOVA | F(2,15)=7.396 | P ≤ 0.01 | ** |
|  |  | KO+SD |  | 6 mice | W=0.8761 df=5 p=0.2515 |  |  |  |  |  |
|  |  | KO+KD |  | 6 mice | W=0.9518 df=5 p=0.7552 |  |  |  |  |  |
|  |  | KO+SD vs. WT |  |  |  |  | Tukey's multiple comparisons test |  | P ≤ 0.01 | ** |
|  |  | KO+SD vs. KO+KD |  |  |  |  | Tukey's multiple comparisons test |  | P = 0.1848 | ns |
|  |  | WT vs. KO+KD |  |  |  |  | Tukey's multiple comparisons test |  | P = 0.1499 | n.s |
| **Fig 1J** | **Grooming duration (%)** | WT | mouse | 6 mice | Not all subgroups meets p＞0.05 |  | Kruskal-Wallis test | H=-8.082 df=15 | P ≤ 0.05 | * |
|  |  | KO+SD |  | 6 mice |  |  |  |  |  |  |
|  |  | KO+KD |  | 6 mice |  |  |  |  |  |  |
|  |  | KO+SD vs. WT |  |  |  |  | Tukey's multiple comparisons test |  | P = 0.0161 | * |
|  |  | KO+SD vs. KO+KD |  |  |  |  | Tukey's multiple comparisons test |  | P＞ 0.9999 | ns |
| **Fig 2E** | **AcAc (μg/mL)** | KO+SD | mouse | 4 mice | W=0.9683 df=3 p=0.8306 | F=6.172 df1=3 df2=3 p=0.1692 | Two-tailed unpaired t-test | t=4.753 df=6 | P = 0.0031 | ** |
|  |  | KO+KD |  | 4 mice | W=0.9923 df=3 p=0.9688 |  |  |  |  |  |
| **Fig 2G** | **BHB (μg/mL)** | KO+SD | mouse | 4 mice | W=0.9807 df=3 p=0.9091 | F=102.1 df1=3 df2=3 p=0.032 | Mann-Whitney U test | U=0 | P = 0.0286 | * |
|  |  | KO+KD |  | 4 mice | W=0.8799 df=3 p=0.3381 |  |  |  |  |  |
| **Fig 3C** | **Time in compartment (s)** | WT (S) | mouse | 5 mice | W=0.9154 df=5 p=0.5005 | F=1.786 df1=4 df2=4 p=0.5882 | Two-tailed unpaired t-test | t=-15.87 df=8 | P ≤ 0.0001 | **** |
|  |  | WT (E) |  | 5 mice | W=0.9112 df=5 p=0.4748 |  |  |  |  |  |
|  |  | KO+saline (S) | mouse | 10 mice | W=0.9317 df=9 p=0.4648 | F=1.375 df1=9 df2=9 p=0.6432 | Two-tailed unpaired t-test | t=1.303 df=18 | P = 0.2090 | ns |
|  |  | KO+ saline (E) |  | 10 mice | W=0.9578 df=9 p=0.7606 |  |  |  |  |  |
|  |  | KO+BHB (S) | mouse | 10 mice | W=0.9457 df=9 p=0.6180 | F=1.527 df1=9 df2=9 p=0.5380 | Two-tailed unpaired t-test | t=-3.270 df=18 | P = 0.0043 | ** |
|  |  | KO+BHB (E) |  | 10 mice | W=0.9579 df=9 p=0.7613 |  |  |  |  |  |
| **Fig 3D** | **Preference Score** | WT | mouse | 5 mice | W=0.8209 df=4 p=0.1187 | F=1.603 df1=2 df2=22 p=0.2240 | Repeated measurement ANOVA | F(2,22)=6.674 | P ≤ 0.00 | ** |
|  |  | KO+saline |  | 10 mice | W=0.8469 df=9 p=0.0533 |  |  |  |  |  |
|  |  | KO+BHB |  | 10 mice | W=0.9009 df=9 p=0.2239 |  |  |  |  |  |
|  |  | KO+saline vs. WT |  |  |  |  | Tukey's multiple comparisons test |  | P = 0.0069 | ** |
|  |  | KO+ saline vs. KO+BHB |  |  |  |  | Tukey's multiple comparisons test |  | P = 0.0417 | * |
|  |  | WT vs. KO+BHB |  |  |  |  | Tukey's multiple comparisons test |  | P = 0.4193 | ns |
| **Fig 3E** | **Social interaction time (s)** | WT | mouse | 5 mice | W=0.9761 df=4 p=0.9129 | F=3.381 df1=2 df2=22 p=0.0525 | Repeated measurement ANOVA | F(2,22)=5.988 | P ≤ 0.05 | * |
|  |  | KO+saline |  | 10 mice | W=0.9567 df=9 p=0.7473 |  |  |  |  |  |
|  |  | KO+BHB |  | 10 mice | W=0.9667 df=9 p=0.8586 |  |  |  |  |  |
|  |  | KO+saline vs. WT |  |  |  |  | Tukey's multiple comparisons test |  | P = 0.0097 | ** |
|  |  | KO+ saline vs. KO+BHB |  |  |  |  | Tukey's multiple comparisons test |  | P = 0.0634 | ns |
|  |  | WT vs. KO+BHB |  |  |  |  | Tukey's multiple comparisons test |  | P = 0.4092 | ns |
| **Fig 3G** | **Time in center zone (s)** | WT | mouse | 5 mice | W=0.8794 df=4 p=0.3065 | F=2.357 df1=2 df2=22 p=0.1182 | Repeated measurement ANOVA | F(2,22)=14.39 | P ≤ 0.001 | *** |
|  |  | KO+saline |  | 10 mice | W=0.9326 df=9 p=0.4738 |  |  |  |  |  |
|  |  | KO+BHB |  | 10 mice | W=0.8840 df=9 p=0.1449 |  |  |  |  |  |
|  |  | KO+saline vs. WT |  |  |  |  | Tukey's multiple comparisons test |  | P ≤ 0.0001 | **** |
|  |  | KO+ saline vs. KO+BHB |  |  |  |  | Tukey's multiple comparisons test |  | P = 0.0547 | ns |
|  |  | WT vs. KO+BHB |  |  |  |  | Tukey's multiple comparisons test |  | P = 0.0080 | *** |
| **Fig 3H** | **Total distance (cm)** | WT | mouse | 5 mice | W=0.9738 df=4 p=0.8993 | F=1.767 df1=2 df2=22 p=0.1942 | Repeated measurement ANOVA | F(2,22)=22.16 | P ≤ 0.0001 | **** |
|  |  | KO+saline |  | 10 mice | W=0.9214 df=9 p=0.3690 |  |  |  |  |  |
|  |  | KO+BHB |  | 10 mice | W=0.9101 df=9 p=0.2814 |  |  |  |  |  |
|  |  | KO+saline vs. WT |  |  |  |  | Tukey's multiple comparisons test |  | P ≤ 0.0001 | **** |
|  |  | KO+ saline vs. KO+BHB |  |  |  |  | Tukey's multiple comparisons test |  | P = 0.2789 | ns |
|  |  | WT vs. KO+BHB |  |  |  |  | Tukey's multiple comparisons test |  | P ≤ 0.0001 | **** |
| **Fig 3J** | **Relative no. of c-FOS (%)** | WT | mouse | 3 mice | W=0.9985 df=4 p=0.9265 | F=0.8869 df1=2 df2=6 p=0.4598 | Repeated measurement ANOVA | F(2,6)=20.64 | P ≤ 0.01 | ** |
|  |  | KO+saline |  | 3 mice | W=0.9832 df=9 p=0.7517 |  |  |  |  |  |
|  |  | KO+BHB |  | 3 mice | W=0.9989 df=9 p=0.9370 |  |  |  |  |  |
|  |  | KO+saline vs. WT |  |  |  |  | Tukey's multiple comparisons test |  | P = 0.0426 | * |
|  |  | KO+ saline vs. KO+BHB |  |  |  |  | Tukey's multiple comparisons test |  | P = 0.0016 | ** |
|  |  | WT vs. KO+BHB |  |  |  |  | Tukey's multiple comparisons test |  | P = 0.0412 | * |
| **Fig 4B** | **Relative mRNA levels (ACC)** | WT (Hdac1) | mouse | 3 mice | W=0.9571 df=2 p=0.6014 | F=8.280 df1=2 df2=2 p=0.2155 | Two-tailed unpaired t-test | t=2.481 df=4 | P = 0.0681 | ns |
|  |  | KO (Hdac1) |  | 3 mice | W=0.9912 df=2 p=0.8209 |  |  |  |  |  |
|  |  | WT (Hdac2) | mouse | 3 mice | W=0.9046 df=2 p=0.4004 | F=48.95 df1=2 df2=2 p=0.0400 | Mann-Whitney U test | U=3 | P = 0.7000 | ns |
|  |  | KO (Hdac2) |  | 3 mice | W=0.7866 df=2 p=0.0828 |  |  |  |  |  |
|  |  | WT (Hdac3) | mouse | 3 mice | W=0.9482 df=2 p=0.5614 | F=3.701 df1=2 df2=2 p=0.4254 | Two-tailed unpaired t-test | t=1.145 df=4 | P = 0.4254 | ns |
|  |  | KO (Hdac3) |  | 3 mice | W=0.9634 df=2 p=0.6323 |  |  |  |  |  |
|  |  | WT (Hdac8) | mouse | 3 mice | W=0.9526 df=2 p=0.5809 | F=3.248 df1=2 df2=2 p=0.4708 | Two-tailed unpaired t-test | t=4.105 df=4 | P = 0.0148 | * |
|  |  | KO (Hdac8) |  | 3 mice | W=0.9507 df=2 p=0.5726 |  |  |  |  |  |
|  |  | WT (Hdac4) | mouse | 3 mice | W=0.9739 df=2 p=0.6902 | F=2.722 df1=2 df2=2 p=0.5437 | Two-tailed unpaired t-test | t=1.983 df=4 | P = 0.1184 | ns |
|  |  | KO (Hdac4) |  | 3 mice | W=0.8914 df=2 p=0.3585 |  |  |  |  |  |
|  |  | WT (Hdac5) | mouse | 3 mice | W=0.9322 df=2 p=0.4969 | F=5.866 df1=2 df2=2 p=0.2913 | Two-tailed unpaired t-test | t=3.302 df=4 | P = 0.0299 | * |
|  |  | KO (Hdac5) |  | 3 mice | W=0.9995 df=2 p=0.9591 |  |  |  |  |  |
|  |  | WT (Hdac7) | mouse | 3 mice | W=0.9991 df=2 p=0.9426 | F=4.022 df1=2 df2=2 p=0.3983 | Two-tailed unpaired t-test | t=1.243 df=4 | P = 0.2817 | ns |
|  |  | KO (Hdac7) |  | 3 mice | W=0.9336 df=3 p=0.5002 |  |  |  |  |  |
|  |  | WT (Hdac9) | mouse | 3 mice | W=0.9967 df=3 p=0.8896 | F=2.226 df1=2 df2=2 p=0.6199 | Two-tailed unpaired t-test | t=2.933 df=4 | P = 0.0427 | * |
|  |  | KO (Hdac9) |  | 3 mice | W=0.9738 df=2 p=0.6894 |  |  |  |  |  |
| **Fig 4D** | **Relative mRNA levels (STR)** | WT (Hdac1) | mouse | 3 mice | W=0.9291 df=2 p=0.4852 | F=6.153 df1=2 df2=2 p=0.2796 | Two-tailed unpaired t-test | t=1.211 df=4 | P = 0.2924 | ns |
|  |  | KO (Hdac1) |  | 3 mice | W=0.9300 df=2 p=0.4886 |  |  |  |  |  |
|  |  | WT (Hdac2) | mouse | 3 mice | W=0.9134 df=2 p=0.4294 | F=3.712 df1=2 df2=2 p=0.4244 | Two-tailed unpaired t-test | t=0.6105 df=4 | P = 0.5745 | ns |
|  |  | KO (Hdac2) |  | 3 mice | W=0.9239 df=3 p=0.4662 |  |  |  |  |  |
|  |  | WT (Hdac3) | mouse | 3 mice | W=0.8462 df=3 p=0.2303 | F=1591 df1=2 df2=2 p=0.0013 | Mann-Whitney U test | U=3 | P = 0.7000 | ns |
|  |  | KO (Hdac3) |  | 3 mice | W=0.9741 df=2 p=0.6912 |  |  |  |  |  |
|  |  | WT (Hdac8) | mouse | 3 mice | W=0.9819 df=2 p=0.7422 | F=1.3255 df1=2 df2=2 p=0.8601 | Two-tailed unpaired t-test | t=0.4131 df=4 | P = 0.7007 | ns |
|  |  | KO (Hdac8) |  | 3 mice | W=0.8567 df=2 p=0.2585 |  |  |  |  |  |
|  |  | WT (Hdac4) | mouse | 3 mice | W=0.8183 df=2 p=0.1589 | F=15.14 df1=2 df2=2 p=0.1239 | Two-tailed unpaired t-test | t=0.9780 df=4 | P = 0.3834 | ns |
|  |  | KO (Hdac4) |  | 3 mice | W=0.8705 df=3 p=0.2969 |  |  |  |  |  |
|  |  | WT (Hdac5) | mouse | 3 mice | W=0.9774 df=2 p=0.7119 | F=7.134 df1=2 df2=2 p=0.2459 | Two-tailed unpaired t-test | t=0.9079 df=4 | P = 0.4153 | ns |
|  |  | KO (Hdac5) |  | 3 mice | W=0.8542 df=2 p=0.2517 |  |  |  |  |  |
|  |  | WT (Hdac7) | mouse | 3 mice | W=1.0000 df=2 p=0.9873 | F=12.61 df1=2 df2=2 p=0.1469 | Two-tailed unpaired t-test | t=0.8887 df=4 | P = 0.4244 | ns |
|  |  | KO (Hdac7) |  | 3 mice | W=0.9715 df=2 p=0.6758 |  |  |  |  |  |
|  |  | WT (Hdac9) | mouse | 3 mice | W=0.9031 df=2 p=0.3955 | F=21.31 df1=2 df2=2 p=0.0896 | Two-tailed unpaired t-test | t=0.7384 df=4 | P = 0.5013 | ns |
|  |  | KO (Hdac9) |  | 3 mice | W=0.9082 df=2 p=0.9181 |  |  |  |  |  |
| **Fig 4F** | **Relative mRNA levels (HIP)** | WT (Hdac1) | mouse | 3 mice | W=0.8999 df=2 p=0.3852 | F=4.824 df1=2 df2=2 p=0.3434 | Two-tailed unpaired t-test | t=1.308 df=4 | P = 0.2609 | ns |
|  |  | KO (Hdac1) |  | 3 mice | W=0.8131 df=2 p=0.1461 |  |  |  |  |  |
|  |  | WT (Hdac2) | mouse | 3 mice | W=0.9265 df=2 p=0.4756 | F=3.010 df1=2 df2=2 p=0.4988 | Two-tailed unpaired t-test | t=0.1080 df=4 | P = 0.9192 | ns |
|  |  | KO (Hdac2) |  | 3 mice | W=0.8782 df=3 p=0.3192 |  |  |  |  |  |
|  |  | WT (Hdac3) | mouse | 3 mice | W=0.9381 df=2 p=0.5197 | F=5.726 df1=2 df2=2 p=0.2973 | Two-tailed unpaired t-test | t=0.2391 df=4 | P = 0.8228 | ns |
|  |  | KO (Hdac3) |  | 3 mice | W=0.9470 df=2 p=0.5563 |  |  |  |  |  |
|  |  | WT (Hdac8) | mouse | 3 mice | W=0.9384 df=2 p=0.5209 | F=1.120 df1=2 df2=2 p=0.9432 | Two-tailed unpaired t-test | t=0.4100 df=4 | P = 0.7028 | ns |
|  |  | KO (Hdac8) |  | 3 mice | W=0.7733 df=2 p=0.0522 |  |  |  |  |  |
|  |  | WT (Hdac4) | mouse | 3 mice | W=0.8611 df=2 p=0.2706 | F=1.069 df1=2 df2=2 p=0.9667 | Two-tailed unpaired t-test | t=0.06557 df=4 | P = 0.9509 | ns |
|  |  | KO (Hdac4) |  | 3 mice | W=0.9641 df=2 p=0.6358 |  |  |  |  |  |
|  |  | WT (Hdac5) | mouse | 3 mice | W=0.8837 df=2 p=0.3352 | F=4.237 df1=2 df2=2 p=0.3819 | Two-tailed unpaired t-test | t=1.859 df=4 | P = 0.1366 | ns |
|  |  | KO (Hdac5) |  | 3 mice | W=0.9982 df=2 p=0.9179 |  |  |  |  |  |
|  |  | WT (Hdac7) | mouse | 3 mice | W=0.8477 df=2 p=0.2343 | F=5.095 df1=2 df2=2 p=0.3281 | Two-tailed unpaired t-test | t=0.2018 df=4 | P = 0.8500 | ns |
|  |  | KO (Hdac7) |  | 3 mice | W=0.8501 df=2 p=0.2408 |  |  |  |  |  |
|  |  | WT (Hdac9) | mouse | 3 mice | W=0.8191 df=2 p=0.1609 | F=1.131 df1=2 df2=2 p=0.9384 | Two-tailed unpaired t-test | t=0.1482 df=4 | P = 0.8893 | ns |
|  |  | KO (Hdac9) |  | 3 mice | W=0.9440 df=2 p=0.5437 |  |  |  |  |  |
| **Fig 4H** | **Relative intensity**  **(HDAC5/actin)** | WT (ACC) | mouse | 3 mice | Not all subgroups meets p＞0.05 |  | Mann-Whitney U test | U=0 | P = 0.1000 | ns |
|  |  | KO (ACC) |  | 3 mice |  |  |  |  |  |  |
|  |  | WT (STR) | mouse | 3 mice | Not all subgroups meets p＞0.05 |  | Mann-Whitney U test | U=3 | P = 0.7000 | ns |
|  |  | KO (STR) |  | 3 mice |  |  |  |  |  |  |
|  |  | WT (HIP) | mouse | 3 mice | Not all subgroups meets p＞0.05 |  | Mann-Whitney U test | U=3 | P = 0.7000 | ns |
|  |  | KO (HIP) |  | 3 mice |  |  |  |  |  |  |
| **Fig 4J** | **Relative intensity**  **(HDAC8/actin)** | WT (ACC) | mouse | 3 mice | Not all subgroups meets p＞0.05 |  | Mann-Whitney U test | U=3 | P = 0.7000 | ns |
|  |  | KO (ACC) |  | 3 mice |  |  |  |  |  |  |
|  |  | WT (STR) | mouse | 3 mice | Not all subgroups meets p＞0.05 |  | Mann-Whitney U test | U=3 | P = 0.7000 | ns |
|  |  | KO (STR) |  | 3 mice |  |  |  |  |  |  |
|  |  | WT (HIP) | mouse | 3 mice | Not all subgroups meets p＞0.05 |  | Mann-Whitney U test | U=3 | P = 0.7000 | ns |
|  |  | KO (HIP) |  | 3 mice |  |  |  |  |  |  |
| **Fig 4L** | **Relative intensity**  **(HDAC9/actin)** | WT (ACC) | mouse | 3 mice | W=1.000 df=2 p＞0.9999 | F=13.51 df1=2 df2=2 p=0.1378 | Two-tailed unpaired t-test | t=4.008 df=4 | P = 0.0160 | * |
|  |  | KO (ACC) |  | 3 mice | W=0.8246 df=2 p=0.1746 |  |  |  |  |  |
|  |  | WT (STR) | mouse | 3 mice | W=1.000 df=3 p＞0.9999 | F=67.27 df1=2 df2=2 p=0.0293 | Mann-Whitney U test | U=0 | P = 0.1000 | ns |
|  |  | KO (STR) |  | 3 mice | W=0.8195 df=2 p=0.1619 |  |  |  |  |  |
|  |  | WT (HIP) | mouse | 3 mice | Not all subgroups meets p＞0.05 |  | Mann-Whitney U test | U=0 | P = 0.1000 | ns |
|  |  | KO (HIP) |  | 3 mice |  |  |  |  |  |  |
| **Fig 4M** | **Relative Hdac9 mRNA levels** | WT | groups | 5 groups (12-14 cells per group)/2 mice | W=0.8839 df=4 p=0.3275 | F=3.499 df1=4 df2=5 p=0.2486 | Two-tailed unpaired t-test | t=2.577 df=9 | P = 0.0298 | * |
|  |  | KO |  | 6 groups (12-14 cells per group)/2 mice | W=0.8855 df=5 p=0.2953 |  |  |  |  |  |
| **Fig 4O** | **a.u.** | WT | mouse | 3 mice | W=0.9303 df=2 p=0.4898 | F=107.2 df1=2 df2=2 p=0.0.0185 | Two-tailed unpaired t-test | t=3.074 df=4 | P = 0.0372 | * |
|  |  | KO |  | 3 mice | W=0.8685 df=2 p=0.2913 |  |  |  |  |  |
| **Fig 4Q** | **Relative intensity (HDAC9/actin)** | WT | mouse | 3 mice | W=0.9810 df=2 p=0.7357 | F=0.8860 df1=2 df2=6 p=0.4601 | Repeated measurement ANOVA | F(2,6)=8.130 | P ≤ 0.05 | * |
|  |  | KO+saline |  | 3 mice | W=0.9757 df=2 p=0.7010 |  |  |  |  |  |
|  |  | KO+BHB |  | 3 mice | W=0.9479 df=2 p=0.5604 |  |  |  |  |  |
|  |  | KO+saline vs. WT |  |  |  |  | Tukey's multiple comparisons test |  | P = 0.0249 | * |
|  |  | KO+ saline vs. KO+BHB |  |  |  |  | Tukey's multiple comparisons test |  | P = 0.0374 | * |
|  |  | WT vs. KO+BHB |  |  |  |  | Tukey's multiple comparisons test |  | P = 0.9375 | ns |
| **Fig 4R** | **Relative Hdac9 mRNA levels** | WT | mouse | 3 mice | W=0.9943 df=2 p=0.8552 | F=0.2520 df1=2 df2=6 p=0.7851 | Repeated measurement ANOVA | F(2,6)=22.49 | P ≤ 0.01 | ** |
|  |  | KO+saline |  | 3 mice | W=0.9828 df=2 p=0.7488 |  |  |  |  |  |
|  |  | KO+BHB |  | 3 mice | W=0.8618 df=2 p=0.2725 |  |  |  |  |  |
|  |  | KO+saline vs. WT |  |  |  |  | Tukey's multiple comparisons test |  | P = 0.0116 | * |
|  |  | KO+ saline vs. KO+BHB |  |  |  |  | Tukey's multiple comparisons test |  | P = 0.0014 | ** |
|  |  | WT vs. KO+BHB |  |  |  |  | Tukey's multiple comparisons test |  | P = 0.1382 | ns |
| **Fig 5D** | **Relative intensity (HDAC9/actin)** | Control | mouse | 3 mice | W=0.9991 df=2 p=0.9424 | F=1.243 df1=2 df2=2 p=0.8916 | Two-tailed unpaired t-test | t=2.852 df=4 | P = 0.0463 | * |
|  |  | HDAC9-OE |  | 3 mice | W=0.9569 df=2 p=0.6008 |  |  |  |  |  |
| **Fig 5E** | **Relative Hdac9 mRNA levels** | Control | mouse | 3 mice | W=0.9513 df=2 p=0.5748 | F=11.93 df1=2 df2=2 p=0.1547 | Two-tailed unpaired t-test | t=3.738 df=4 | P = 0.0202 | * |
|  |  | HDAC9-OE |  | 3 mice | W=0.9491 df=2 p=0.5652 |  |  |  |  |  |
| **Fig 5G** | **Time in compartment (s)** | Control (S) | mouse | 7 mice | W=0.9597 df=6 p=0.8163 | F=1.595 df1=6 df2=6 p=0.5847 | Two-tailed unpaired t-test | t=5.398 df=12 | P = 0.0002 | *** |
|  |  | Control (E) |  | 7mice | W=0.9024 df=6 p=0.3457 |  |  |  |  |  |
|  |  | HDAC9-OE+saline (S) |  | 8 mice | Not all subgroups meets p＞0.05 |  | Mann-Whitney U test | U=9.500 | P = 0.0166 | * |
|  |  | HDAC9-OE+saline (E) |  | 8 mice |  |  |  |  |  |  |
|  |  | HDAC9-OE+BHB (S) |  | 5 mice | W=0.8814 df=4 p=0.3155 | F=1.326 df1=4 df2=4 p=0.7909 | Two-tailed unpaired t-test | t=-3.000 df=8 | P = 0.0171 | * |
|  |  | HDAC9-OE+BHB (E) |  | 5 mice | W=0.9359 df=4 p=0.6373 |  |  |  |  |  |
| **Fig 5H** | **Preference Score** | Control | mouse | 7 mice | W=0.9487 df=6 p=0.7182 | F=1.610 df1=2 df2=17 p=0.2290 | Kruskal-Wallis test | H=6.778 df=2 | P ≤ 0.05 | * |
|  |  | HDAC9-OE+saline |  | 8 mice | W=0.8716 df=7 p=0.1563 |  |  |  |  |  |
|  |  | HDAC9-OE+BHB |  | 5 mice | W=0.9316 df=4 p=0.6076 |  |  |  |  |  |
|  |  | HDAC9-OE+saline vs. Control |  |  |  |  | Dunn's multiple comparisons test |  | P = 0.0332 | * |
|  |  | HDAC9-OE +saline vs. HDAC9-OE+BHB |  |  |  |  | Dunn's multiple comparisons test |  | P ＞ 0.9999 | ns |
|  |  | HDAC9-OE+BHB vs. Control |  |  |  |  | Dunn's multiple comparisons test |  | P = 0.3351 | ns |
| **Fig 5I** | **Social interaction time (s)** | Control | mouse | 7 mice | W=0.9330 df=6 p=0.5770 | F=5.252 df1=2 df2=17 p=0.0176 | Kruskal-Wallis test | H=11.7 df=2 | P ≤ 0.01 | ** |
|  |  | HDAC9-OE+saline |  | 8 mice | W=0.8382 df=7 p=0.0722 |  |  |  |  |  |
|  |  | HDAC9-OE+BHB |  | 5mice | W=0.9052 df=4 p=0.4394 |  |  |  |  |  |
|  |  | HDAC9-OE+saline vs. Control |  |  |  |  | Dunn's multiple comparisons test |  | P = 0.0021 | ** |
|  |  | HDAC9-OE+saline vs. HDAC9-OE+BHB |  |  |  |  | Dunn's multiple comparisons test |  | P = 0.0435 | * |
|  |  | HDAC9-OE+BHB vs. Control |  |  |  |  | Dunn's multiple comparisons test |  | P = 0.5119 | ns |
| **Fig 5L** | **Frequency (Hz)** | Control | cells | 13 cells/2 mice | W=0.9603 df=12 p=0.7580 | F=5.086 df1=2 df2=32 p=0.0121 | Kruskal-Wallis test | H=11.69 df=2 | P ≤ 0.01 | ** |
|  |  | HDAC9-OE+saline |  | 11 cells/2 mice | W=0.9201 df=10 p=0.3191 |  |  |  |  |  |
|  |  | HDAC9-OE+BHB |  | 11 cells/2 mice | W=0.8921 df=10 p=0.1477 |  |  |  |  |  |
|  |  | HDAC9-OE+saline vs. Control |  |  |  |  | Dunn's multiple comparisons test |  | P = 0.0050 | ** |
|  |  | HDAC9-OE+saline vs. HDAC9-OE+BHB |  |  |  |  | Dunn's multiple comparisons test |  | P = 0.0159 | * |
|  |  | HDAC9-OE+BHB vs. Control |  |  |  |  | Dunn's multiple comparisons test |  | P ＞ 0.9999 | ns |
| **Fig 5M** | **Amplitude (pA)** | Control | cells | 13 cells/2 mice | W=0.9086 df=12 p=0.1756 | F=2.865 df1=2 df2=34 p=0.0708 | Repeated measurement ANOVA | F(2,32)=2.535 | P ≥ 0.05 | ns |
|  |  | HDAC9-OE+saline |  | 11 cells/2 mice | W=0.9580 df=10 p=0.7464 |  |  |  |  |  |
|  |  | HDAC9-OE+BHB |  | 11 cells/2 mice | W=0.8558 df=10 p=0.0508 |  |  |  |  |  |
|  |  | HDAC9-OE+saline vs. Control |  |  |  |  | Tukey's multiple comparisons test |  | P = 0.4036 | ns |
|  |  | HDAC9-OE+saline vs. HDAC9-OE+BHB |  |  |  |  | Tukey's multiple comparisons test |  | P = 0.0786 | ns |
|  |  | HDAC9-OE+BHB vs. Control |  |  |  |  | Tukey's multiple comparisons test |  | P = 0.5611 | ns |
| **Fig 5O** | **Relative intensity**  **(GLUR1/ACTIN)** | Control | mouse | 5 mice | W=0.9591 df=4 p=0.8019 | F=0.9291 df1=2 df2=12 p=0.4216 | Repeated measurement ANOVA | F(2,12)=8.031 | P ≤ 0.01 | ** |
|  |  | HDAC9-OE+saline |  | 5 mice | W=0.7857 df=4 p=0.0616 |  |  |  |  |  |
|  |  | HDAC9-OE+BHB |  | 5 mice | W=0.8698 df=4 p=0.2658 |  |  |  |  |  |
|  |  | HDAC9-OE+saline vs. Control |  |  |  |  | Bonferroni's multiple comparisons test |  | P = 0.0018 | ** |
|  |  | HDAC9-OE+saline vs. HDAC9-OE+BHB |  |  |  |  | Bonferroni's multiple comparisons test |  | P = 0.0340 | * |
|  |  | HDAC9-OE+BHB vs. Control |  |  |  |  | Bonferroni's multiple comparisons test |  | P = 0.1383 | ns |
| **Fig 5Q** | **Relative intensity**  **(GLUR2/ACTIN)** | Control | mouse | 4 mice | W=0.9980 df=3 p=0.9936 | F=3.945 df1=2 df2=9 p=0.0588 | Kruskal-Wallis test | H=5.115 df=2 | P ≥ 0.05 | ns |
|  |  | HDAC9-OE+saline |  | 4 mice | W=0.8920 df=3 p=0.3926 |  |  |  |  |  |
|  |  | HDAC9-OE+BHB |  | 4 mice | W=0.9956 df=3 p=0.9842 |  |  |  |  |  |
|  |  | HDAC9-OE+saline vs. Control |  |  |  |  | Bonferroni's multiple comparisons test |  | P = 0.0215 | * |
|  |  | HDAC9-OE+saline vs. HDAC9-OE+BHB |  |  |  |  | Bonferroni's multiple comparisons test |  | P = 0.2087 | ns |
|  |  | HDAC9-OE+BHB vs. Control |  |  |  |  | Bonferroni's multiple comparisons test |  | P = 0.1844 | ns |
| **Fig 5S** | **Relative intensity**  **(SHANK3/ACTIN)** | Control | mouse | 3 mice | W=1.000 df=2 p＞0.9999 | F=0.3721 df1=2 df2=6 p=0.0.7041 | Repeated measurement ANOVA | F(2,6)=12.20 | P ≤ 0.01 | ** |
|  |  | HDAC9-OE+saline |  | 3 mice | W=0.9504 df=2 p=0.5710 |  |  |  |  |  |
|  |  | HDAC9-OE+BHB |  | 3 mice | W=0.9108 df=2 p=0.4208 |  |  |  |  |  |
|  |  | HDAC9-OE+saline vs. Control |  |  |  |  | Tukey's multiple comparisons test |  | P = 0.0073 | ** |
|  |  | HDAC9-OE+saline vs. HDAC9-OE+BHB |  |  |  |  | Tukey's multiple comparisons test |  | P = 0.0309 | * |
|  |  | HDAC9-OE+BHB vs. Control |  |  |  |  | Tukey's multiple comparisons test |  | P = 0.4398 | ns |
| **Fig 6C** | **Number of Interactions** | WT | cells | 10 cells/3 mice | Not all subgroups meets p＞0.05 |  | Two-way RM ANOVA | F(2, 31)=16.66 | P ≤ 0.0001 | **** |
|  |  | KO+saline |  | 11 cells/4 mice |  |  |  |  |  |  |
|  |  | KO+BHB |  | 13 cells/4 mice |  |  |  |  |  |  |
|  |  | WT vs. KO+saline |  |  |  |  | Tukey's multiple comparisons test |  | P ≤ 0.0001 | **** |
|  |  | KO+saline vs. KO+BHB |  |  |  |  | Tukey's multiple comparisons test |  | P = 0.1142 | ns |
|  |  | WT vs. KO+BHB |  |  |  |  | Tukey's multiple comparisons test |  | P ≤ 0.0001 | **** |
| **Fig 6E** | **Total spine density**  **(no. per 10 μm)** | WT | dendrites | 8 dendrites/3 mice | Not all subgroups meets p＞0.05 |  | Kruskal-Wallis test | H=13.08 df=2 | P ≤ 0.01 | ** |
|  |  | KO+saline |  | 19 dendrites/3 mice |  |  |  |  |  |  |
|  |  | KO+BHB |  | 19 dendrites/3 mice |  |  |  |  |  |  |
|  |  | WT vs. KO+saline |  |  |  |  | Dunn's multiple comparisons test |  | P = 0.0058 | ** |
|  |  | KO+saline vs. KO+BHB |  |  |  |  | Dunn's multiple comparisons test |  | P = 0.0108 | * |
|  |  | WT vs. KO+BHB |  |  |  |  | Dunn's multiple comparisons test |  | P﹥0.9999 | ns |
| **Fig 6F** | **Mushroom spine density**  **(no. per 10 μm)** | WT | dendrites | 8 dendrites/3 mice | Not all subgroups meets p＞0.05 |  | Kruskal-Wallis test | H=9.535 df=2 | P ≤ 0.01 | ** |
|  |  | KO+saline |  | 19 dendrites/3 mice |  |  |  |  |  |  |
|  |  | KO+BHB |  | 19 dendrites/3 mice |  |  |  |  |  |  |
|  |  | WT vs. KO+saline |  |  |  |  | Dunn's multiple comparisons test |  | P = 0.0032 | ** |
|  |  | KO+saline vs. KO+BHB |  |  |  |  | Dunn's multiple comparisons test |  | P = 0.0468 | * |
|  |  | WT vs. KO+BHB |  |  |  |  | Dunn's multiple comparisons test |  | P = 0.1571 | ns |
| **Fig 6I** | **Frequency (Hz)** | WT | cells | 7 cells/2 mice | W=0.8834 df=6 p=0.2421 | F=1.865 df1=2 df2=25 p=0.5098 | Repeated measurement ANOVA | F(2,25)=12.51 | P ≤ 0.001 | *** |
|  |  | KO+saline |  | 11 cells/2 mice | W=0.9621 df=9 p=0.5070 |  |  |  |  |  |
|  |  | KO+BHB |  | 10 cells/2 mice | W=0.9075 df=9 p=0.2640 |  |  |  |  |  |
|  |  | WT vs. KO+saline |  |  |  |  | Bonferroni's multiple comparisons test |  | P = 0.0664 | ns |
|  |  | KO+saline vs. KO+BHB |  |  |  |  | Bonferroni's multiple comparisons test |  | P= 0.0001 | **** |
|  |  | WT vs. KO+BHB |  |  |  |  | Bonferroni's multiple comparisons test |  | P = 0.1060 | ns |
| **Fig 6J** | **Amplitude (pA)** | WT | cells | 7 cells/2 mice | W=0.9247 df=6 p=0.5070 | F=5.235 df1=2 df2=25 p=0.0126 | Kruskal-Wallis test | H=14.25 df=2 | P ≤ 0.001 | *** |
|  |  | KO+saline |  | 11 cells/2 mice | W=0.9193 df=10 p=0.3132 |  |  |  |  |  |
|  |  | KO+BHB |  | 10 cells/2 mice | W=0.8594 df=9 p=0.0750 |  |  |  |  |  |
|  |  | WT vs. KO+saline |  |  |  |  | Dunn's multiple comparisons test |  | P= 0.0011 | ** |
|  |  | KO+saline vs. KO+BHB |  |  |  |  | Dunn's multiple comparisons test |  | P= 0.0236 | * |
|  |  | WT vs. KO+BHB |  |  |  |  | Dunn's multiple comparisons test |  | P = 0.7650 | ns |
| **Fig 6M** | **△F/F** | WT | cells | 133 cells/3 experiments | Not all subgroups meets p＞0.05 |  | Kruskal-Wallis test | H=14.25 df=2 | P ≤ 0.001 | *** |
|  |  | KO+saline |  | 140 cells/3 experiments |  |  |  |  |  |  |
|  |  | KO+BHB |  | 103 cells/3 experiments |  |  |  |  |  |  |
|  |  | WT vs. KO+saline |  |  |  |  | Dunn's multiple comparisons test |  | P ≤ 0.0001 | **** |
|  |  | KO+saline vs. KO+BHB |  |  |  |  | Dunn's multiple comparisons test |  | P ≤ 0.0001 | **** |
|  |  | WT vs. KO+BHB |  |  |  |  | Dunn's multiple comparisons test |  | P ≤ 0.0001 | **** |
| **Fig 6O** | **Relative intensity**  **(GLUR1/ACTIN)** | WT | mouse | 6 mice | W=0.9415 df=5 p=0.6709 | F=5.235 df1=2 df2=15 p=0.0126 | Repeated measurement ANOVA | F(2,15)=3.705 | P ≤ 0.05 | * |
|  |  | KO+saline |  | 6 mice | W=0.8158 df=5 p=0.0811 |  |  |  |  |  |
|  |  | KO+BHB |  | 6 mice | W=0.9272 df=5 p=0.5590 |  |  |  |  |  |
|  |  | WT vs. KO+saline |  |  |  |  | Bonferroni's multiple comparisons test |  | P = 0.0259 | * |
|  |  | KO+saline vs. KO+BHB |  |  |  |  | Bonferroni's multiple comparisons test |  | P = 0.0421 | * |
|  |  | WT vs. KO+BHB |  |  |  |  | Bonferroni's multiple comparisons test |  | P = 0.8061 | ns |
| **Fig 6Q** | **Relative intensity**  **(GLUR2/ACTIN)** | WT | mouse | 6 mice | W=0.9056 df=5 p=0.4083 | F=0.1502 df1=2 df2=15 p=0.8618 | Repeated measurement ANOVA | F(2,15)=4.527 | P ≤ 0.05 | * |
|  |  | KO+saline |  | 6 mice | W=0.8901 df=5 p=0.3186 |  |  |  |  |  |
|  |  | KO+BHB |  | 6 mice | W=0.9514 df=5 p=0.7512 |  |  |  |  |  |
|  |  | WT vs. KO+saline |  |  |  |  | Bonferroni's multiple comparisons test |  | P = 0.0099 | ** |
|  |  | KO+saline vs. KO+BHB |  |  |  |  | Bonferroni's multiple comparisons test |  | P = 0.0658 | ns |
|  |  | WT vs. KO+BHB |  |  |  |  | Bonferroni's multiple comparisons test |  | P = 0.3493 | ns |
| **Fig 7D** | **Time in compartment (s)** | KO+Vehcile (S) | mouse | 6 mice | W=0.8909 df=5 p=0.3229 | F=1.635 df1=5 df2=5 p=0.6026 | Two-tailed unpaired t-test | t=-0.2853 df=10 | P = 0.7812 | ns |
|  |  | KO+Vehcile (E) |  | 6 mice | W=0.9239 df=5 p=0.5337 |  |  |  |  |  |
|  |  | KO+TMP269 (S) |  | 8 mice | W=0.8984 df=5 p=0.2793 | F=1.301 df1=7 df2=7 p=0.7372 | Two-tailed unpaired t-test | t=-5.366 df=14 | P ≤ 0.0001 | **** |
|  |  | KO+TMP269 (E) |  | 8 mice | W=0.9682 df=5 p=0.8835 |  |  |  |  |  |
| **Fig 7E** | **Social interaction time(s)** | KO+Vehcile | mouse | 6 mice | W=0.9250 df=5 p=0.5423 | F=3.352 df1=5 df2=7 p=0.2017 | Two-tailed unpaired t-test | t=-2.414 df=12 | P = 0.0327 | * |
|  |  | KO+TMP269 |  | 8 mice | W=0.9164 df=5 p=0.4014 |  |  |  |  |  |
| **Fig 7F** | **Preference Score** | KO+Vehcile | mouse | 6 mice | W=0.9649 df=5 p=0.8568 | F=3.836 df1=5 df2=7 p=0.1580 | Two-tailed unpaired t-test | t=-3.163 df=12 | P = 0.0082 | ** |
|  |  | KO+TMP269 |  | 8 mice | W=0.9274 df=7 p=0.4928 |  |  |  |  |  |
| **Fig 7G** | **time in center zone (s)** | KO+Vehcile | mouse | 6 mice | W=0.8854 df=5 p=0.2950 | F=1.472 df1=5 df2=7 p=0.6932 | Two-tailed unpaired t-test | t=-0.3563 df=12 | P = 0.7278 | ns |
|  |  | KO+TMP269 |  | 8 mice | W=0.8630 df=5 p=0.1287 |  |  |  |  |  |
| **Fig 7H** | **total distance (cm)** | KO+Vehcile | mouse | 6 mice | W=0.9565 df=5 p=0.7923 | F=1.896 df1=5 df2=7 p=0.4990 | Two-tailed unpaired t-test | t=-0.1270 df=12 | P = 0.9011 | ns |
|  |  | KO+TMP269 |  | 8 mice | W=0.9471 df=7 p=0.6815 |  |  |  |  |  |
| **Fig 7J** | **Number of intersections** | KO+Vehcile | cells | 6 cells/2 mice | Not all subgroups meets p＞0.05 |  | Two-way ANOVA | F(1,10)=13.30 | P ≤ 0.001 | *** |
|  |  | KO+TMP269 |  | 6 cells/4 mice |  |  |  |  |  |  |
|  |  | KO+Vehcile |  |  |  |  | Interaction | F(44, 440) = 4.572 | P ≤ 0.0001 | **** |
|  |  | KO+TMP269 |  |  |  |  |  |  |  |  |
| **Fig 7L** | **Total spine density**  **(no. per 10 μm)** | KO+Vehcile | dendrites | 28 dendrites/ 2 mice | W=0.9675 df=28 p=0.5157 | F=1.595 df1=28 df2=23 p=0.2478 | Two-tailed unpaired t-test | t=-4.348 df=49 | P ≤ 0.0001 | **** |
|  |  | KO+TMP269 |  | 23 dendrites/4 mice | W=0.9805 df=23 p=0.9138 |  |  |  |  |  |
| **Fig 7M** | **Mushroom spine density**  **(no. per 10 μm)** | KO+Vehcile | dendrites | 28 dendrites/ 2 mice | Not all subgroups meets p＞0.05 |  | Mann-Whitney U test | U=113.5 | P ≤ 0.0001 | **** |
|  |  | KO+TMP269 |  | 23 dendrites/4 mice |  |  |  |  |  |  |
| **Fig 7O** | **Frequency (Hz)** | KO+Vehcile | cells | 16 cells/2 mice | Not all subgroups meets p＞0.05 |  | Mann-Whitney U test | U=52 | P = 0.0006 | *** |
|  |  | KO+TMP269 |  | 19 cells/3 mice |  |  |  |  |  |  |
| **Fig 7P** | **Amplitude (pA)** | KO+Vehcile | cells | 16 cells/2 mice | Not all subgroups meets p＞0.05 |  | Mann-Whitney U test | U=90 | P = 0.0406 | * |
|  |  | KO+TMP269 |  | 19 cells/3 mice |  |  |  |  |  |  |
| **Fig 7S** | **△F/F** | KO+Vehcile | cells | 140 cells/3 experiments | Not all subgroups meets p＞0.05 |  | Mann-Whitney U test | U=9510 | P ≤ 0.0001 | **** |
|  |  | KO+TMP269 |  | 249 cells/3 experiments |  |  |  |  |  |  |
| **Fig EV1C** | **Time in center (S)** | Control | mouse | 8 mice | W=0.9228 df=7 p=0.4527 | F=1.088 df1=7 df2=8 p=0.9248 | Two-tailed unpaired t-test | t=-0.07125 df=15 | P = 0.9441 | ns |
|  |  | HDAC9-OE |  | 9 mice | W=0.8768 df=8 p=0.1454 |  |  |  |  |  |
| **Fig EV1D** | **Total distance (cm)** | Control | mouse | 8 mice | W=0.9318 df=7 p=0.5325 | F=1.890 df1=7 df2=8 p=0.3916 | Two-tailed unpaired t-test | t=-3.270 df=15 | P = 0.0052 | ** |
|  |  | HDAC9-OE |  | 9 mice | W=0.8398 df=8 p=0.0574 |  |  |  |  |  |
| **Fig EV1E** | **Grooming duration (s)** | Control | mouse | 6 mice | W=0.9107 df=5 p=0.4408 | F=2.434 df1=5 df2=7 p=0.3447 | Two-tailed unpaired t-test | t=-0.07067 df=12 | P = 0.9448 | ns |
|  |  | HDAC9-OE |  | 8 mice | W=0.9687 df=7 p=0.8878 |  |  |  |  |  |
| **Fig EV3B** | **Total length**  **of dendrites (μm)** | KO+saline | cells | 25 cells | W=0.9211 df=24 p=0.0541 | F=1.488 df1=24 df2=20 p=0.3702 | Two-tailed unpaired t-test | t=-2.246 df=44 | P = 0.0298 | * |
|  |  | KO+BHB |  | 21 cells | W=0.9218 df=20 p=0.0941 |  |  |  |  |  |
| **Fig EV4B** | **Relative fluorescence intensity** | WT+DMSO | slices | 3 slices | W=0.9727 df=2 p=0.6830 | F=0.3318 df1=3 df2=8 p=0.8028 | Repeated measurement ANOVA | F(3,8)=7.126 | P ≤ 0.01 | ** |
|  |  | WT+Bay K8644 |  | 3 slices | W=0.9929 df=2 p=0.8390 |  |  |  |  |  |
|  |  | KO+DMSO |  | 3 slices | W=0.9783 df=2 p=0.7174 |  |  |  |  |  |
|  |  | KO+Bay K8644 |  | 3 slices | W=0.9892 df=2 p=0.8012 |  |  |  |  |  |
|  |  | WT+DMSO vs. WT+Bay K8644 |  |  |  |  | Bonferroni's multiple comparisons test |  | P = 0.4739 | ns |
|  |  | WT+DMSO vs. KO+DMSO |  |  |  |  | Bonferroni's multiple comparisons test |  | P = 0.0021 | ** |
|  |  | WT+DMSO vs. KO+Bay K8644 |  |  |  |  | Bonferroni's multiple comparisons test |  | P = 0.2338 | ns |
|  |  | KO+DMSO vs. WT+Bay K8644 |  |  |  |  | Bonferroni's multiple comparisons test |  | P = 0.0163 | * |
|  |  | KO+Bay K8644 vs. WT+Bay K8644 |  |  |  |  | Bonferroni's multiple comparisons test |  | P = 0.8964 | ns |
|  |  | KO+DMSO vs. KO+Bay K8644 |  |  |  |  | Bonferroni's multiple comparisons test |  | P = 0.0133 | * |
| **Fig EV4C** | **Relative Hdac9**  **mRNA expression** | WT+DMSO | wells | 5 wells | W=0.8882 df=4 p=0.3480 | F=0.9425 df1=2 df2=16 p=0.4433 | Repeated measurement ANOVA | F(3,16)=9.873 | P ≤ 0.001 | *** |
|  |  | WT+Bay K8644 |  | 5 wells | W=0.9205 df=4 p=0.5332 |  |  |  |  |  |
|  |  | KO+DMSO |  | 5 wells | W=0.9253 df=4 p=0.5649 |  |  |  |  |  |
|  |  | KO+Bay K8644 |  | 5 wells | W=0.8607 df=4 p=0.2309 |  |  |  |  |  |
|  |  | WT+DMSO vs. WT+Bay K8644 |  |  |  |  | Tukey's multiple comparisons test |  | P = 0.8847 | ns |
|  |  | WT+DMSO vs. KO+DMSO |  |  |  |  | Tukey's multiple comparisons test |  | P = 0.0006 | *** |
|  |  | WT+DMSO vs. KO+Bay K8644 |  |  |  |  | Tukey's multiple comparisons test |  | P = 0.3686 | ns |
|  |  | KO+DMSO vs. WT+Bay K8644 |  |  |  |  | Tukey's multiple comparisons test |  | P = 0.0028 | ** |
|  |  | KO+Bay K8644 vs. WT+Bay K8644 |  |  |  |  | Tukey's multiple comparisons test |  | P = 0.7808 | ns |
|  |  | KO+DMSO vs. KO+Bay K8644 |  |  |  |  | Tukey's multiple comparisons test |  | P = 0.0190 | * |
